# Supplementary material for: Image-based drug screening combined with molecular profiling identifies signatures and drivers of therapy resistance in pediatric AML
Source: Cell Rep Med. 2025 Aug 20;6(9):102304. doi: 10.1016/j.xcrm.2025.102304 (PMC12490222; doi:10.1016/j.xcrm.2025.102304)
Supplement: Document S1. Figures S1–S9 and Tables S1 and S2 [file mmc1.pdf]

Cell Reports Medicine, Volume *6*

Supplemental information

Image-based drug screening combined with molecular profiling identifies signatures and drivers of therapy resistance in pediatric AML

Ben Haladik, Margarita Maurer-Granofszky, Peter Zoescher, Raul Jimenez-Heredia, Alexandra Frohne, Anna Segarra-Roca, Chloe Casey, Felix Kartnig, Sarah Giuliani, Christina Rashkova, Peter Repiscak, Michael N. Dworzak, Giulio Superti-Furga, and Kaan Boztug

Supplementary Figures

Figure S1

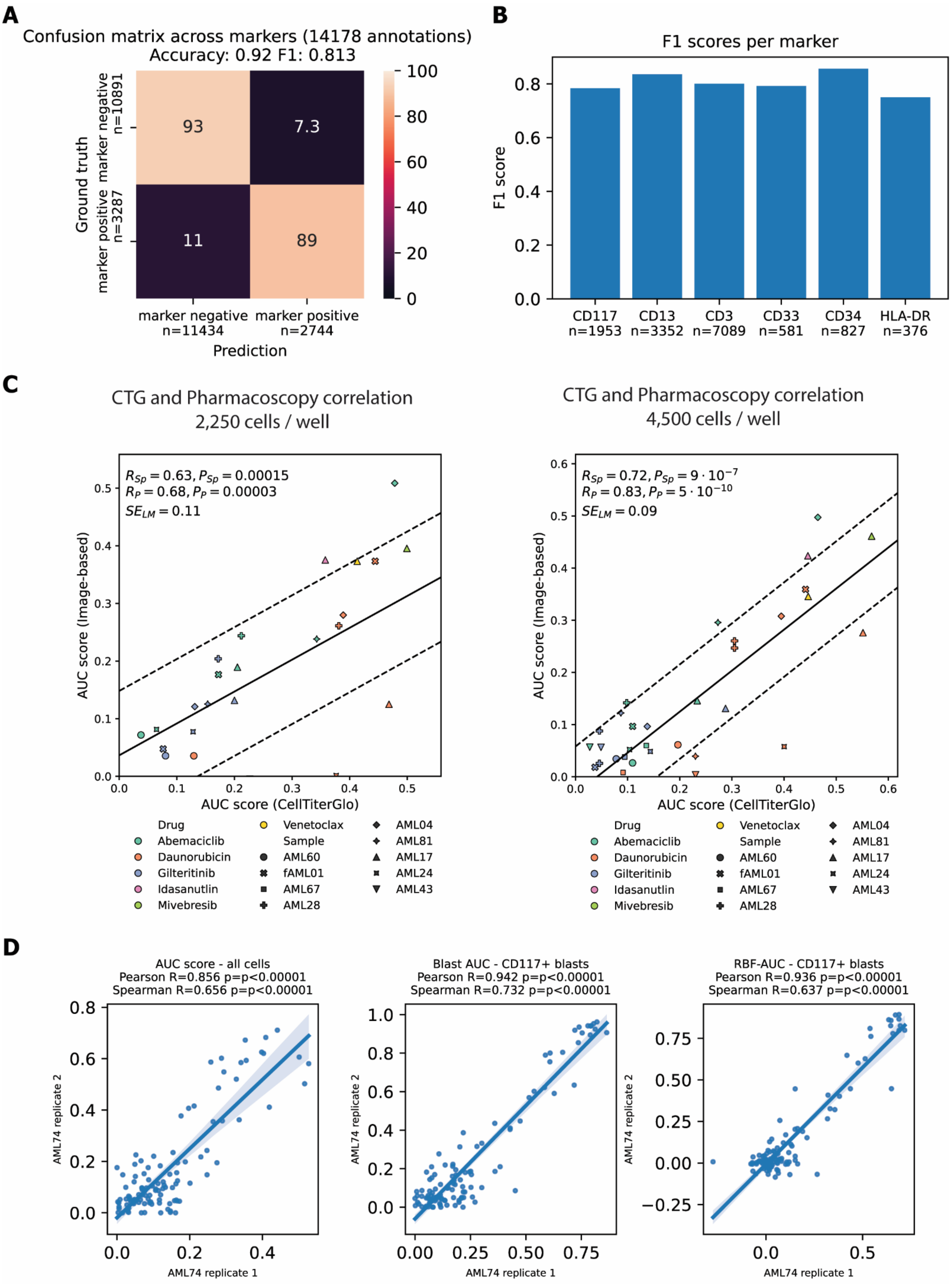

Figure S1: Pharmacoscopy technical benchmarkings. Related to Figure 1

A Confusion matrix for marker-positivity across all cells in the testing dataset for the celltype model. **B** Bargraph of F1 scores per surface antigen in the testing dataset. The bottom number indicates the number of cells **C** Left: Correlation between CTG inhibition scores and Pharmacoscopy inhibition scores for 6 drugs and 9 samples for a cell-density of 2.250 cells per well. Right: same as left, but with 4.500 cells per well. **D** Correlation between replicates across readouts for the AUC score on all cells (left), target cells only (middle) and the RBF-AUC score (right).

Figure S2

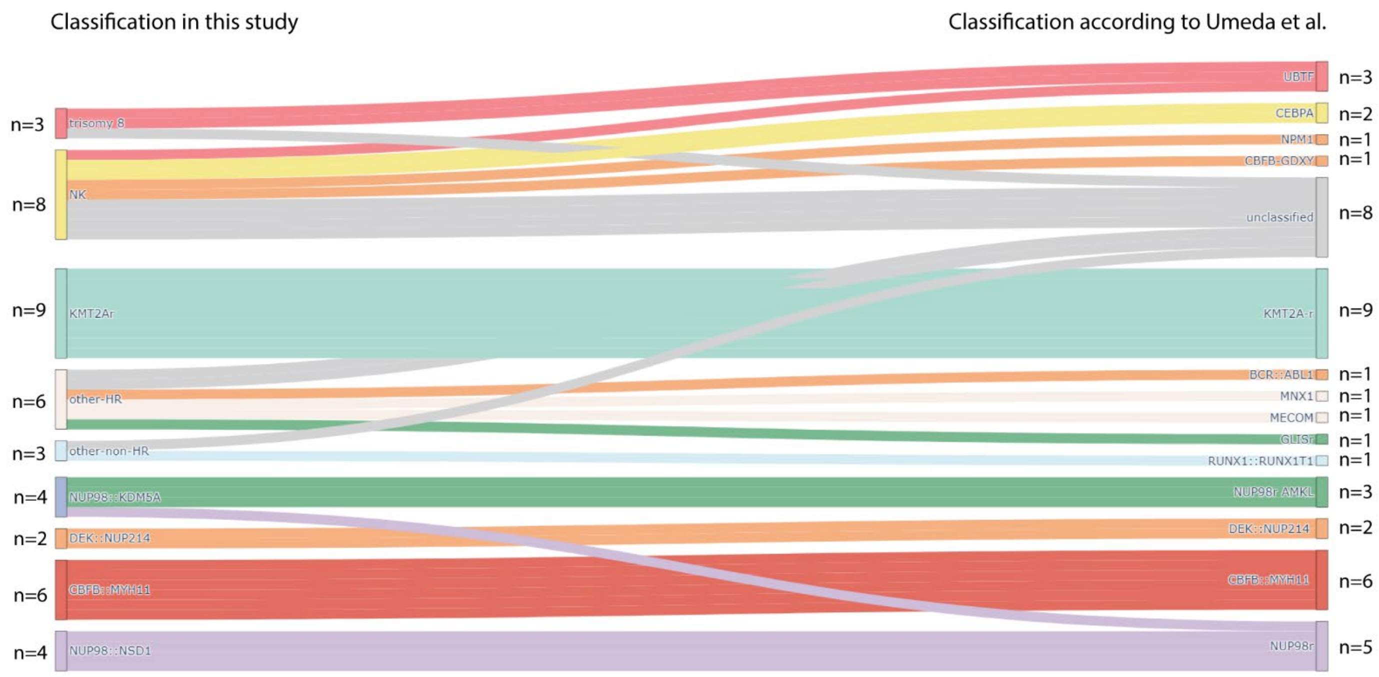

Figure S2: Molecular re-classification of our cohort. Related to Figure 2

Sankey chart indicating matchings between the classification in this study for the n=45 samples in our cohort and the classification in Umeda et al.

Figure S3

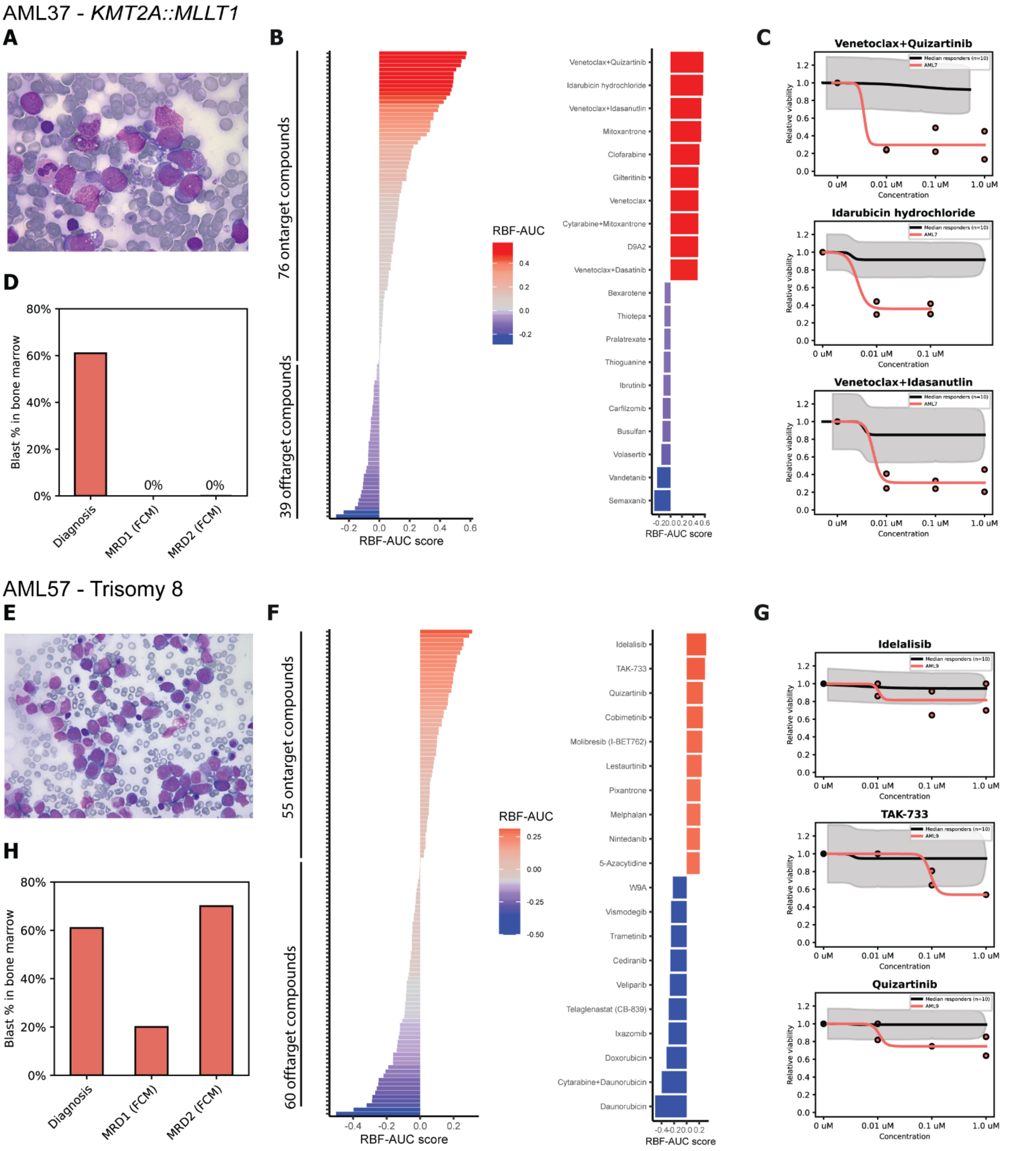

Figure S3: Case vignettes illustrating our drug sensitivity profiling reporting format. Related to Figure 3

A Bone marrow smear of sample AML37 (good responder). The patient had a monoblastic M5a phenotype and a *KMT2A::MLLT1* fusion. **B** Left: Bar chart of RBF-AUC scores for all tested compounds. Right: Bar chart of RBF-AUC scores for the 10 compounds with the highest and lowest RBF-AUC scores respectively and the induction regimen drugs Idarubicin and Mitoxantrone among the top 10 compounds. **C** Dose response curves for the patient (red) and 10 patients with RBF-AUC scores closest to the median score for the respective drug. Lines indicate the fit of the dose response curve. Dots indicate blast viability relative to the average of the DMSO control for each replicate after QC. The shaded grey area indicates the standard deviation around the curve fit for the 10 median responders. **D** Barplots of blast percentages at diagnosis and at MRD time points after induction 1 and 2 as determined by flow-cytometry (FCM) indicating complete response after the first induction cycle. **E** Bone marrow smear of sample AML57 (poor responder). The patient had an M2 phenotype, trisomy and mutations in *NRAS*, *WT1*, and *FLT3* **F** Barplots of RBF-AUC scores analogous with induction compounds Daunorubicin, Doxorubicin and the combination of

Cytarabine and Daunorubicin among the lowest scoring drugs analogous to **B**. **G** Dose response curves for top 3 drugs analogous to **C.** **H** Barplots of blast percentages at diagnosis and at MRD time points analogous to **D** indicating highly resistant disease.

Figure S4: Clustering analysis of chemosensitivities

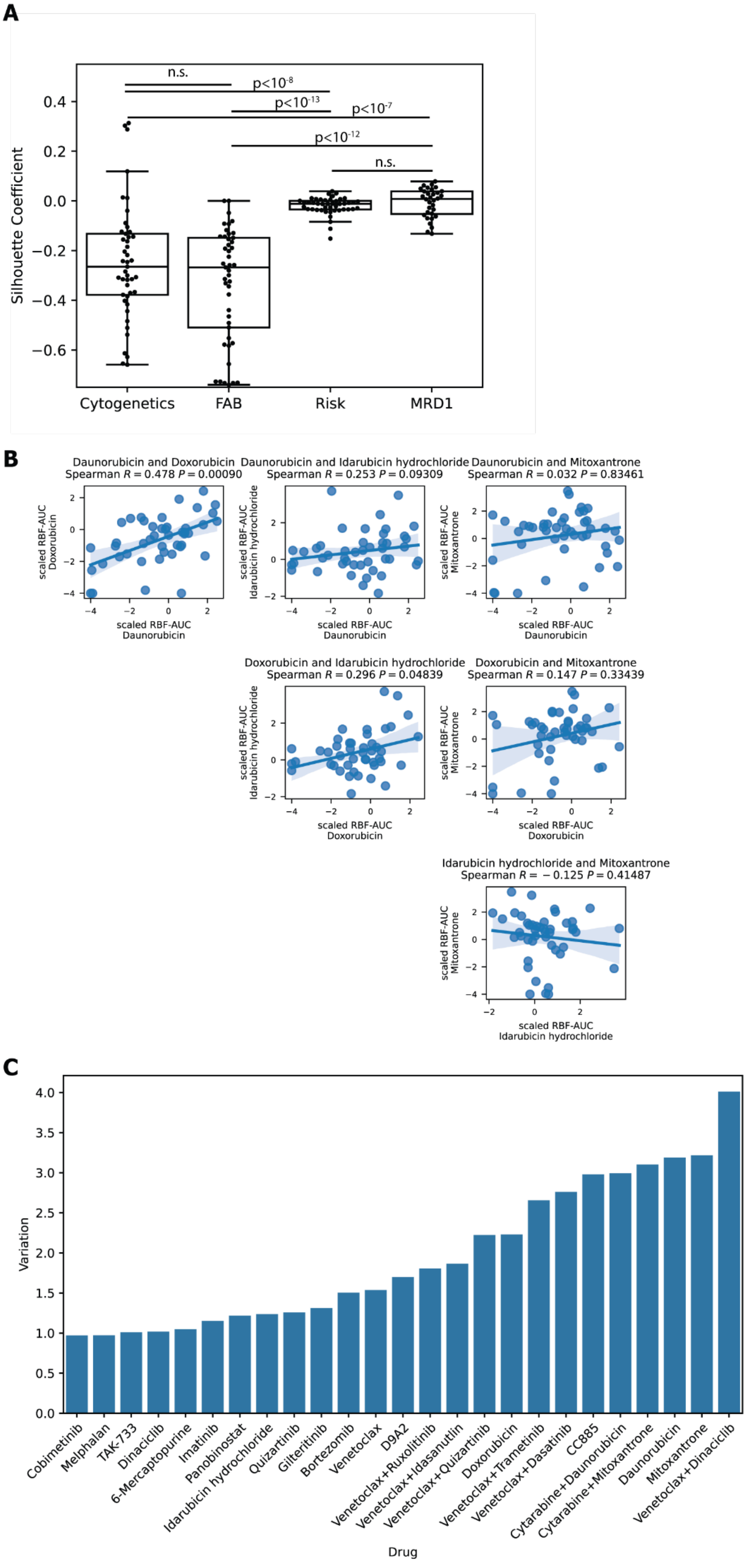

Figure S4: Clustering analysis of chemosensitivities. Related to Figure 3

A Silhouette coefficients of drug sensitivities per sample based on sample classification by cytogenetic group (n=45), FAB classification (n=45), risk group (n=45), and MRD status (n=32) respectively. P-values calculated with Mann-Whitney-U test. Dots indicate individual samples. Boxes represent quartiles. Whiskers extend to points that are within 1.5 interquartile ranges. **B** Correlations of RBF-AUC scores for selected chemotherapeutics for the n=45 samples profiled in this study. Individual dots indicate the scaled RBF-AUC score for individual samples and the respective compounds. Lines indicate a linear model fit. Shaded areas around lines indicate the 95% confidence interval. **C** Variation per compound for the 25 most variable compounds.

Figure S5

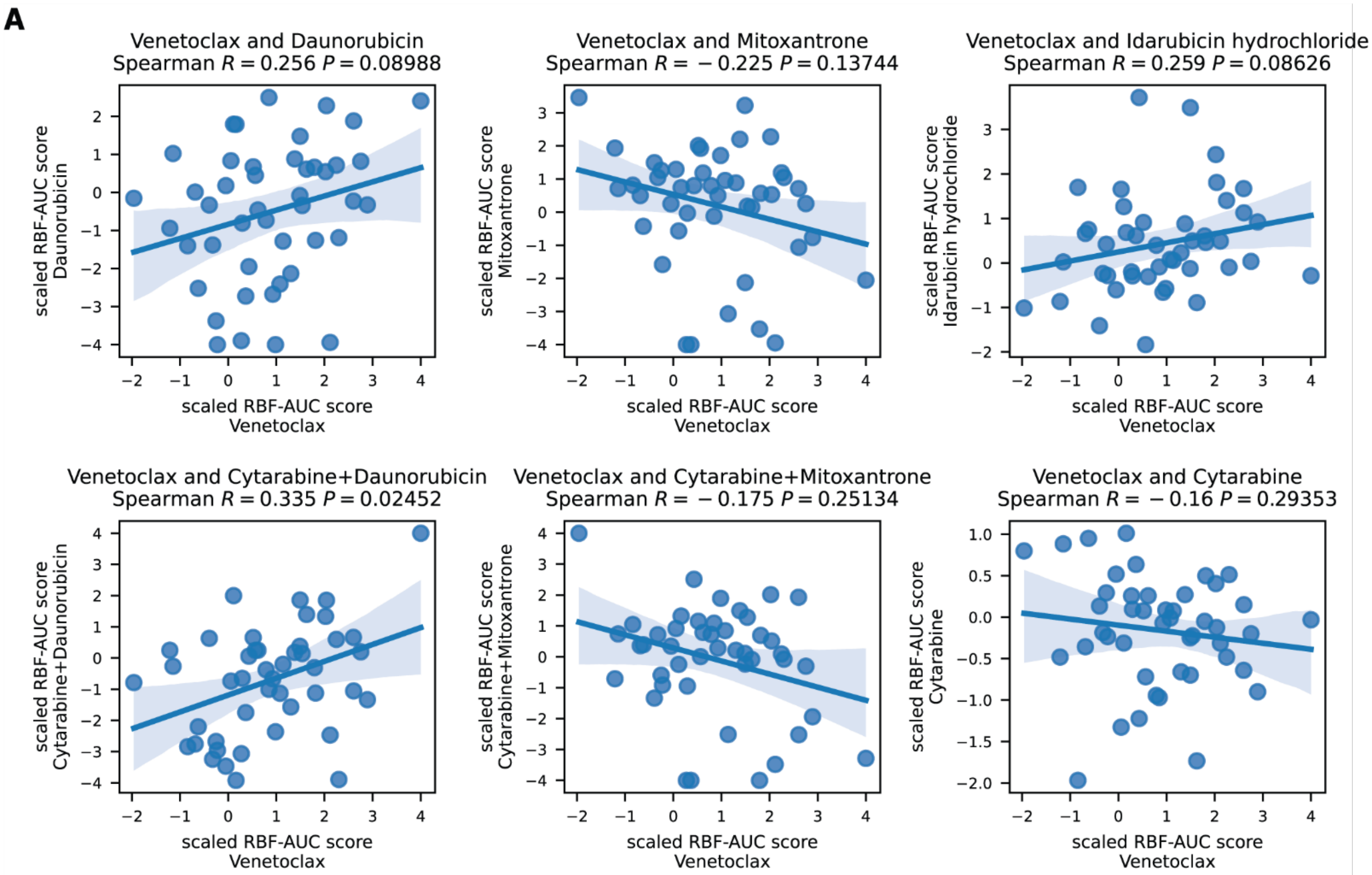

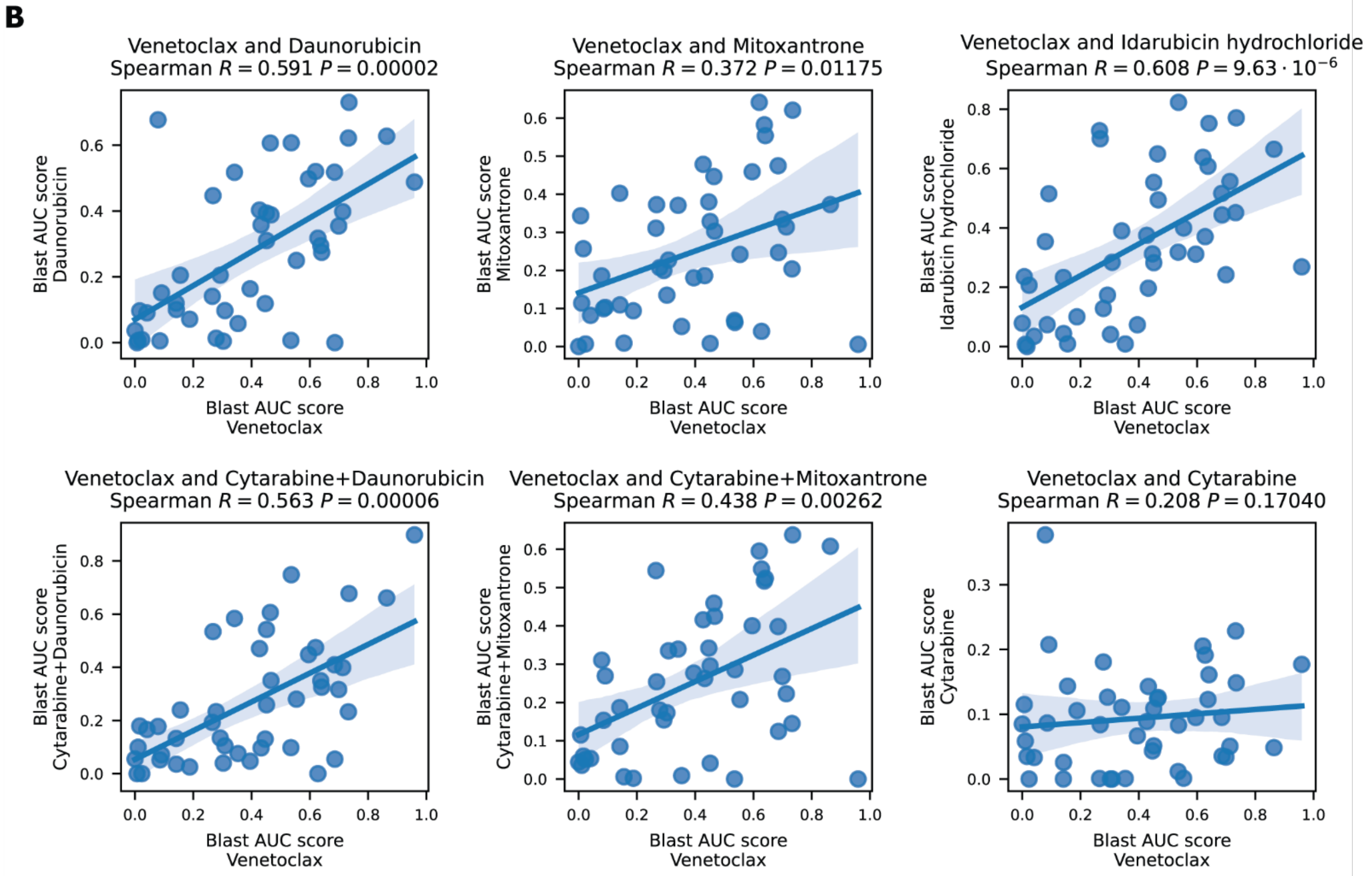

Figure S5: Correlation of Venetoclax and selected chemotherapeutic agents. Related to Figure 3

A Scatterplots and Spearman correlations between Venetoclax on the horizontal axes and selected chemotherapy compounds on the vertical axes for n=45 samples in the cohort. Individual dots indicate the scaled RBF-AUC score for individual samples and

the respective compounds. Lines indicate a linear model fit. Shaded areas around lines indicate the 95% confidence interval. **B** Same as A, but dots indicate the absolute AUC score that only reflects responses of the blast population (Methods).

Figure S6

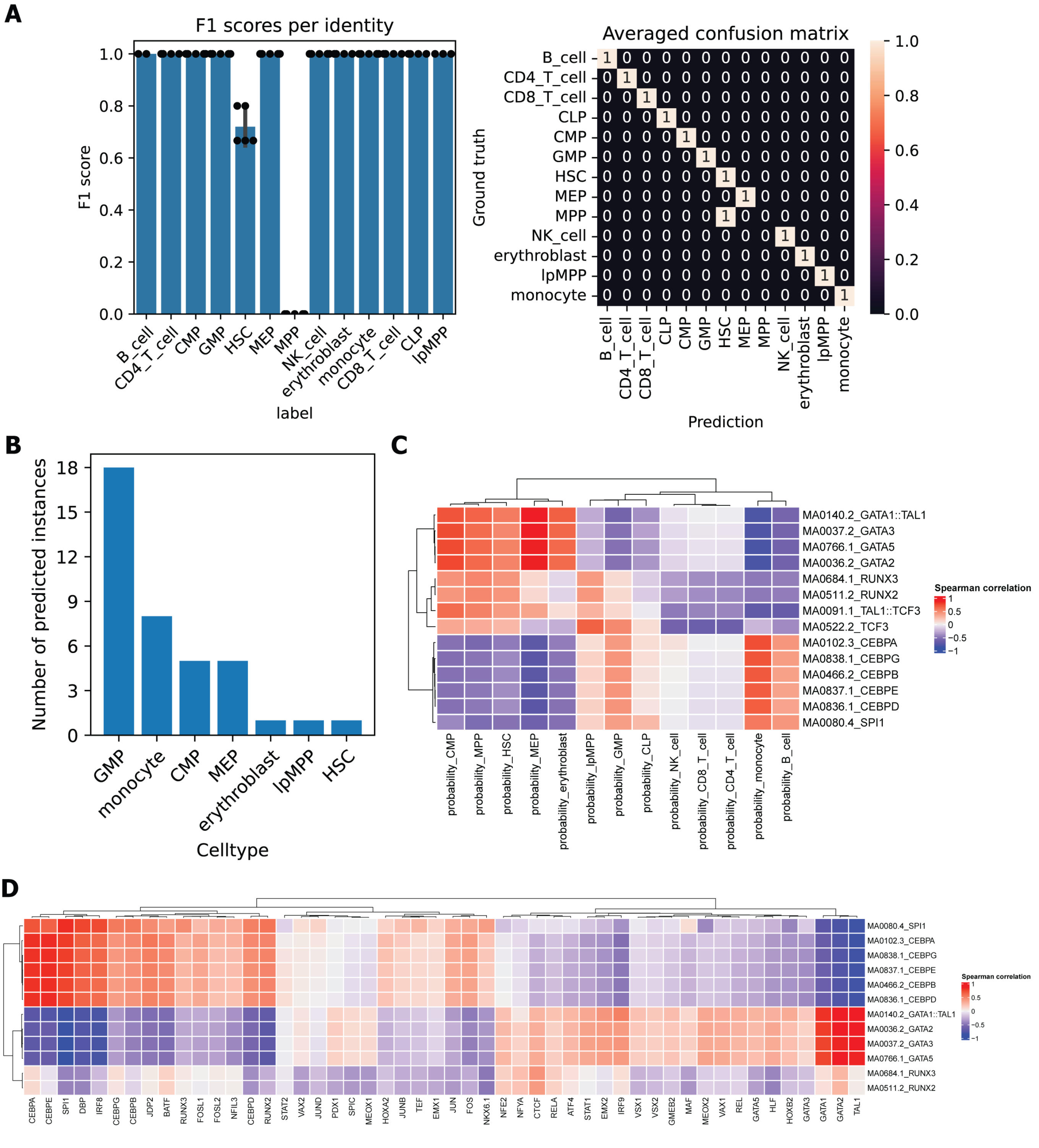

Figure S6: Mapping pedAML samples to their most similar healthy cell-types. Related to Figure 5

A Performance of the support vector classifier. Left: F1 scores per run and celltype in 5-fold cross-validation. Dots indicate F1 scores per run. Bar heights indicate mean F1 scores over all folds. Error bars indicate 95% confidence intervals. Right: Averaged confusion matrix over all runs. **B** Numbers of predicted healthy cell-types for samples in the ExTrAct-cohort. **C** Clustered heatmap of Spearman correlations for predicted probabilities of healthy cell-types and chromVAR deviation scores of key hematopoietic transcription factors **D** Spearman rank correlation for chromVAR scores for TF motifs from Supplementary Figure S4B and normalized expression levels of key hematopoietic transcription factors

Figure S7

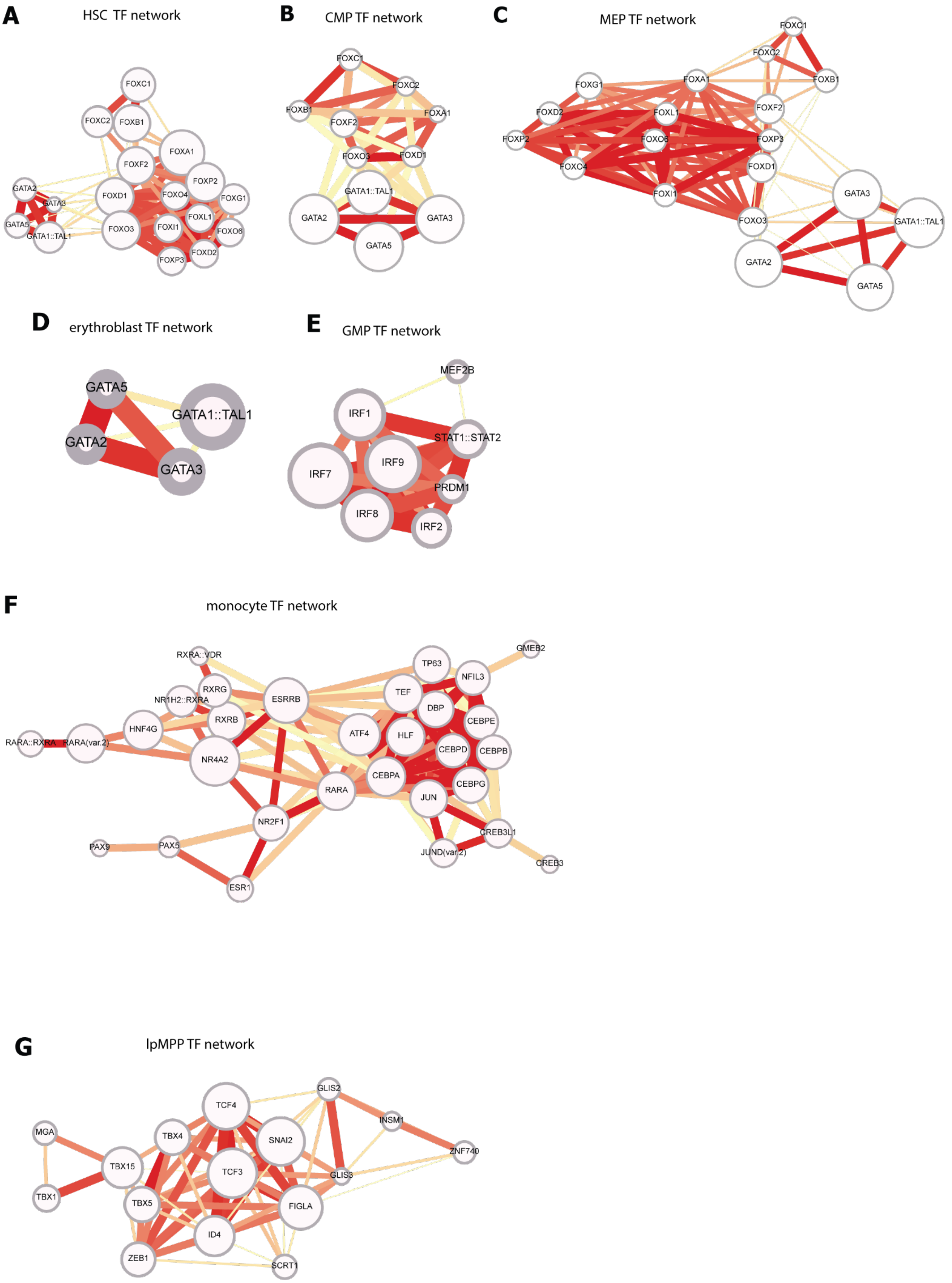

Figure S7: Transcription factor activity networks for predicted cell states. Related to Figure 5

Networks represent the largest connected component correlating TF-activities that are significantly associated with the respective cell states for the n=38 samples with ATAC-seq data after QC. Node sizes are proportional to correlation between TF activity and

cell state probability. Edges represent Spearman correlations of at least 0.8 between individual TF activities (STAR methods). Edge widths and colors are proportional to correlation value. **A** HSC TF network. **B** CMP TF network. **C** MEP TF network. **D** erythroblast TF network. **E** GMP TF network. **F** monocyte TF network. **G** lpMPP TF network.

Figure S8

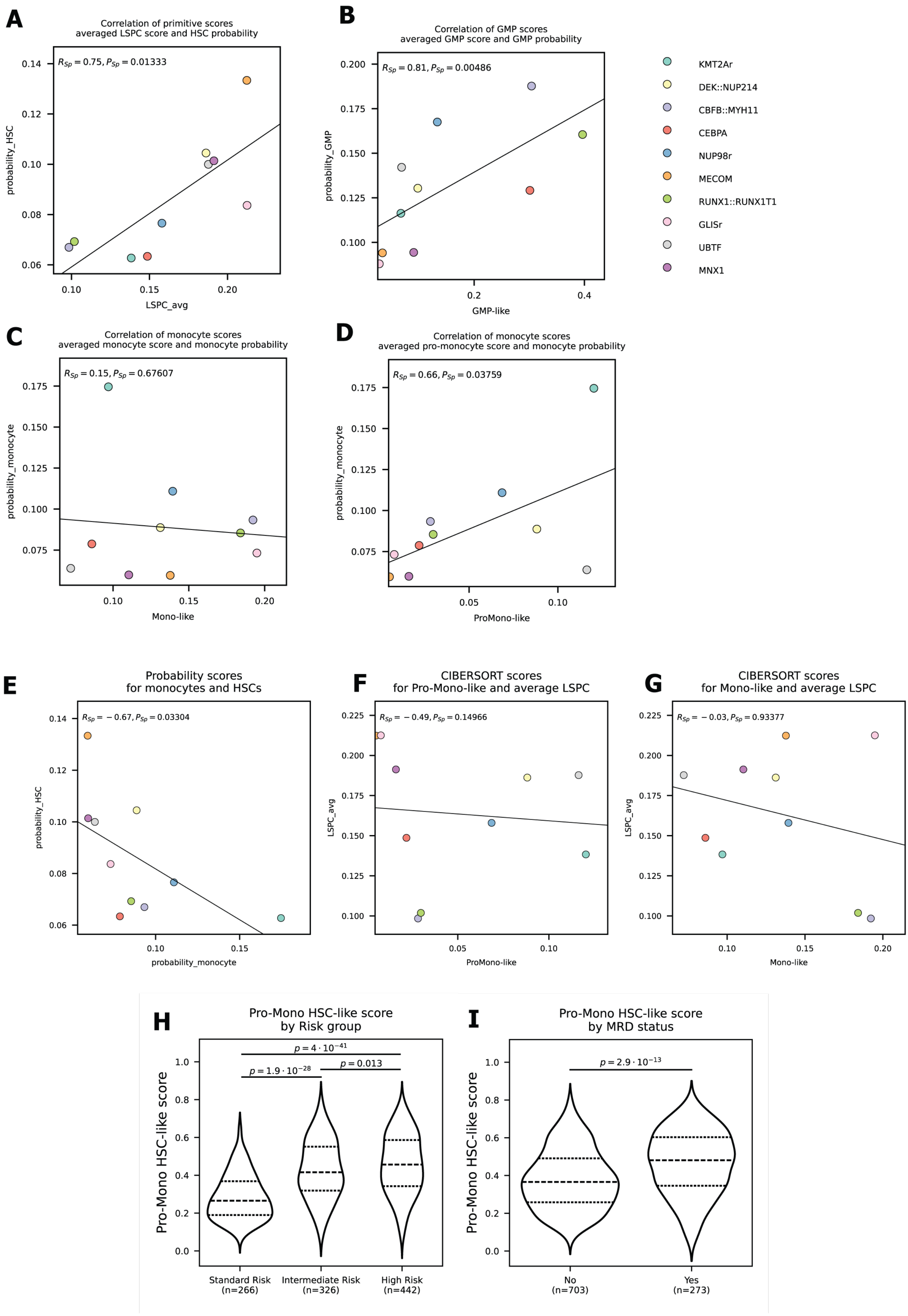
A
Correlation of primitive scores
averaged LSPC score and HSC probability
$R_{Sp} = 0.75, P_{Sp} = 0.01333$
probability_HSC
LSPC_avg
B
Correlation of GMP scores
averaged GMP score and GMP probability
$R_{Sp} = 0.81, P_{Sp} = 0.00486$
probability_GMP
GMP-like
KMT2Ar
DEK::NUP214
CBFB::MYH11
CEBPA
NUP98r
MECOM
RUNX1::RUNX1T1
GLISr
UBTF
MNX1
C
Correlation of monocyte scores
averaged monocyte score and monocyte probability
$R_{Sp} = 0.15, P_{Sp} = 0.67607$
probability_monocyte
Mono-like
D
Correlation of monocyte scores
averaged pro-monocyte score and monocyte probability
$R_{Sp} = 0.66, P_{Sp} = 0.03759$
probability_monocyte
ProMono-like
E
Probability scores
for monocytes and HSCs
$R_{Sp} = -0.67, P_{Sp} = 0.03304$
probability_HSC
probability_monocyte
F
CIBERSORT scores
for Pro-Mono-like and average LSPC
$R_{Sp} = -0.49, P_{Sp} = 0.14966$
LSPC_avg
ProMono-like
G
CIBERSORT scores
for Mono-like and average LSPC
$R_{Sp} = -0.03, P_{Sp} = 0.93377$
LSPC_avg
Mono-like
H
Pro-Mono HSC-like score
by Risk group
$p = 4 \cdot 10^{-41}$
$p = 1.9 \cdot 10^{-28}$
$p = 0.013$
Pro-Mono HSC-like score
Standard Risk (n=266)
Intermediate Risk (n=326)
High Risk (n=442)
I
Pro-Mono HSC-like score
by MRD status
$p = 2.9 \cdot 10^{-13}$
Pro-Mono HSC-like score
No (n=703)
Yes (n=273)

Figure S8: Validation analysis of cell type differentiation states. Related to Figure 5

A-D CIBERSORT scores from Umeda et al (x-axis) plotted against probabilities for cell differentiation states from this study (y-axis). Dots indicate median values per genetic subtype for *KMT2A*r (n=6 this study; n=236 Umeda et al.), *DEK::NUP214* (n=2 this study, n=17 Umeda et al.), *CBFB::MYH11* (n=6 this study, n=102 Umeda et al.), *CEBPA* (n=2 this study, n=63 Umeda et al.), *NUP98*r (n=5 this study, n=77 Umeda et al.), *MECOM* (n=1 this study, n=11 Umeda et al.), *RUNX1::RUNX1T1* (n=1 this study, n=141 Umeda et al.), *GLIS*r (n=1 this study, n=20 Umeda et al.), *UBTF* (n=2 this study, n=45 Umeda et al.), *MNX1* (n=1 this study, n=4 Umeda et al.) Black lines indicate linear model fit regression line. **A** Averaged LSPC CIBERSORT score and HSC probability **B** GMP-like CIBERSORT score and GMP probability **C** Mono-like CIBERSORT score and monocyte probability **D** Pro-Mono like CIBERSORT score and monocyte probability. **E-G** Dots and lines as in A-D. **E** HSC probability plotted against monocyte probability **F** averaged LSPC CIBERSORT score and Mono-like CIBERSORT score **G** averaged LSPC CIBERSORT score and Pro-Mono-like CIBERSORT score **H** Violin plot of Pro-Mono HSC score by AIEOP-BFM risk group **I** Violin plot of Pro-Mono HSC score by MRD status after induction 1. Widths are proportional to the number of samples within the corresponding value interval. Dashed lines in H and I separate the quartiles of the data.

Figure S9

A

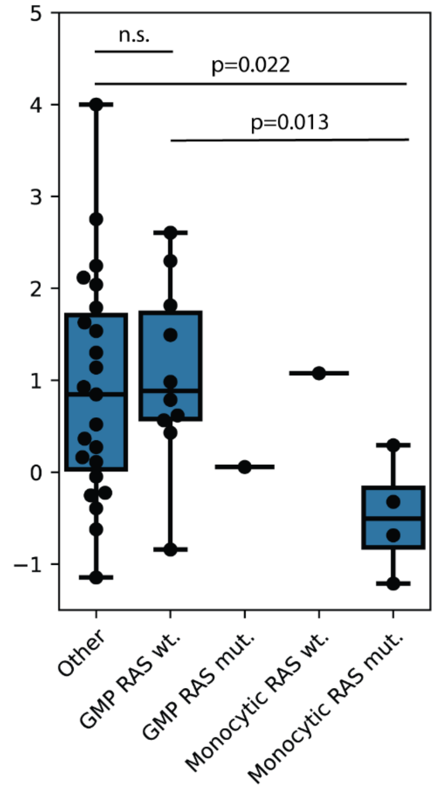

B

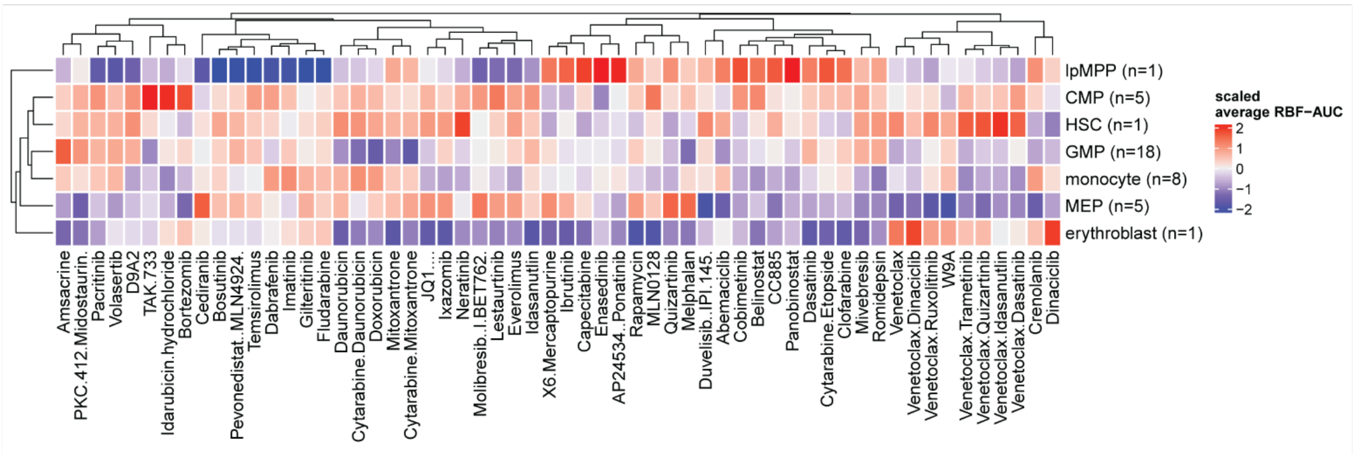

Figure S9: Associations of drug response and cellular differentiation states. Related to Figure 5

A Comparative analysis of Venetoclax response by RAS-mutation status and differentiation status for monocytic samples with mutated RAS (n=4), one monocytic sample with wild type RAS (n=1), one GMP-like sample with mutated RAS (n=1), GMP-like samples with wild type RAS (n=8) and samples that are neither GMP-like nor monocytic and have been termed Other (n=23). P-values were calculated using the Mann-Whitney-U Test. Dots indicate RBF-AUC values for individual samples. Boxes represent quartiles. Whiskers extend to points that are within 1.5 interquartile ranges. **B** Heatmap of scaled averaged RBF-AUC values for predicted differentiation states.

Supplementary Tables

Supplementary Table 1: Compound library overview. Related to Figure 1.

Compound	Compound MoA	Compound class	ConcPoint1 (micromolar)	ConcPoint2 (micromolar)	ConcPoint3 (micromolar)
Pentostatin	Adenosine deaminase inhibitor	Chemotherapeutic	1	0.1	0.01
Thiotepa	Alkylating agent	Chemotherapeutic	1	0.1	0.01
Palifosfamide	Alkylating agent	Chemotherapeutic	1	0.1	0.01
Busulfan	Alkylating agent	Chemotherapeutic	1	0.1	0.01
Melphalan	Alkylating agent	Chemotherapeutic	1	0.1	0.01
Aminopterin	Antimetabolite, Antifolate	Chemotherapeutic	1	0.1	0.01
Pralatrexate	Antimetabolite, Antifolate	Chemotherapeutic	0.7	0.07	0.007
Methotrexate	Antimetabolite, Antifolate	Chemotherapeutic	1	0.1	0.01
Doxorubicin	Antimetabolite, DNA intercalater	Chemotherapeutic	1	0.1	0.01
Idarubicin hydrochloride	Antimetabolite, DNA intercalater	Chemotherapeutic	1	0.1	0.01
Daunorubicin	Antimetabolite, DNA intercalater	Chemotherapeutic	1	0.1	0.01
Mitoxantrone	Antimetabolite, DNA intercalater, Topoisomerase 2 inhibitor	Chemotherapeutic	1	0.1	0.01
Pixantrone	Antimetabolite, DNA intercalater, Topoisomerase 2 inhibitor	Chemotherapeutic	1	0.1	0.01
Etoposide	Antimetabolite, DNA synthesis inhibitor	Chemotherapeutic	2	0.2	0.02
Hydroxyurea	Antimetabolite, DNA sythesis inhibitor, other effects	Chemotherapeutic	1	0.1	0.01
Amsacrine	Antimetabolite, DNA-intercalater	Chemotherapeutic	1	0.1	0.01
Thioguanine	Antimetabolite, Purines	Chemotherapeutic	1	0.1	0.01
6-Mercaptopurine	Antimetabolite, Purines	Chemotherapeutic	0.1	0.01	0.001
Clofarabine	Antimetabolite, Purines	Chemotherapeutic	1	0.1	0.01
Fludarabine	Antimetabolite, Purines	Chemotherapeutic	1	0.1	0.01
5-Fluorouracil	Antimetabolite, Pyrimidines	Chemotherapeutic	1	0.1	0.01
Cytarabine	Antimetabolite, Pyrimidines	Chemotherapeutic	1	0.1	0.01
Elacytarabine	Antimetabolite, Pyrimidines	Chemotherapeutic	1	0.1	0.01
Capecitabine	Antimetabolite, Pyrimidines	Chemotherapeutic	1	0.1	0.01
5-Azacytidine	Antimetabolite, Pyrimidines, Hypomethylating agent	Chemotherapeutic	3	0.3	0.03
Irinotecan	Antimetabolite, Topoisomerase 1 inhibitor	Chemotherapeutic	1	0.1	0.01
Venetoclax	BCL2-inhibitor	Cell death signaling (+/- Kinase inhibitor)	1	0.1	0.01
Molibresib (I-BET762)	BET inhibitor	Epigenetic	1	0.1	0.01
JQ1-(+)	BET inhibitor	Epigenetic	0.96	0.096	0.0096
Mivebresib	BET inhibitor	Epigenetic	1	0.1	0.01
Venetoclax+Dasatinib	Combination	Cell death signaling (+/- Kinase inhibitor)	0.5+0.5	0.05+0.05	0.005+0.005
Venetoclax+Ruxolitinib	Combination	Cell death signaling (+/- Kinase inhibitor)	0.5+0.5	0.05+0.05	0.005+0.005
Venetoclax+Idasanutlin	Combination	Cell death signaling (+/- Kinase inhibitor)	0.5+0.5	0.05+0.05	0.005+0.005
Venetoclax+Dinaciclib	Combination	Cell death signaling (+/- Kinase inhibitor)	0.5+0.5	0.05+0.05	0.005+0.005
Venetoclax+Trametinib	Combination	Cell death signaling (+/- Kinase inhibitor)	0.5+0.5	0.05+0.05	0.005+0.005

Venetoclax+Quizartinib	Combination	Cell death signaling (+/- Kinase inhibitor)	0.5+0.5	0.05+0.05	0.005+0.005
Cytarabine+Daunorubicin	Combination, Antimetabolite	Chemotherapeutic	0.5+0.5	0.05+0.05	0.005+0.005
Cytarabine+Etopside	Combination, Antimetabolite	Chemotherapeutic	0.5+0.5	0.05+0.05	0.005+0.005
Cytarabine+Mitoxantrone	Combination, Antimetabolite	Chemotherapeutic	0.5+0.5	0.05+0.05	0.005+0.005
Pinemetostat (EPZ-5676)	Epigenetic drug	Epigenetic	1	0.1	0.01
Romidepsin	Epigenetic drug, HDAC inhibitor	Epigenetic	2	0.2	0.02
Belinostat	Epigenetic drug, HDAC inhibitor	Epigenetic	1	0.1	0.01
Panobinostat	Epigenetic drug, HDAC inhibitor	Epigenetic	5	0.5	0.05
ORY1001	Epigenetic drug, Histone demethylase inhibitor	Epigenetic	1	0.1	0.01
Tazemetostat (EPZ-6438)	Epigenetic drug, Histone methyltransferase inhibitor	Epigenetic	1	0.1	0.01
Guadecitabine	Epigenetic drug, hypomethylating agent	Epigenetic	1	0.1	0.01
Decitabine	Epigenetic drug, hypomethylating agent	Epigenetic	1	0.1	0.01
GSK126	Epigenetic drug, Methyltransferase inhibitor	Epigenetic	1	0.1	0.01
Prednisolone	glucocorticoid	Other	1	0.1	0.01
Vismodegib	Hedgehog pathway inhibitor	Kinase inhibitor	2	0.2	0.02
Glasdegib	Hedgehog pathway inhibitor	Kinase inhibitor	1	0.1	0.01
Ivosidenib	IDH inhibitor	Metabolic drug	1	0.1	0.01
Enasedinib	IDH inhibitor	Metabolic drug	1	0.1	0.01
Ceritinib	Kinase inhibitor, ALK	Kinase inhibitor	1	0.1	0.01
Crizotinib	Kinase inhibitor, ALK	Kinase inhibitor	2	0.2	0.02
Alisertib	Kinase inhibitor, Aurora	Kinase inhibitor	1	0.1	0.01
Bafetinib	Kinase inhibitor, BCR-ABL	Kinase inhibitor	1	0.1	0.01
Imatinib	Kinase inhibitor, BCR-ABL	Kinase inhibitor	1	0.1	0.01
AP24534 Ponatinib	Kinase inhibitor, BCR-ABL	Kinase inhibitor	1	0.1	0.01
Dasatinib	Kinase inhibitor, BCR-ABL	Kinase inhibitor	1	0.1	0.01
Nilotinib	Kinase inhibitor, BCR-ABL	Kinase inhibitor	2	0.2	0.02
Bosutinib	Kinase inhibitor, BCR-ABL	Kinase inhibitor	1	0.1	0.01
Vemurafenib	Kinase inhibitor, BRAF	Kinase inhibitor	2	0.2	0.02
Dabrafenib	Kinase inhibitor, BRAF	Kinase inhibitor	1	0.1	0.01
Ibrutinib	Kinase inhibitor, BTK	Kinase inhibitor	1	0.1	0.01
Dinaciclib	Kinase inhibitor, CDK	Kinase inhibitor	1	0.1	0.01
Palbociclib	Kinase inhibitor, CDK	Kinase inhibitor	1	0.1	0.01
Abemaciclib	Kinase inhibitor, CDK	Kinase inhibitor	1	0.1	0.01
Neratinib	Kinase inhibitor, EGFR	RTK inhibitor	1	0.1	0.01
Erlotinib	Kinase inhibitor, EGFR	RTK inhibitor	2	0.2	0.02
Gefitinib	Kinase inhibitor, EGFR	RTK inhibitor	1	0.1	0.01
Gilteritinib	Kinase inhibitor, FLT3	RTK inhibitor	0.1	0.01	0.001
Quizartinib	Kinase inhibitor, FLT3	RTK inhibitor	1	0.1	0.01
Crenolanib	Kinase inhibitor, FLT3	RTK inhibitor	1	0.1	0.01
Lestaurtinib	Kinase inhibitor, FLT3	RTK inhibitor	1	0.1	0.01
Ruxolitinib	Kinase inhibitor, JAK	Kinase inhibitor	1	0.1	0.01
Tofacitinib	Kinase inhibitor, JAK	Kinase inhibitor	1	0.1	0.01
Pacritinib	Kinase inhibitor, JAK	Kinase inhibitor	1	0.1	0.01
Trametinib	Kinase inhibitor, MEK	Kinase inhibitor	1	0.1	0.01
Cobimetinib	Kinase inhibitor, MEK	Kinase inhibitor	1	0.1	0.01

TAK-733	Kinase inhibitor, MEK	Kinase inhibitor	1	0.1	0.01
Idelalisib	Kinase inhibitor, PI3K	Kinase inhibitor	1	0.1	0.01
Duvelisib (IPI-145)	Kinase inhibitor, PI3K	Kinase inhibitor	1	0.1	0.01
Volasertib	Kinase inhibitor, Plk	Kinase inhibitor	1	0.1	0.01
Sunitinib	Kinase inhibitor, RTK	RTK inhibitor	1	0.1	0.01
Nintedanib	Kinase inhibitor, RTK	RTK inhibitor	1	0.1	0.01
Cabozantinib	Kinase inhibitor, unspecific	RTK inhibitor	2	0.2	0.02
Regorafenib	Kinase inhibitor, unspecific	Kinase inhibitor	2	0.2	0.02
PKC-412(Midostaurin)	Kinase inhibitor, unspecific	RTK inhibitor	1	0.1	0.01
Sorafenib	Kinase inhibitor, unspecific	Kinase inhibitor	1	0.1	0.01
Vandetanib	Kinase inhibitor, VEGFR	RTK inhibitor	2	0.2	0.02
Semaxanib	Kinase inhibitor, VEGFR	RTK inhibitor	1	0.1	0.01
Pazopanib	Kinase inhibitor, VEGFR	RTK inhibitor	0.443	0.0443	0.00443
Cediranib	Kinase inhibitor, VEGFR	RTK inhibitor	1	0.1	0.01
Idasanutlin	MDM2 inhibitor	Cell death signaling (+/- Kinase inhibitor)	1	0.1	0.01
Temsirolimus	mTOR inhibitor	Metabolic drug	1	0.1	0.01
Everolimus	mTOR inhibitor	Metabolic drug	1	0.1	0.01
Rapamycin	mTOR inhibitor	Metabolic drug	1	0.1	0.01
MLN0128	mTOR inhibitor	Metabolic drug	1	0.1	0.01
MLN2480	mTOR inhibitor	Metabolic drug	1	0.1	0.01
Pevonedistat (MLN4924)	NEDD inhibitor	Metabolic drug	1	0.1	0.01
Veliparib	PARP inhibitor	Epigenetic	1	0.1	0.01
Olaparib	PARP inhibitor	Epigenetic	1	0.1	0.01
Carfilzomib	Proteasome inihibitor	Metabolic drug	0.000117	0.0000117	0.00000117
Ixazomib	Proteasome inihibitor	Metabolic drug	1	0.1	0.01
Bortezomib	Proteasome inihibitor	Metabolic drug	1	0.1	0.01
Bexarotene	Retinoid	Other	2	0.2	0.02
Alitretinoin	Retinoid receptor activator	Other	2	0.2	0.02
CC885	SLC9A1 degradation toxicitiy control	Other	1	0.1	0.01
D9A2	SLC9A1 degrader	Metabolic drug	1	0.1	0.01
W9A	SLC9A1 inhibitor	Metabolic drug	1	0.1	0.01
Rosuvastatin	Statin	Metabolic drug	1	0.1	0.01
Lovastatin	Statin	Metabolic drug	1	0.1	0.01
Telaglenastat (CB-839)	TCA cycle inhibitor, targets Glutaminase	Metabolic drug	1	0.1	0.01
Devimistat	TCA cycle inhibitor, targets Pyruvate Dehydrogenase	Metabolic drug	1	0.1	0.01

Supplementary Table 2: Extended patient information. Related to Figure 2.

Extended information on patient samples in the cohort. (WES) next do detected mutations in the Additional alterations column indicates that the alterations have been identified via whole-exome sequencing. Abbreviations are the following: NK: Normal Karyotype; WES: Whole exome sequencing; ND: not detected/not assessed; Dx: Diagnosis; SCT: stem cell transplantation; FUP: follow up; MRD: Measurable residual disease; SR: Standard risk; IR: Intermediate risk; HR: High risk; pos: positive; neg: negative

Sample ID	Sex	Age at Dx (years)	FAB	Cytogenetics	Additional alterations	FCM-MRD (d21 or d28)	PCR MRD d28	MRD vote Ind1	FCM-MRD d56	PCR MRD d56	MRD vote Ind2	Risk BFM 2019	RISK BFM 2019 post Ind1	Time to death (days)	Time to relapse (days)	Time to SCT (days)	Time to FUP (days)	Treatment _protocol	Umeda 2024 category
AML74	male	17,8	M4	NK	FLT3-ITD	7.50 %	ND	pos	ambiguous	nd	neg	IR	HR	745	303	no SCT	745	AML-BFM 04 mod	UBTF
AML20	female	3,8	M1	NK	FLT3-ITD NPM1	0.53 %	ND	pos	ambiguous	nd	neg	IR	HR	alive	no relapse	no SCT	603	AML-BFM 2012	NPM1
AML82	female	16,5	M1	NK		neg	ND	neg	neg	neg	neg	IR	IR	alive	798	no SCT	1358	AML-BFM 2013	CBFB-GDXY
AML64	male	5,7	M1	NK		neg	ND	neg	neg	nd	neg	IR	IR	alive	no relapse	no SCT	1868	AML-BFM 2004	unclassified
AML31	male	11,8	M7	BCR::ABL1		ND	ND	ND	nd	nd	ND	HR	HR	alive	no relapse	139	2230	AML04 Interim	BCR-ABL1
AML93	female	7,4	M0	ETV6::MNX1		ND	ND	ND	nd	nd	ND	HR	HR	668	480	100	668	AML04 Interim	MNX1
AML37	male	1,4	M5a	KMT2A::MLLT1		neg	<0,01 %	neg	neg	neg	neg	IR	IR	alive	no relapse	no SCT	1091	AML-BFM 2012	KMT2Ar
AML06	male	1,4	M7	NUP98::KDM5A		10.60%	2%	pos	0.104%	0.09%	pos	HR	HR	254	154	no SCT	254	AML-BFM 2012	NUP98r AMKL
AML57	male	18,3	M2/MDS !	trisomy 8	NRAS, WT1	20%	ND	pos	70%	nd	pos	IR	HR	alive	no relapse	67	246	AML-BFM 2004	UBTF
AML84	female	1,4	M7	complex		neg	ND	neg	neg	nd	neg	HR	HR	alive	no relapse	no SCT	607	AML-BFM 2012	unclassified
AML13	male	17,7	M2	KMT2A::MLLT1	FLT3-ITD, DNMT3A, RAD21	0.09 %	0.70%	pos	neg	<0,01 %	neg	IR	HR	alive	326	625	938	AML-BFM 2012	KMT2Ar
AML39	female	0,1	M4	DEK::NUP214	FLT3-ITD	3.10 %	ND	pos	2.20%	nd	pos	HR	HR	alive	no relapse	134	322	AML-BFM 2012	DEK-NUP214
AML44	female	12,8	M7	NUP98::KDM5A	KIT	0.38 %	ND	pos	neg	nd	neg	HR	HR	alive	no relapse	no SCT	911	AML-BFM 2012	NUP98r AMKL

AML66	female	8,9	M4Eo	CBFB::MYH11		neg	ND	neg	neg	nd	neg	SR	SR	alive	no relapse	no SCT	1207	AML-BFM 2012	CBFB-MYH11
AML47	male	17,4	M4Eo	CBFB::MYH11	NRAS, KIT	ambiguous	ND	neg	ambiguous	nd	neg	SR	SR	alive	no relapse	no SCT	237	AML-BFM 2012	CBFB-MYH11
AML58	male	4,4	M2	NK	CEBPAdm	neg	nd	neg	neg	nd	neg	SR	SR	alive	no relapse	no SCT	555	AML-BFM 2012	CEBPA
AML63	male	4,6	M5a	NUP98::NSD1	FLT3-ITD, GATA2	ambiguous	ND	neg	ambiguous	nd	neg	HR	HR	alive	973	973	1635	AML-BFM 04	NUP98r
AML62	female	14,2	M4	DEK::NUP214	FLT3-ITD	nd	ND	ND	neg	nd	neg	HR	HR	alive	no relapse	134	1362	AML-BFM 2004	DEK-NUP214
AML59	male	7,6	M4Eo	CBFB::MYH11		0.10 %	ND	pos	neg	nd	neg	SR	IR	alive	no relapse	no SCT	2313	AML04 Interim	CBFB-MYH11
AML77	male	8,5	M4	trisomy 8	GATA2	1.50 %	ND	pos	0.36%	nd	pos	IR	HR	66	no relapse	63	66	AML-BFM 04	unclassified
AML26	female	6,1	M4Eo	CBFB::MYH11		ND	ND	ND	nd	nd	ND	SR	SR	alive	no relapse	no SCT	2457	AML04 Interim	CBFB-MYH11
AML88	male	5,1	M5	monosomy 7	NRAS	neg	ND	neg	neg	nd	neg	HR	HR	alive	no relapse	32	2190	AML-BFM 2004	unclassified
AML95	male	15,4	M4	inv(3)(q21q26) RPN1/MECOM		ND	ND	ND	38%	nd	pos	HR	HR	319	190	93	319	AML-BFM 04	MECOM
AML54	male	0,1	M5	KMT2A::MLLT3		ambigous 0,17 %	<0,01 %	neg	0.01%	0.03%	neg	IR	IR	342	237	175	342	AML-BFM 2012	KMT2Ar
AML07	female	15,3	M5b	KMT2A::MLLT3		neg	0.20%	pos	neg	<0,01 %	neg	IR	HR	alive	no relapse	no SCT	979	AML-BFM 2012	KMT2Ar
AML60	male	9,8	M4Eo	CBFB::MYH11		neg	nd	neg	neg	nd	neg	SR	SR	alive	1069	1175	1667	AML04 Interim	CBFB-MYH11
AML98	female	1,4	M4Eo	CBFB::MYH11		ND	ND	ND	neg	nd	neg	SR	SR	alive	no relapse	no SCT	2343	AML04 Interim	CBFB-MYH11
AML89	female	4,5	M2	RUNX1::RUNX1T1		ND	ND	ND	nd	nd	ND	SR	SR	alive	no relapse	no SCT	3499	AML04 Interim	RUNX1-RUNX1T1
AML76	male	16,8	M5	KMT2A::MLLT3		neg	neg	neg	neg	neg	neg	IR	IR	alive	no relapse	no SCT	1583	AML-BFM 2004	KMT2Ar
AML87	male	8,9	M7	NK	ETV6, JAK3, WT1	0.54 %	0.30%	pos	neg	neg	neg	IR	HR	949	413	no SCT	949	AML-BFM 2012	unclassified
AML50	male	3,3	M7	CBFA2T3::GLIS2	GATA2	3.07 %	4%	pos	neg	neg	neg	HR	HR	alive	no relapse	no SCT	1368	AML-BFM 2012	GLISr
AML52	female	11	M5	KMT2A::MLLT10		neg	<0,01 %	neg	neg	neg	neg	HR	HR	alive	no relapse	153	956	AML-BFM 2012	KMT2Ar

AML65	female	11,3	M5	KMT2A::MLLT3	NRAS	neg	neg	neg	neg	neg	neg	IR	IR	alive	no relapse	no SCT	410	AML-BFM 2012	KMT2Ar
AML23	female	7,2	M5a	KMT2A::MLLT1	GATA2, KRAS, NRAS	0.36 %	0.20%	pos	neg	neg	neg	IR	HR	alive	293	no SCT	1441	AML-BFM 2012	KMT2Ar
AML67	female	1,1	M5a	KMT2A::MLLT3		neg	<10-4	neg	neg	neg	neg	IR	IR	alive	no relapse	no SCT	1730	AML-BFM 2004	KMT2Ar
AML28	female	16,4	M1	NK	FLT3, NRAS, WT1 (WES)	ND	ND	ND	ND	ND	ND	IR	IR	472	314	394	472	AML-BFM 2004	unclassified
AML24	male	10,3	M4	trisomy 8	FLT3-ITD, UBTF, (WES)	ND	ND	ND	ND	ND	ND	IR	IR	alive	421	519	2191	(AML-BFM 04) different therapy	UBTF
AML43	male	17,8	M2	NK	CEBPAdm (WES)	ND	ND	ND	ND	ND	ND	SR	SR	alive	no relapse	no SCT	1735	AML-BFM 2004	CEBPA
AML04	male	6,7	M1	NK	FLT3-ITD	ND	ND	ND	ND	ND	ND	IR	IR	534	277	357	534	AML-BFM 2004	unclassified
AML81	female	12,6	M4	other	FLT3 (WES)	ND	ND	ND	ND	ND	ND	IR	IR	alive	no relapse	no SCT	2961	AML-BFM 2004	unclassified
AML17	female	16,9	M4	NUP98::NSD1	FLT3	ambigous	1%	pos	ambiguous	0.10%	pos	HR	HR	alive	no relapse	137	137	AML-BFM 2012	NUP98r
AML12	male	10,8	M2	NUP98::NSD1	NRAS	32.71%	50%	pos	neg	0.04%	neg	HR	HR	alive	463	159	463	AML-BFM 2012	NUP98r
AML29	female	3,9	M2	NUP98::NSD1		ND	ND	ND	ND	ND	ND	HR	HR	alive	397	146	4190	(AML-BFM 04) different therapy	NUP98r
AML55	male	7,6	M5	NUP98::KDM5A		neg	<0,01 %	neg	neg	neg	neg	HR	HR	alive	no relapse	181	543	AML-BFM recommendations 2019	NUP98r
AML34	male	1,7	M6/M7	NUP98::KDM5A		0.21 %	0.40%	pos	0.34%	0.30%	pos	HR	HR	alive	no relapse	no SCT	258	AML-BFM recommendations 2019	NUP98r AMKL
