# Supplementary material for: Image-based drug screening combined with molecular profiling identifies signatures and drivers of therapy resistance in pediatric AML
Source: Cell Rep Med. 2025 Aug 20;6(9):102304. doi: 10.1016/j.xcrm.2025.102304 (PMC12490222; doi:10.1016/j.xcrm.2025.102304)
Supplement: Document S2. Article plus supplemental information [file mmc2.pdf]

Cell Reports
Medicine

Article

Image-based drug screening combined with molecular profiling identifies signatures and drivers of therapy resistance in pediatric AML

Graphical abstract

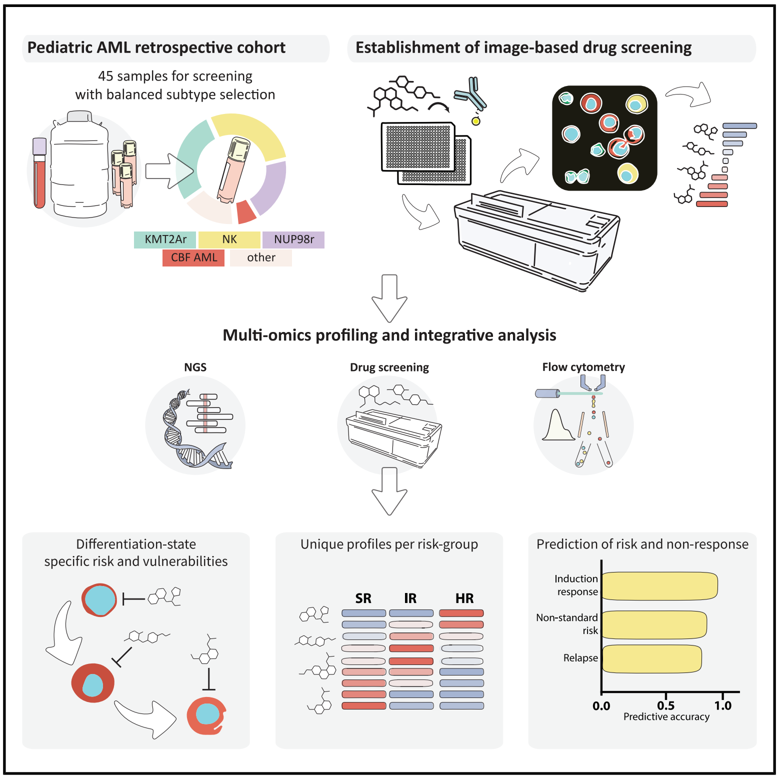

Authors

Ben Haladik,
Margarita Maurer-Granofszky,
Peter Zoescher, ..., Michael N. Dworzak,
Giulio Superti-Furga, Kaan Boztug

Correspondence

kaan.boztug@ccri.at

In brief

Haladik et al. establish high-content imaging and deep-learning-based drug response profiling for pediatric acute myeloid leukemia. Combined with multi-omics profiling, they identify treatment options for epigenetically defined chemotherapy-resistant cell states and demonstrate the predictivity of drug response profiles for patient risk and response.

Highlights

- Establishment of image-based drug response profiling for pediatric AML
- Identification of pediatric AML differentiation states with ATAC-seq
- Pediatric AML differentiation states exhibit distinct drug response and risk profiles
- Pediatric AML drug response profiling at diagnosis may predict therapy response

Haladik et al., 2025, Cell Reports Medicine 6, 102304
September 16, 2025 © 2025 The Authors. Published by Elsevier Inc.
https://doi.org/10.1016/j.xcrm.2025.102304

CellPress

Cell Reports Medicine

CellPress
OPEN ACCESS

Article

Image-based drug screening combined with molecular profiling identifies signatures and drivers of therapy resistance in pediatric AML

Ben Haladik,[1,2] Margarita Maurer-Granofszky,[1] Peter Zoescher,[1] Raul Jimenez-Heredia,[1,3] Alexandra Frohne,[1] Anna Segarra-Roca,[1] Chloe Casey,[1] Felix Kartnig,[2,4] Sarah Giuliani,[1] Christina Rashkova,[1,3] Peter Repiscak,[1] Michael N. Dworzak,[1,3,6,8] Giulio Superti-Furga,[2,5,8] and Kaan Boztug[1,2,3,6,7,8,9,*]

[1]St. Anna Children's Cancer Research Institute, Vienna, Austria
[2]CeMM Research Center for Molecular Medicine of the Austrian Academy of Sciences, Vienna, Austria
[3]Medical University of Vienna, Department of Pediatrics and Adolescent Medicine, Vienna, Austria
[4]Medical University of Vienna, Department of Internal Medicine III, Division of Rheumatology, Vienna, Austria
[5]Medical University of Vienna, Center for Physiology and Pharmacology, Vienna, Austria
[6]St. Anna Children's Hospital, Vienna, Austria
[7]Clinic for Pediatric Immunology and Rheumatology, Center for Pediatrics and Adolescent Medicine, University Hospital Bonn, Bonn, Germany
[8]These authors contributed equally
[9]Lead contact
*Correspondence: kaan.boztug@ccri.at
https://doi.org/10.1016/j.xcrm.2025.102304

SUMMARY

Despite recent advances in the understanding of the genomic landscape of pediatric acute myeloid leukemia (pedAML), targeted treatments are only available for selected genomic alterations, and the functional link between genotype and outcome remains partially elusive. Functional precision medicine approaches to investigate treatment resistance and patient risk have not been applied systematically for pedAML. Here, we describe an advanced functional screening platform combining high-content imaging and deep learning-based phenotyping. In 45 patients with pedAML, we identify BCL2 and FLT3 inhibitors and standard chemotherapy as major drivers of the chemosensitivity landscape, reveal substantial differential sensitivities between risk groups, and may effectively predict individual measurable residual disease and patient risk. Integration with genomic and epigenomic data uncovers a chemotherapy-resistant primitive state vulnerable to combined BCL2 and MDM2 inhibition and HDAC inhibition. Overall, we identify early signatures of therapy resistance across genetic subgroups and prioritize targeted treatments for these functionally and epigenetically defined patient subsets.

INTRODUCTION

Pediatric acute myeloid leukemia (pedAML) is a rare hematological malignancy with poorer outcome than its lymphoblastic counterpart in children and adolescents and fundamentally different biology than in adult patients.[1,2] These differences are characterized by the disproportionally higher prevalence of structural aberrations in pedAML such as, for instance, *KMT2A* or *NUP98* rearrangements. Frequencies of non-structural mutations also differ markedly: *NRAS*, *KRAS*, *KIT*, and *WT1*, for example, are more commonly affected in younger patients, whereas variants in *DNMT3A*, *TP53*, and *NPM1* are more frequent in older patients.[1] Through rigorous optimization of treatment protocols over the past decades, 5-year overall survival (OS) rates have dramatically increased in most countries of the world,[2] and recent clinical trials now report 5-year OS between 60% and 80% in Western European countries[3–5] and the US.[6] Large-scale efforts in recent years have furthered our understanding of the genetic determinants of patient risk and poor outcome in this disease,[1,7] revealed novel genomic subtypes,[8] and elucidated the trajectories of cellular composition hierarchies between diagnosis and relapse.[9] Furthermore, several studies in recent years have addressed poor outcome in selected genetically defined subgroups by identifying targetable disease mechanisms and novel therapeutic agents with menin inhibitors being particularly promising in *KMT2A*- and *NUP98*-rearranged leukemias.[10,11]

A systematic understanding of the functional basis of treatment resistance and poor response in pedAML has remained elusive, and individual contributions of subclonal evolution, pharmacogenomics, and germline variants have increasingly been recognized. Deep sequencing of genomic DNA in matched samples of diagnosis and non-response or relapse revealed patient-specific expansions of subclones with more prevalent genetic variants,[12] which may at least partially drive therapy resistance.[7,13] Pharmacogenomic efforts over the past decades

This is an open access article under the CC BY license (http://creativecommons.org/licenses/by/4.0/).

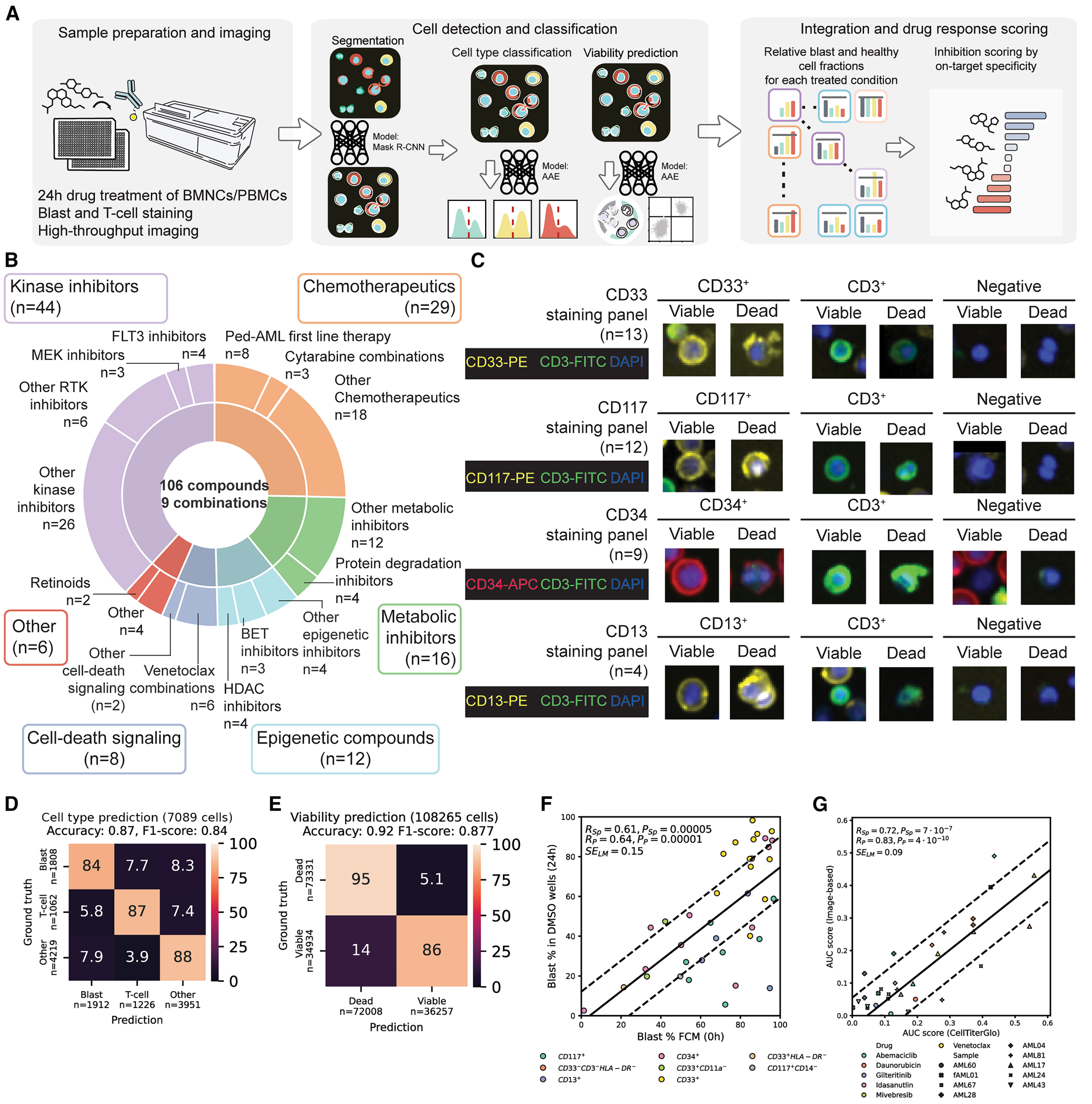

Figure 1. Establishing an image-based drug sensitivity profiling platform for pedAML

(A) Workflow. Mononuclear cells from either peripheral blood (PBMCs) or bone marrow (BMNCS) were incubated on plates with pre-printed compounds. After 24 h of incubation, these cells were stained with blast- and T cell-specific markers and imaged. A custom pipeline then used deep learning models to classify cells by their marker expression profile and viability.

(B) Compound library overview. Pie-chart of relative abundancies per drug class in our custom compound library of 106 compounds and 9 combinations. RTK, receptor tyrosine kinase.

(C) Example images for the four most common staining panels in our pipeline covering $n = 38$ samples. Each row indicates a staining panel. Images are contrast adjusted single-cell images for the three channels indicated for each row.

(D) Normalized confusion matrix for cell type prediction for $n = 7{,}089$ cells in the testing dataset.

(E) Normalized confusion matrix for viability prediction for $n = 108{,}265$ cells in the testing dataset.

(F) Scatterplot of relative blast abundancies among viable cells for flow cytometry (x axis) and image-based profiling (y axis) for $n = 38$ samples where flow cytometry-based blast fractions were available. The black line indicates the linear model fit for flow cytometry and image-based values. Dashed lines indicate the

(legend continued on next page)

have identified several variants that affect drug metabolism, and seminal work in acute lymphoblastic leukemia has uncovered genetic determinants of anti-cancer activity or increased off-target toxicities for commonly used anti-leukemic agents.[14,15] Similarly, though not investigated at the same level of detail to date, more recent work in pedAML indicated that patients with SNPs that affect cellular accumulation of the active metabolite of cytarabine, cytarabine triphosphate, have inferior outcomes.[16,17] Furthermore, there are several germline variants that predispose to pedAML. For example, patients with Fanconi anemia, GATA2 deficiency, or Down syndrome all have a tendency to develop pedAML and require distinct treatment approaches.[18–20] In addition to these genetic drivers of predisposition and patient risk, several studies demonstrated that measurable residual disease (MRD) after induction—a surrogate for *in vivo* treatment response—provides prognostic value over the years and even well into relapse,[8,21–24] indicating that characteristics beyond the broadly recognized risk-stratifying mutations and genomic aberrations are further determinants of patient outcome. Thus, collectively, the identification of additive risk-conferring functional properties may allow to better predict outcome and optimally match patients to clinical trials.

One particularly promising strategy to address the aforementioned challenges in predicting individual patient response to therapy is to employ so-termed functional precision medicine approaches, which aim to identify efficacious agents in a personalized fashion by directly measuring the effects of candidate drugs in primary patient material.[25] Recent work in this field has shown promise for improving outcome in late-stage hematological malignancies in adults,[26,27] in several high-risk pediatric populations,[28–30] and therefore highlighted the potential use of functional screenings as a stratification and discovery tool.[31,32] While these studies demonstrated promising results for biological discovery and potential improvements in outcome, challenges such as the interpretation of screening hits, the applicability of functional screening alongside established treatment concepts, and the identification of meaningful molecular correlates of *ex vivo* drug responses to derive robust insights from contextualizing compound screening data with molecular profiling data still remain largely unaddressed.

We here set out to systematically characterize patients with pedAML by advancing our previously established functional drug sensitivity profiling platform and integrating the resulting profiles with comprehensive genomic, transcriptomic, and epigenomic data. Through the comprehensive characterization of 45 patients, retrospectively sampled at diagnosis, we identified clearly distinguishable chemosensitivity profiles between risk groups as defined by the Associazione Italiana di Ematologia e Oncologia Pediatrica - Berlin Frankfurt Münster (AIEOP-BFM) AML consortium (EUCT: 2022-500783-35-00), revealed intriguing associations of drug response with cellular hierarchy compositions, and demonstrated the predictivity of *ex vivo* profiles for key clinical parameters.

RESULTS

Establishment of an image-based high-throughput compound screening platform for pedAML

To dissect the integrated chemosensitivity landscape of pedAML, we first set out to tailor our established image-based functional screening platform—previously termed Pharmacoscopy[26,27]—to some of the specific challenges in pedAML, such as heterogeneity in material amounts and blast fractions at diagnosis, the lack of universal blast-specific markers across cases and entities, and the challenges with *ex vivo* testing of myeloid blasts as described elsewhere.[33]

Hence, we established an image-based drug screening platform to quantify on-target drug activity against malignant blasts in primary mononuclear cells from blood or bone marrow. We treated cells with a custom, pedAML-specific compound library of 115 compounds and subsequently stained for markers as identified via our well-established flow cytometry based blast identification,[34] thereby explicitly accounting for the patient- and blast-specific immunophenotype (Figures 1A and 1B; Table S1). Our custom image analysis pipeline segmented cells with the Mask-R-CNN model[35] and classified them into viable and non-viable cells as well as marker-positive or marker-negative cells with adversarial autoencoders[36] (Figures 1A and 1C). Our cell type prediction accurately assessed cell marker positivity with an accuracy of 92% (Figure S1A) and roughly uniform predictive power across markers (Figure S1B), leading to an overall accuracy of 87% for the distinction between blasts, T cells, and other cells (Figure 1D). Our viability prediction model predicted cell viability as measured by staining for cells with fractured membranes with 92% accuracy (Figure 1E, STAR Methods). These approaches led to good agreement with orthogonal methods: leukemic blast fractions in untreated negative control wells correlated significantly with blast fractions as determined via flow cytometry (Pearson $R = 0.64$, $p = 0.00001$, Spearman $R = 0.61$, $p = 0.00005$, Figure 1F), which is similar to the performance described in a previous study.[26,37–41] We further assessed the robustness of our predictive viability model by performing image-based drug sensitivity profiling and measurements of metabolic activity via CellTiter-Glo in parallel and also found significant and high correlations of these orthogonal viability measurements across the 8 samples and 6 compounds we measured (Pearson $R = 0.83$, $p = 4{\cdot}10^{-10}$, Spearman R: 0.72, $p = 7{\cdot}10^{-7}$; Figure 1G). Notably, these correlations largely remained robust to 2- and 4-fold decreases in cell numbers (Figure S1C). Additional measurements of replicate samples further confirmed the robustness of this approach with Pearson correlations between replicates consistently above 0.8 across readouts—similar to other studies[38] (Figure S1D). Thus, our high-throughput imaging approach and custom image analysis pipeline allowed us to faithfully distinguish between blasts and healthy cells and to calculate on-target drug responses enabling targeted drug sensitivity profiling in pedAML.

standard error. Abbreviations: R_{Sp}, Spearman rank correlation coefficient; P_{Sp}, *p* value of Spearman rank correlation; R_P, Pearson correlation coefficient; P_P, *p* value of Pearson correlation; SE_{Lm}, normalized standard error of the linear model.
(G) Scatterplot of inhibition scores for CTG (*x* axis) and image-based profiling (*y* axis) for $n = 9$ samples and the 6 drugs tested. Lines and abbreviations are as in (F). Compounds were tested at four concentrations in technical quadruplicates per concentration (STAR Methods).

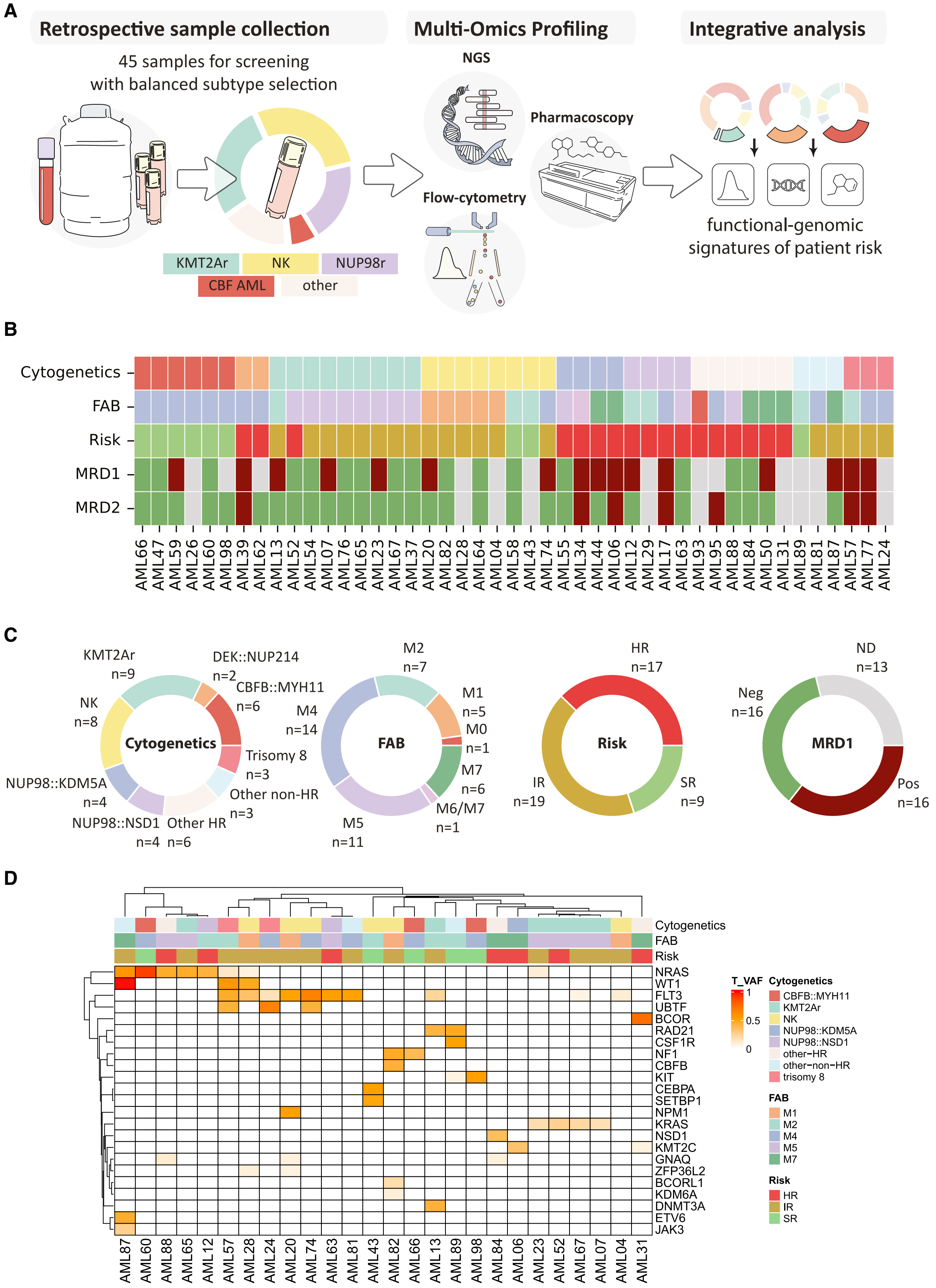

(legend on next page)

CellPress
OPEN ACCESS

Molecular and clinical cohort characteristics

We hypothesized that systematic application of this platform integrated with comprehensive molecular characterization may enable the identification of actionable early predictors of patient risk and non-response. Therefore, we combined our image-based chemosensitivity testing approach with detailed molecular profiling via whole-exome sequencing (WES), RNA sequencing, and assay for transposase accessible chromatin using sequencing (ATAC-seq) (Figure 2A). We applied this strategy to 45 fresh-frozen samples taken at diagnosis, thereby generating a comprehensive dataset comprising patients sampled at equivalent time points and treated according to highly similar treatment algorithms with a common cytarabine- and anthracycline-based chemotherapy backbone as established by the AIEOP-BFM study group.[3] We used five additional fresh pedAML samples for assay optimization and benchmarking. Initially, we selected available samples to cover all major cytogenetic subgroups and achieve enrichment for intermediate-risk (IR) and high-risk (HR) cases and subsequently categorized them based on diagnostic reports and our WES data. Samples with a normal karyotype, *CBFB::MYH11* fusions, and *KMT2A* rearrangements made up the most frequent subtypes in our cohort (Figures 2B and 2C). In addition to this grouping by recurring cytogenetic alterations, we assigned samples with cytogenetic alterations that occurred only once or had an unknown genetic subtype in our cohort into the groups other-non-HR (1× *RUNX1::RUNX1T1*, 2× unknown genetic alteration) and other-HR (*ETV6::MNX1*, *RPN1::MECOM*, *BCR::ABL1*, *CBFA2T3::GLIS2*, monosomy 7, complex karyotype). We further classified samples by risk according to the criteria established in the AIEOP-BFM-AML 2020 trial (EUCT: 2022-500783-35-00), based on genomic findings and response to induction therapy. Additionally, we annotated our cohort with data on MRD after induction at measurement time point 1 at day 21 or day 28 after first induction and time point 2 at day 56 after second induction and with a threshold of 0.1% blasts in bone marrow as detected by either flow cytometry or PCR. Thus, we generated a comprehensive overview of patient risk and early response for most patients (Tables 1 and S2).

Subsequently, we analyzed our WES data to identify additional, potentially risk-conferring mutations in our cohort and to delineate the clonality of these mutations by quantifying tumor variant allele frequencies (T-VAFs). Intriguingly, we found several rare entities within our cohort, such as three cases with *UBTF* tandem duplications in samples with trisomy 8 or a normal karyotype and a sample with the *CBFB* GDXY insertion and a normal karyotype. Further hierarchical clustering analysis of T-VAFs revealed three main clusters that largely recapitulated the expected patterns of co-occurrence (Figure 2D). Both *KMT2A* and *NUP98* rearrangements were mostly associated with mutations in *RAS* family genes or *FLT3*. While *KRAS* mutations in our cohort were exclusively associated with *KMT2A* rearrangements, *FLT3* mutations co-occurred with mutations in *WT1* (2 samples) and *UBTF* (3 samples). Most mutations occurred within major subclones, whereas mutations in *KRAS*, *KMT2C*, *BCORL1*, and others were restricted to minor subclones. Given these findings, we classified our cohort with respect to newly described, molecularly unique risk groups.[8] Re-classification mainly affected samples with a normal karyotype, trisomy 8, and the two basket categories for HR and non-HR (Figure S2; Table S2).

Having established our methodology and a cohort that covers the majority of risk-conferring genetic lesions in pedAML, we used scoring based on the relative blast fraction (RBF) to quantify the on-target activity of each compound and concentration and subsequently calculated the approximate area under the dose-response curve for these values to derive RBF-area under the curve (AUC) scores similar to our previous work[26,27] (STAR Methods). Selected case vignettes illustrating the reporting format of our drug sensitivity profiling are displayed in Figure S3. For instance, we investigated a patient with *KMT2A::MLLT1* and a monoblastic M5a phenotype according to the French American British (FAB) classification that responded well to chemotherapy *ex vivo* and achieved stable remission from the first induction cycle onward (Figures S3A–S3D). Another patient in our cohort with an FAB M2 phenotype, classified as high risk according to the AML-BFM 2020 study protocol due to trisomy of chromosome 8 and mutations in *NRAS*, *WT1*, and *FLT3*, showed resistance in our *ex vivo* profiling and did not respond to induction chemotherapy but could eventually undergo successful allogenic hematopoietic stem cell transplantation after late response and has remained in remission since (Figures S3E–S3H).

Inter-patient chemosensitivity heterogeneity is driven by response to chemotherapy and venetoclax combinations

Given these anecdotal observations linking *ex vivo* drug sensitivities and individual clinical courses, we sought to identify the drivers of the functional landscape in pedAML and their associations with clinical characteristics. Across compounds, unsupervised clustering of RBF-AUC scores revealed substantial heterogeneity between patients and initially showed no association with classical clinical parameters such as cellular identity as indicated by FAB class, cytogenetic subgroups, or initial response as quantified by silhouette coefficients (Figures 3 and S4A).

Figure 2. Workflow and cohort overview

(A) Workflow: fresh-frozen samples from 45 patients, which were taken at diagnosis, were profiled for this study. After thawing, samples were profiled with flow cytometry and subjected to image-based drug screening and comprehensive next generation sequencing (NGS) characterization.

(B) Cohort characteristics: the cohort contains samples from all major cytogenetic subgroups with an enrichment for IR and HR cases. Top: patient characteristics. Each column represents a patient. Color codes in the respective columns indicate cytogenetic subgroup, FAB class, risk according to the AIEOP-BFM-AML 2020 study protocol, and measurable residual disease (MRD) after induction 1 and 2, respectively, and are the same as in (C).

(C) Subtype frequencies. Pie charts indicate the relative abundance of the cytogenetic subgroups, FAB classes, risk groups, and MRD positivity after induction 1, respectively.

(D) Clustered heatmap of tumor variant allele frequencies for n = 26 samples where whitelisted protein-altering mutations could be detected.

Table 1. Patient clinical characteristics

ID	FAB	Genetic subgroup	MRD Ind1	MRD Ind2	Risk at diagnosis	Time to death (days)	Time to relapse (days)	Time to SCT (days)	Time to FUP (days)
AML04	M1	NK	ND	ND	IR	534	277	357	534
AML06	M7	*NUP98::KDM5A*	pos	pos	HR	254	154	no SCT	254
AML07	M5b	*KMT2A::MLLT3*	pos	neg	IR	alive	no relapse	no SCT	979
AML12	M2	*NUP98::NSD1*	pos	neg	HR	alive	463	159	463
AML13	M2	*KMT2A::MLLT1*	pos	neg	IR	alive	326	625	938
AML17	M4	*NUP98::NSD1*	pos	pos	HR	alive	no relapse	137	137
AML20	M1	NK	pos	neg	IR	alive	no relapse	no SCT	603
AML23	M5a	*KMT2A::MLLT1*	pos	neg	IR	alive	293	no SCT	1,441
AML24	M4	trisomy 8	ND	ND	IR	alive	421	519	2,191
AML26	M4Eo	*CBFB::MYH11*	ND	ND	SR	alive	no relapse	no SCT	2,457
AML28	M1	NK	ND	ND	IR	472	314	394	472
AML29	M2	*NUP98::NSD1*	ND	ND	HR	alive	397	146	4,190
AML31	M7	*BCR::ABL1*	ND	ND	HR	alive	no relapse	139	2,230
AML34	M6/M7	*NUP98::KDM5A*	pos	pos	HR	alive	no relapse	no SCT	258
AML37	M5a	*KMT2A::MLLT1*	neg	neg	IR	alive	no relapse	no SCT	1,091
AML39	M4	*DEK::NUP214*	pos	pos	HR	alive	no relapse	134	322
AML43	M2	NK (*CEBPA*dm)	ND	ND	SR	alive	no relapse	no SCT	1,735
AML44	M7	*NUP98::KDM5A*	pos	neg	HR	alive	no relapse	no SCT	911
AML47	M4Eo	*CBFB::MYH11*	neg	neg	SR	alive	no relapse	no SCT	237
AML50	M7	*CBFA2T3::GLIS2*	pos	neg	HR	alive	no relapse	no SCT	1,368
AML52	M5	*KMT2A::MLLT10*	neg	neg	HR	alive	no relapse	153	956
AML54	M5	*KMT2A::MLLT3*	neg	neg	IR	342	237	175	342
AML55	M5	*NUP98::KDM5A*	neg	neg	HR	alive	no relapse	181	543
AML57	M2/MDS	trisomy 8	pos	pos	IR	alive	no relapse	67	246
AML58	M2	NK (*CEBPA*dm)	neg	neg	SR	alive	no relapse	no SCT	555
AML59	M4Eo	*CBFB::MYH11*	pos	neg	SR	alive	no relapse	no SCT	2,313
AML60	M4Eo	*CBFB::MYH11*	neg	neg	SR	alive	1,069	1,175	1,667
AML62	M4	*DEK::NUP214*	ND	neg	HR	alive	no relapse	134	1,362
AML63	M5a	*NUP98::NSD1*	neg	neg	HR	alive	973	973	1,635
AML64	M1	NK	neg	neg	IR	alive	no relapse	no SCT	1,868
AML65	M5	*KMT2A::MLLT3*	neg	neg	IR	alive	no relapse	no SCT	410
AML66	M4Eo	*CBFB::MYH11*	neg	neg	SR	alive	no relapse	no SCT	1,207
AML67	M5a	*KMT2A::MLLT3*	neg	neg	IR	alive	no relapse	no SCT	1,730
AML74	M4	NK	pos	neg	IR	745	303	no SCT	745
AML76	M5	*KMT2A::MLLT3*	neg	neg	IR	alive	no relapse	no SCT	1,583
AML77	M4	trisomy 8	pos	pos	IR	66	no relapse	63	66
AML81	M4	Other	ND	ND	IR	alive	no relapse	no SCT	2,961
AML82	M1	NK	neg	neg	IR	alive	798	no SCT	1,358
AML84	M7	Complex	neg	neg	HR	alive	no relapse	no SCT	607
AML87	M7	NK	pos	neg	IR	949	413	no SCT	949
AML88	M5	monosomy 7	neg	neg	HR	alive	no relapse	32	2,190
AML89	M2	*RUNX1::RUNX1T1*	ND	ND	SR	alive	no relapse	no SCT	3,499
AML93	M0	*ETV6::MNX1*	ND	ND	HR	668	480	100	668
AML95	M4	inv(3)(q21q26) *RPN1::MECOM*	ND	pos	HR	319	190	93	319
AML98	M4Eo	*CBFB::MYH11*	ND	neg	SR	alive	no relapse	no SCT	2,343

Overview of patient clinical characteristics for each retrospective sample. Abbreviations are as follows: FAB, French American British classification; NK, normal karyotype; MRD, measurable residual disease; SR, standard risk; IR, intermediate risk; HR, high risk; SCT, hematopoietic stem cell transplantation; FUP, follow-up; ND, not determined.

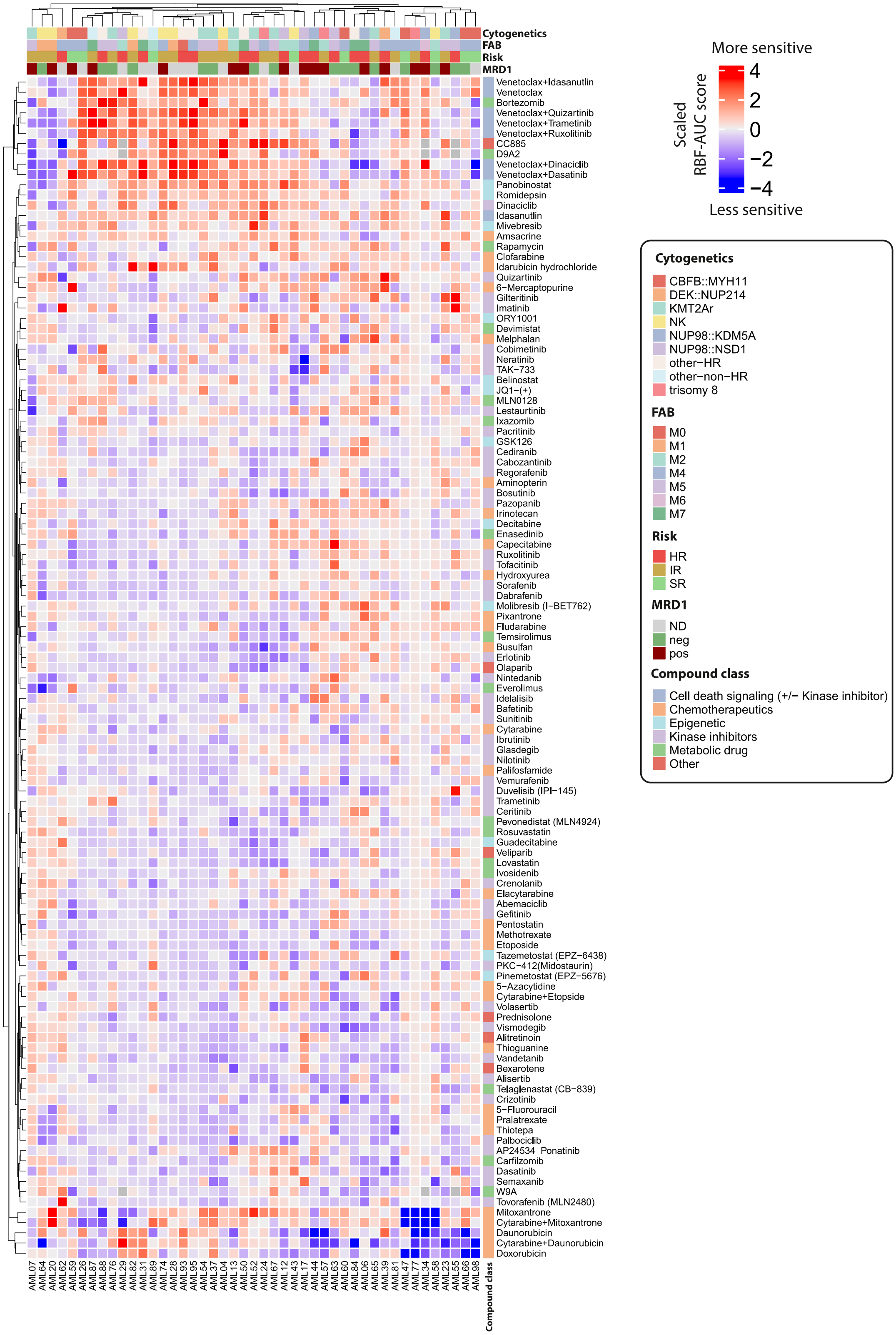

(legend on next page)

Clustering of compounds indicated two main clusters that seemed to drive variation. One cluster comprised venetoclax combinations, together with bortezomib, the SLC9 degrader D9A2, and the cereblon modulator CC885, whereas the other comprised first-line chemotherapy compounds such as daunorubicin alone, mitoxantrone alone, or either drug in combination with cytarabine. While daunorubicin and doxorubicin clustered closely together and were in the same cluster as mitoxantrone, idarubicin was placed in a different cluster apart from these drugs. This is also reflected in the correlation coefficients for these compounds, where scaled RBF-AUC scores of daunorubicin and doxorubicin responses correlate significantly with each other, but this is not the case for the other anthracyclines, which may reflect different off-target toxicities of these drugs (Figure S4B). These compounds were also among the compounds with the highest variation of RBF-AUC values across all tested compounds (Figure S4C), indicating that these were drivers of variation. Previous work has already established a link between apoptotic priming and response to chemotherapy and more specifically topoisomerase inhibitors.[42] Given the additional established link between apoptotic priming and venetoclax response,[43] we investigated potential correlations between chemotherapy response and response to venetoclax. We did not observe any strong correlations between venetoclax and chemotherapy for our scaled RBF-AUC score, which may reflect the differential off-target effects between these compounds. However, using the AUC score on the absolute blast fraction, without consideration of the personalized healthy (non-malignant) control cells, revealed significant correlations between venetoclax response and response to the topoisomerase inhibitors daunorubicin and idarubicin, and the combinations of cytarabine with daunorubicin or mitoxantrone, whereas responses to cytarabine did not correlate with venetoclax responses using either quantification approach (Figures S5A and S5B), indicating that apoptotic priming may also be a determinant of chemotherapy response in pedAML but not necessarily informative of off-target effects. Intriguingly, the two targeted FLT3 inhibitors quizartinib and gilteritinib were also among the compounds driving variation, but neither of these agents clustered with venetoclax combinations or standard chemotherapeutics of the induction regimen. Thus, we investigated the correlations of RBF-AUC scores for the compounds that drive variation. Compounds in the venetoclax combination cluster formed one cluster of strongly correlating compounds and correlated significantly with several chemotherapy drugs and combinations, further indicating that these compounds jointly drive variation. Intriguingly, the FLT3 inhibitors gilteritinib and quizartinib correlated negatively with a majority of compounds in that cluster, indicating that combined BCL2 inhibition and FLT3 inhibition posed opposing axes of vulnerability in our cohort (Figure 4A). These intriguing correlations prompted us to further investigate the correlations between different drug classes (STAR Methods). By filtering for the most consistent correlations between drug-class pairs, we identified general trends of positive correlations between HDAC inhibitors and proteasome inhibitors with venetoclax combinations and negative correlations between FLT3 inhibitors and venetoclax combinations as well as several other consistent pairs (Figure 4B). These results indicated that venetoclax may not be effective in patients who benefit from FLT3 inhibitors, whereas proteasome inhibitors, HDAC inhibitors, and venetoclax combinations may be effective in similar patient populations.

Chromatin accessibility analysis delineates cellular hierarchy states

Given these observations, we set out to identify the molecular drivers of the observed chemosensitivity patterns. Recent work has identified distinctive associations of known and newly defined genomic classes with leukemic stem cell states.[8] Another recent study identified a trajectory toward a more primitive cell state upon relapse.[9] Together, these results indicated that leukemic cells that are more similar to hematopoietic stem cells (HSCs) may drive resistance to chemotherapy. Therefore, we hypothesized that cellular differentiation states and their underlying gene-regulatory phenotypes may drive differential responses to induction chemotherapy and that chemosensitivities in pedAML undergo profound changes along the differentiation trajectory.

Accordingly, we leveraged our ATAC-seq data to gain insights into cellular differentiation states in our cohort. First, we mapped ATAC-seq data against a healthy reference.[44] Principal component analysis (PCA) after batch correction placed a majority of pedAML samples in proximity to several progenitor populations (Figure 5A), whereas samples with an FAB M7 phenotype with characteristic lesions such as *NUP98* rearrangements and the *CBFA2T3::GLIS2* fusion clustered mainly with each other in proximity to erythroblasts and megakaryocyte erythroid progenitor cells. Variation seemed to be mainly driven by differences between undifferentiated progenitor populations and differentiated lymphoid cells on the first principal component and differences between M7 pedAML samples and monocytes on the second principal component, suggesting that the pedAML cells largely assume precursor-like identities.

Given these observations and previous data that suggested a transition toward more stem cell-like phenotypes upon relapse,[9] we applied a support vector classifier to map pedAML samples in our cohort to their closest healthy counterpart. Our model was able to reliably distinguish all healthy cell types except multipotent progenitors and hematopoietic stem cells (Figure S6A). Overall, the model classified a majority of samples as granulocyte-macrophage progenitors (GMPs) (n = 19) or monocytes (n = 8), followed by megakaryocyte erythroid progenitors (MEPs) (n = 5) and common myeloid progenitors (CMPs) (n = 5). HSCs, erythroblasts, and lymphoid-primed multipotent progenitors (lpMPPs) were predicted only once (Figure S6B). We further confirmed the validity of these predictions using

Figure 3. The chemosensitivity landscape of pedAML

Scaled RBF-AUC scores for n = 45 samples of the retrospective cohort and each compound and combination.

Positive values (red) indicate sensitivity. Negative values (blue) indicate resistance. Row and column annotations are indicated in the legend on the right. Compounds were tested at three concentrations with two technical replicates per concentration (STAR Methods).

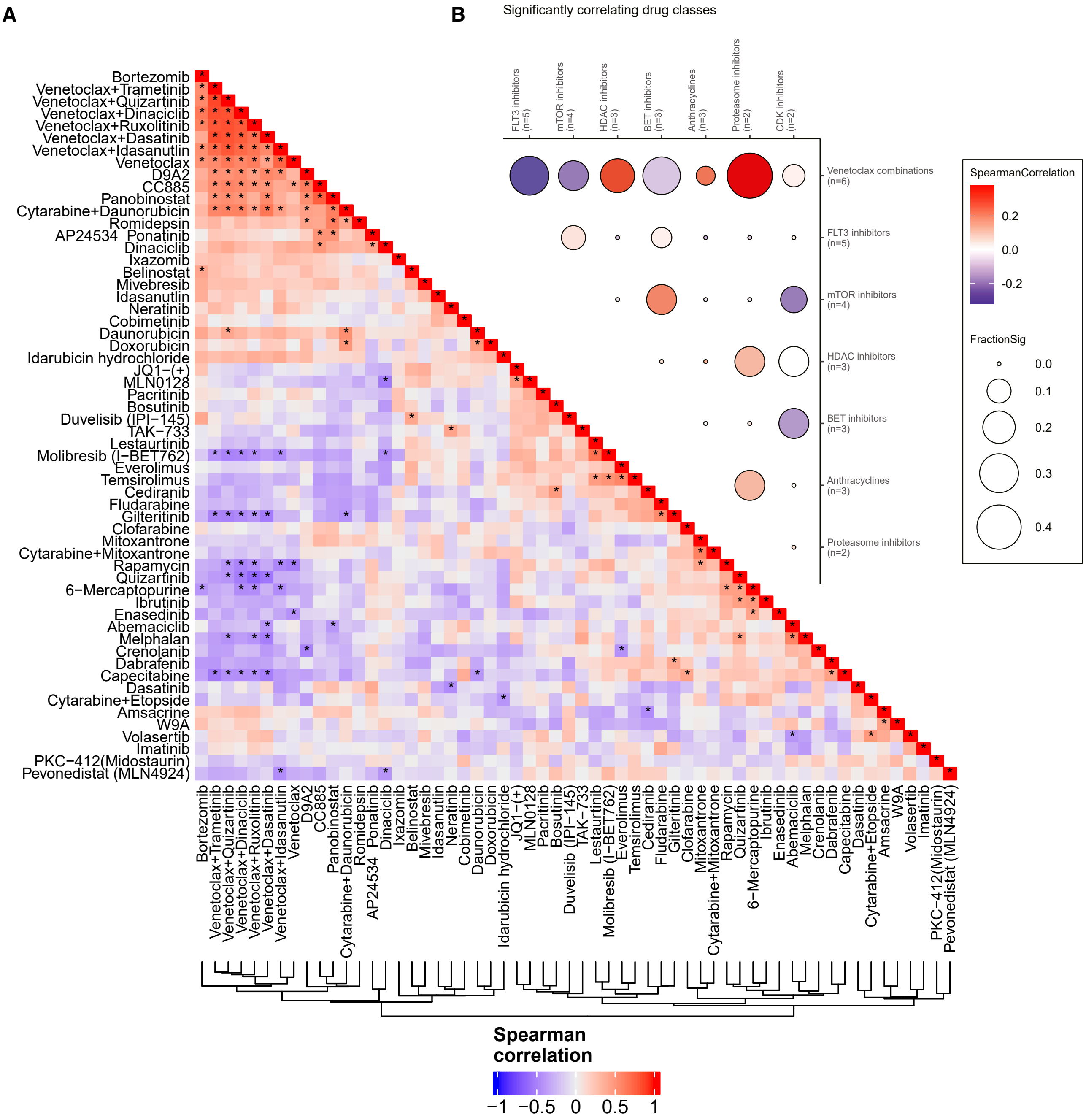

Figure 4. Correlations analyses identify axes of vulnerability

(A) Pairwise Spearman correlations of RBF-AUC scores for the 58 drugs with activity in at least 3 samples. Drugs are ordered by hierarchical clustering. Significant correlations after Benjamini-Hochberg multiple testing correction ((p_{adj} < 0.05) are highlighted with a star.

(B) Bubble plot of the seven drug classes with consistently correlating pairs where all pairs have either positive or negative correlations. Colors indicate the average Spearman correlations across all pairs between the two classes. Sizes indicate the fraction of significant pairs after Benjamini-Hochberg multiple testing correction (p_{adj} < 0.05).

chromVAR analysis,[45] which demonstrated strong associations of *CEBP*-family transcription factor (TF) motifs with monocytic identities, *RUNX*-family motifs with precursor identities, and *GATA*-family motifs with erythroid and precursor identities (Figure S6C). ChromVAR scores for the respective TFs also correlated strongly with expression of the respective genes, further supporting the validity of cell type and TF analyses (Figure S6D). Globally, we observed intriguing changes in TF activity along the differentiation trajectory, with partial overlaps along specific trajectories. HSC-like states were associated

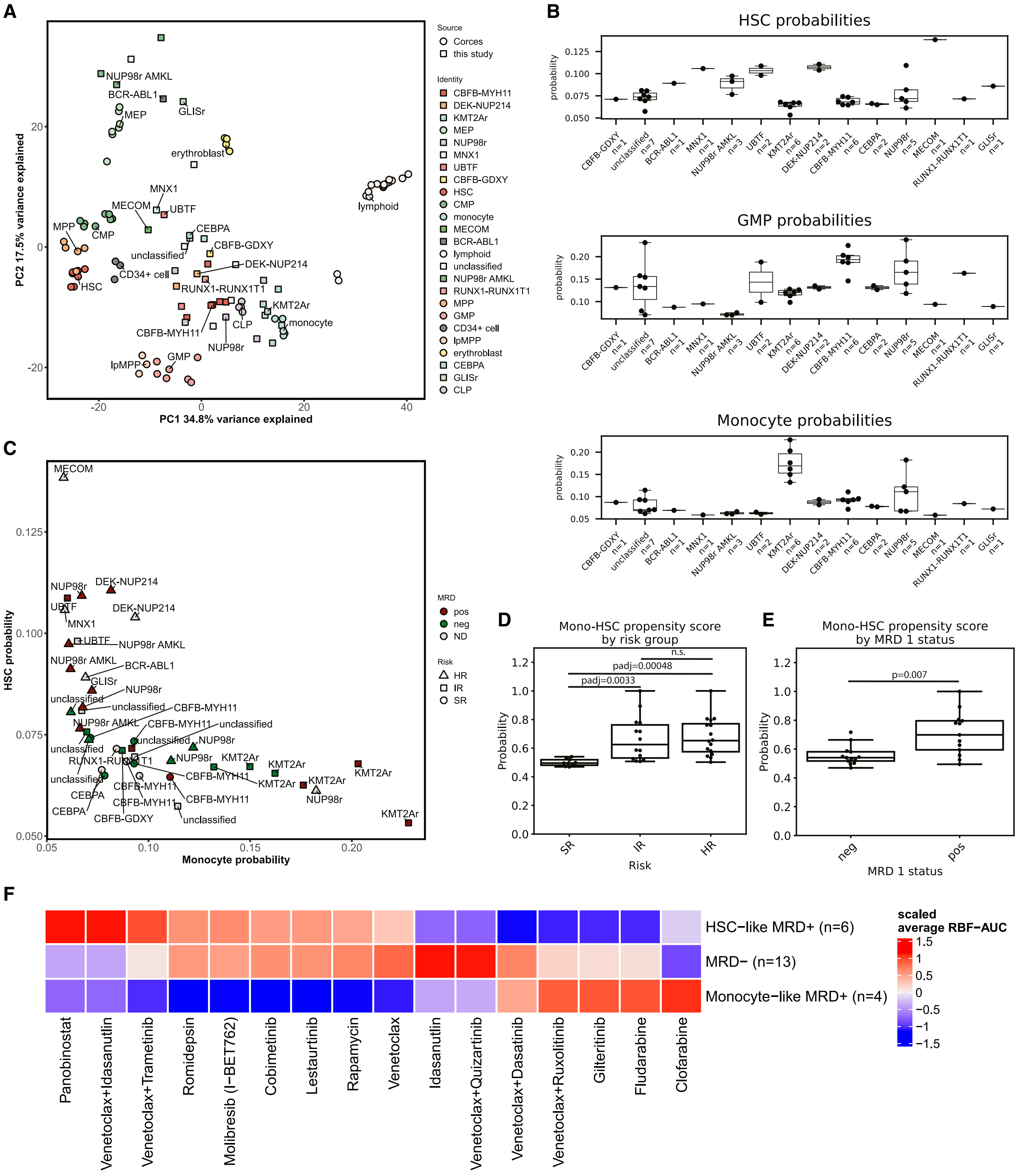

Figure 5. Cellular differentiation states are associated with unique drug response signatures

(A) PCA plot for this study (*n* = 39) and the dataset from Corces et al. (*n* = 69) using the 1,000 most variable consensus regions. Annotations indicate healthy precursors or groups defined by Umeda et al., respectively. Unclassified indicates all samples that could not be classified according to the scheme; lymphoid indicates B cells, T cells, and NK cells. MEP, megakaryocyte erythroid progenitor; HSC, hematopoietic stem cell; CMP, common myeloid progenitor; MPP, multipotent progenitor; GMP, granulocyte-macrophage progenitor; lpMPP, lymphoid-primed multipotent progenitor; CLP, common lymphoid progenitor.

(legend continued on next page)

with GATA-family TFs and TFs of the forkhead box (FOX) family (Figure S7A). The association with GATA-family TFs was largely retained for the CMP-, MEP-, and erythroblast-like states, whereas the association with FOX TFs partially changed and was finally lost in the erythroblast state (Figures S7B–S7D). Intriguingly, FOX TFs have mainly been implicated in lymphoid homeostasis,[46] indicating that lymphoid gene-regulatory programs may be co-opted for leukemic development in these states.[9] Monocytic and GMP-like states on the other hand were more associated with activity of interferon regulatory factors (IRFs) and STAT or CEBP-family TFs, respectively (Figures S7E and S7F), whereas lpMPP-like states had a distinct pattern of TF co-regulation (Figure S7G).

Intriguingly, the comparison of probabilities between genetic groups revealed group-specific patterns of similarity that recapitulated other recent work[8] (Figure 5B): *UBTF*-TD and *DEK::NUP214* leukemias were predicted to be more similar to HSCs than others, and *CBFB::MYH11* leukemias more similar to GMPs. Furthermore, *KMT2A*-rearranged samples in our cohort were predicted to be particularly similar to monocytes when compared to other samples.

Cellular hierarchy states are associated with distinct clinical responses and *ex vivo* chemosensitivities

Given these associations with genetically defined risk groups, we hypothesized that the position along the differentiation trajectory from HSCs to monocytes may be associated with patient risk and outcome. Plotting the probabilities of HSCs and monocytes revealed an intriguing pattern, where the majority of samples had low probabilities for both HSC and monocyte identities. Samples with MRD or higher risk seemed to deviate from this state toward either a monocytic or an HSC-like state (Figure 5C). A more monocyte-like state was associated with intermediate- to high-risk *KMT2A*-rearranged AMLs, whereas a more HSC-like state was associated with *NUP98* fusions and *UBTF* tandem duplications. These data indicated that there was a higher propensity for high-risk genetics and non-response toward either end of the differentiation spectrum such that both the most differentiated and least differentiated cell states are associated with higher patient risk. Thus, we aimed to quantify this phenomenon by devising a Mono-HSC propensity score that quantifies this propensity away from the average phenotype in our cohort and toward a more HSC-like or more monocytic identity as $\max\left(\frac{P_{mono}}{\max(P_{mono})}, \frac{P_{HSC}}{\max(P_{HSC})}\right)$ with the monocyte probability P_{mono} and the HSC probability P_{HSC}. In line with our expectations from the observed genetics at either ends of the differentiation spectrum, we found this score to be significantly associated with both patient risk and MRD after induction 1 (Figures 5D and 5E). Additonal correlative analysis of our differentiation state probabilities and CIBERSORT scores from a recent large-scale cohort study identified similar associations between genetic risk groups and differentiation states. We found significant correlations for primitive states and GMP-like states, whereas only the pro-monocytic state correlated significantly with our monocyte probability but not the monocyte score derived in the related work (Figures S8A–S8G). Devising a similar Mono-HSC propensity score as described earlier for the primitive score and the pro-monocytic score, we found significant differences between MRD-positive and -negative samples and between the different risk groups as defined by the AIEOP-BFM-AML study group (Figures S8H–S8I).

Associations between cellular differentiation state and patient risk and drug responses have been observed in a number of works in both pediatric and adult AML.

Given these associations of cellular hierarchies and clinical response, we hypothesized that these phenotypic differences would be mirrored in the blast chemosensitivities. Indeed, analysis of the top compounds for the more HSC-like MRD-positive samples (HSC-MRD+) and the more monocyte-like MRD-positive samples (Mono-MRD+) revealed distinct profiles, with panobinostat and the combination of venetoclax and idasanutlin as specific vulnerabilities of HSC-MRD+ samples, whereas chemotherapeutics such as irinotecan and thiotepa inhibited Mono-MRD+ samples more specifically. Previous research has identified monocytic cell states and RAS mutations as drivers of venetoclax resistance in adult AML,[47,48] prompting us to test whether there would be a differential influence of *RAS* mutations or monocytic cell states on venetoclax sensitivity. Indeed, our analysis indicated lower sensitivity to venetoclax in RAS-mutated monocytic samples than in GMP-like *RAS* wild-type samples or other samples (Figure S9A). Notably, combinations of venetoclax had an effect that was highly dependent on the combination partner, and combinations with kinase inhibitors showed less activity in HSC-MRD+ samples (Figure 5F). We further expanded this analysis to all predicted hematopoietic differentiation states and found venetoclax sensitivity to be most strongly associated with HSC and erythroblast states, whereas other states seemed to be more resistant to venetoclax as a single agent (Figure S9B). Intriguingly, the combinations of venetoclax with other agents had additional higher activity in CMPs, indicating that combination with other agents broadens the efficacy spectrum to more gene-regulatory backgrounds. Several compounds, such as quizartinib or pazopanib, showed inconsistent activity with respect to differentiation states, indicating that for some of these, other drivers may be more relevant for response.

Chemosensitivity data are predictive of patient response

To better understand the potential clinical utility of our drug sensitivity profiling approach, we set out to further investigate the

(B) Boxplots of probabilities per genetic group as defined by Umeda et al. Boxes indicate the quartiles of the distribution and whiskers extend to points that are within 1.5 interquartile ranges. Dots indicate individual samples (n = 39) Top: probabilities for hematopoietic stem cells (HSCs). Middle: probabilities for granulocyte-macrophage precursors (GMPs). Bottom: probabilities for monocytes.

(C) Scatterplot of HSC probability (x axis) against monocyte probability (y axis), colored by MRD1 (n = 39).

(D and E) Scaled probability of monocytic/HSC identity by risk group (n = 39) and MRD status (n = 26). Significance was tested with the Mann-Whitney U test. Boxes and whiskers are indicated as in (B). Dots indicate values for individual samples.

(F) Heatmap of scaled average RBF-AUC scores for top 10 drugs of MRD-positive monocyte-like and MRD-positive HSC-like samples.

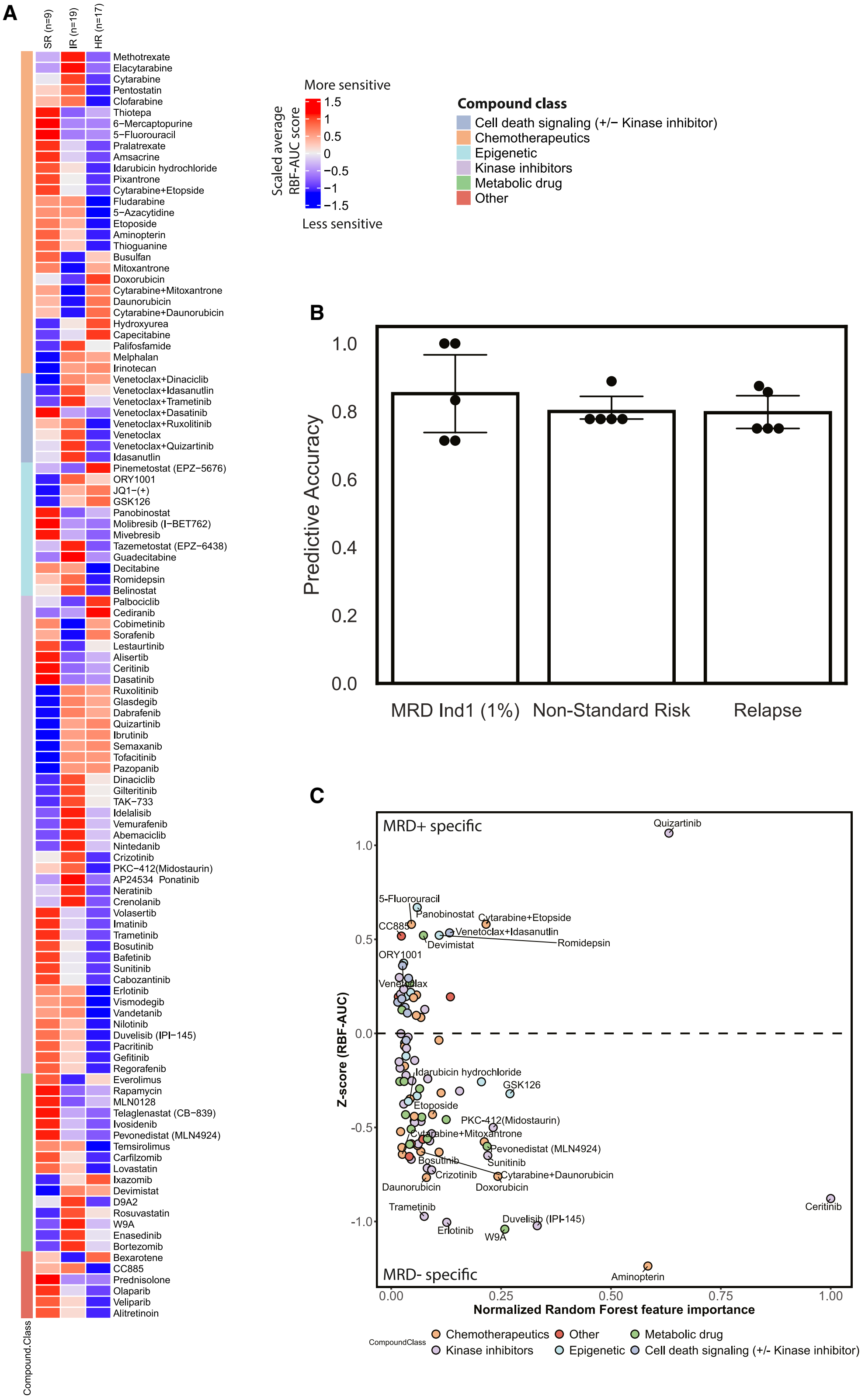

(legend on next page)

CellPress
OPEN ACCESS

relationship between chemosensitivity, as quantified by RBF-AUC scores, and key clinical characteristics such as MRD, non-standard risk as defined in the AIEOP-BFM-AML 2020 trial, and early relapse. Specifically, we aimed to identify key differences in sensitivity patterns between risk groups by comparing RBF-AUC values and generating predictive models for MRD, risk, and early relapse.

We hypothesized that chemosensitivity differences between risk groups, as defined by the AIEOP-BFM-AML study group, may reveal actionable vulnerabilities. Analysis of responses by risk group revealed intriguing differences that were often concordant with compound mechanism of action between the standard-risk (SR), IR, and HR groups (Figure 6A). SR samples showed stronger responses to chemotherapy than HR samples in a majority of cases, particularly for nucleoside analogs such as 6-mercaptopurine or 5-fluorouracil, whereas cytarabine and anthracyclines such as idarubicin and daunorubicin seemed to be less specific to individual risk groups (Figure 6A). Furthermore, several targeted agents that are mainly considered in high-risk and relapse settings did not show the strongest activity in the HR group. Venetoclax alone and in combination most frequently showed activity in the IR group, and tyrosine kinase inhibitors showed stronger activity in the SR and IR groups than in the HR group. We found only a few vulnerabilities that were specific to the HR group, such as sensitivity to the CDK inhibitor palbociclib and the DOT1L inhibitor pinometostat (Figure 6A). Palbociclib is a CDK4/6 inhibitor, and CDK6 has been shown to be an essential target in both *NUP98*- and *KMT2A*-rearranged AML.[49,50] *NUP98* rearrangements are the most common genetic background among high-risk samples in our cohort.

More generally, our data indicate that chemotherapy sensitivity varies broadly by agent and that response profiles of genetically defined risk groups may only partially converge on individual targets, further emphasizing that drug sensitivity data do not simply recapitulate genetically defined risk. In line with these observations, our analysis of cluster concordance between drug sensitivity profiles and genetic status did not yield any associations between the two but rather indicated MRD and patient risk to be similarly concordant with drug sensitivity profiles (Figure S3A), indicating that drug sensitivity profiles reflect functional aspects of patient risk that are not recapitulated by genetics.

Given these differences between risk groups, we hypothesized that simple machine learning models may be able to predict key clinical parameters from RBF-AUC scores. Thus, we aimed to predict MRD status, non-standard risk, and early relapse from drug sensitivity profiles using RBF-AUC scores as input for random forest models in a cross-validation setting. Predictive accuracies ranged from 85.2% for the prediction of 1% MRD after induction 1 to 79.9% for non-standard risk, further highlighting the predictive power of our approach (Figure 6B). Accuracy scores for the prediction of early relapse—defined as relapse within the first 12 months after diagnosis—were 79.2% and therefore slightly lower than those for MRD, indicating that chemosensitivity screening may be most predictive for events that are closer in time not at least because of inter-individual divergences in later treatment, such as stem cell transplantation. Overall, these results indicate that drug sensitivity profiling may have clinical utility for the early identification of non-responders and thus provide an additional tool for patient risk stratification—beyond the identification of promising treatments in high-risk settings.

Analyzing the relationship between feature importances and relative inhibition revealed drugs such as the antifolate aminopterin and the receptor tyrosine kinase inhibitors erlotinib and ceritinib to be more active in these MRD-negative samples (Figure 6C). The increased sensitivity to the antifolate aminopterin may indicate a stronger dependency on folate metabolism in low-risk patients, whereas the increased sensitivities to the unspecific tyrosine kinase inhibitors erlotinib and ceritinib may be more indicative of a broad dependency on tyrosine kinase signaling. The higher sensitivities in MRD-negative samples overall may indicate an increased susceptibility to apoptosis, which has been linked to better patient outcomes in earlier studies.[51] These results further support the observed predictivity of *ex vivo* testing for response from previous studies[26,27,30,32] and provide additional rationale for early integration of functional screening to predict response and identify early vulnerabilities in high-risk disease.

DISCUSSION

Because of its rigorous logic, precision and personalized medicine have inspired the biomedical community over the last decade to achieve a patient-specific treatment rationale.[52] Indeed, our molecular mechanistic understanding of disease in a variety of cancers has tempted us to consider the granular stratification of patients based on genomic data and more recently additional -omics as an achievable goal.[52] However, recent experiences in adult cancers such as adult leukemia and lymphoma indicate that genetics-focused precision medicine approaches may not faithfully recapitulate the functional heterogeneity within samples from patients with cancer.[25] These observations imply that there are additional determinants of functional drug responsiveness that would be key to integrate into precision medicine-based approaches. Hence, the field of functional precision medicine has started to tackle these challenges by direct functional testing of cancer cells, either as an

Figure 6. Chemosensitivity screening is predictive of key clinical parameters

(A) Heatmap of scaled averaged responses per AIEOP-BFM-AML risk group as quantified by RBF-AUC scores for $n = 45$ samples in our cohort.

(B) Accuracy scores for each prediction target of 1% MRD ($n = 32$ evaluable samples), non-standard risk ($n = 45$ evaluable samples), and early relapse ($n = 39$ evaluable samples) over the 5-fold cross-validation. Dots indicate accuracy scores per fold. The height of the bars indicates the mean accuracy over all folds, and error bars indicate the 95% confidence interval.

(C) Scatterplot of Z score of inhibition for MRD1-positive (top) compared to MRD-negative (bottom) samples (y axis) against random forest feature importance (x axis), where compounds to the right contribute more to predictivity based on cross-validation over $n = 32$ evaluable samples. Compounds are colored by compound class as in Figures 1 and 4.

alternative or conversely as a complementary approach to genomics-based precision medicine approaches.[53,54] However, the heterogeneity and relatively small sample sizes in pediatric tumors challenge the notion of clinical trial designs with genetics-based disease classification and treatment strategies.

In this study, we set out to study pedAML as a model disease to assess the feasibility of state-of-the-art image-based chemosensitivity profiling for integration into the clinical management of pedAML and to elucidate functional-genomic patterns of patient risk that may transcend genetically defined risk groups. Building upon our previously established approach with demonstrated clinical efficacy[26,27]—albeit with a relatively short incubation time—we here established an image-based screening platform that enables fast turnover times for a vast array of drugs with clinical relevance, such as chemotherapeutics in first-line therapy, compounds that target epigenetic regulators, BCL2 inhibitors, and a vast number of kinase inhibitors. Recent efforts have pursued similar goals with flow cytometry-based assays in diverse hematological malignancies[32,39–41,55] with a similar intention of employing single-cell readouts to define leukemic blast-specific drug response measurements. In our own experience, an image-based drug response platform as showcased here comes with the need of professional computational image analysis skills but arguably bears advantages when it comes to scalability with regard to costs per sample or the relative ease to expand or adapt platforms to individual patients or novel drugs to be included in a screen. The application of simple machine learning models enabled us to accurately predict key clinical parameters, such as non-standard risk, and MRD after the first induction cycle from drug sensitivity profiles, thus demonstrating the potential applicability of functional screening approaches for the early detection of patients at risk. While the detailed mechanistic and biological basis of the predictivity of drug sensitivity profiling is still a subject of active research and cannot be sufficiently addressed in this study due to sample size and the lack of a validation cohort, our work highlights the potential use of advanced drug sensitivity profiling for early identification of patients at risk and the identification of promising treatments. Future studies may overcome these hurdles and validate these findings by using this predictive approach in a prospective setting, where *ex vivo* screening will be performed at diagnosis and predictiveness can be evaluated at the respective time points for risk stratification, MRD assessment, and upon eventual relapse. Thus, while most functional profiling studies focus on patients with late-stage disease,[26–28,30] our study provides a rationale for testing patients at early time points, in line with the observation that early response in pedAML is a long-term predictor of outcome.[21–24]

The genetic heterogeneity of pedAML—with recent work distinguishing more than 20 genetically distinct entities[8,56]—makes it a particularly attractive target for functional precision medicine approaches. These approaches enable both targeted personalized treatments[26] and the elucidation of the corresponding disease-specific functional landscapes.[54,57,58] Several studies have highlighted functional heterogeneity in pedAML and other pediatric cancers[31,59] but did not identify any functional groupings that could be motivated from a molecular perspective. These heterogeneous results may be due to the relatively high percentages of healthy cells that are being screened together with blasts in these assays. In contrast, our targeted and blast-specific approach indicates that response heterogeneity converges on sensitivity to conventional chemotherapy, HDAC inhibition, or sensitivity to BCL2 inhibition with or without kinase inhibition. Future studies will need to also comparatively test different emerging molecular and drug testing platforms with different treatment times and culturing conditions to understand platform- or technology-dependent advantages and disadvantages of various approaches.

Heterogeneity in treatment responses within genetically defined risk groups has been observed in several large pedAML cohorts.[8,21] However, the determining factors of this functional heterogeneity have remained elusive. On the epigenetic level, we observe that a propensity toward either more monocytic or more HSC-like states is indicative of risk and poor response. Associations of HSC-like gene expression signatures with patient risk in pediatric and adult AML are well known,[60–62] and recent data indicate a trajectory toward more primitive states upon relapse.[9] Several targeted treatment options for HSC-like states have been proposed,[63] but successes have been limited to few agents such as venetoclax. Indeed, we observed higher sensitivity to the combination of venetoclax and idasanutlin in HSC-like states and additionally higher sensitivity to the HDAC inhibitor panobinostat. HDAC inhibitors have been previously applied in clinical trials, but the results have been mixed,[64,65] which may be due to the lack of predictive biomarkers. Our results indicate that HDAC inhibition may be a promising route to target HSC-like states, thus providing a rationale for targeted evaluation of this agent. Furthermore, we observed higher sensitivity to the chemotherapeutic agents clofarabine and fludarabine in more monocyte-like states, indicating that especially this subset may profit from these chemotherapeutics. Hence, our results indicate that cellular differentiation is a targetable state in pedAML and suggest that selective chemotherapy may improve outcome in more differentiated leukemias and that targeted treatments with BCL2 or HDAC inhibition may improve outcome in more HSC-like leukemias. Collectively, our results implicate cellular differentiation states as a driver of treatment response heterogeneity and identify cell state-specific vulnerabilities that may enable personalized targeted treatments of poor responders.

Overall, our study underlines the feasibility and predictivity of *ex vivo* functional profiling for pedAML and other malignancies. The implementation of functional profiling in clinical practice has been in continuous discussion, and recent technological advances finally enable functional profiling in prospective settings.[26,54,66,67] Our data demonstrate that functional profiling enables risk prediction and the identification of targeted treatments for molecularly defined subgroups at even earlier time points, clearly showcasing the added value of functional profiling for patient stratification and response assessment. Looking forward, we envision that systematic application of functional profiling over the disease course will enable earlier identification of patients at risk and of promising drugs and has the potential to improve treatment outcome for broader patient populations.

Limitations of the study

While our study highlights the potential of advanced drug sensitivity profiling at diagnosis for patient stratification and risk

prediction and reveals intriguing associations of drug response and cellular differentiation state in pedAML, it does come with limitations. Given the rarity of pedAML, our patient cohort—while covering the broad spectrum of pedAML driving lesions—bears limitations for identifying associations between genetic background and drug response due to insufficient statistical power. Furthermore, our study is retrospective and does not provide an independent validation cohort to fully validate our findings. Prospective studies in larger cohorts are needed to demonstrate the utility of functional profiling in the clinics. Studies like ours bridge this gap by demonstrating the feasibility of functional profiling for patient stratification and treatment prioritization. Future efforts to implement functional profiling for personalized treatment prioritization will require international collaboration. Such efforts may further advance the substantial improvements in outcome for patients with pedAML that large-scale international trials achieved over the past decades.[3,5,21] Despite these limitations, our study provides substantial new data on the functional and epigenetic landscape of pedAML and provides a template for establishing advanced functional profiling for rare pediatric malignancies.

RESOURCE AVAILABILITY

Lead contact

Further information and requests for resources and reagents should be directed to and will be fulfilled by the lead contact Dr. Kaan Boztug (kaan.boztug@ccri.at).

Materials availability

This study did not generate any new unique reagents.

Data and code availability

- ATAC-seq data of sorted blasts have been deposited in the Gene Expression Omnibus (GEO) under the accession number GEO: GSE282258. RNA-seq data of sorted blasts have been deposited in GEO under the accession number GEO: GSE282423. Raw exome-sequencing data have been deposited at the European Genome Phenome Archive (EGA) under the accession number EGA: EGAD50000001572. To request access, please submit an access request via the EGA website or contact Dr. Kaan Boztug (kaan.boztug@ccri.at). Raw imaging data will be made available upon request and uploaded to the appropriate platforms by the time of publication.
- Code and processed data to reproduce all figures in the manuscript are stored in a GitHub repository under https://github.com/Boztug-lab/pedaml_screening.
- Any additional information required to reanalyze the data reported in this work is available from the lead contact upon request.

ACKNOWLEDGMENTS

We thank all patients and families for their participation in the study. We further thank Michael Schuster and Christoph Bock from the Biomedical Sequencing Facility of the Research Center for Molecular Medicine of the Austrian Academy of Sciences (CeMM) for sequencing services and technical input. Additionally, we thank Anna Koren, Monika Malik, and Stefan Kubicek of the Molecular Discovery Platform of CeMM for their technical input and providing compounds and printed screening plates. We further thank Nora Mühlegger and Chiara Wertz for providing documentation of patient clinical characteristics. We also thank André Rendeiro for critical input on epigenetic analyses and Florian Grebien for invaluable critical review and discussion of the manuscript. This study was supported by funds from the Austrian Science Fund (FWF) (DOI: https://doi.org/10.55776/KLI1056 to K.B.) and by additional intramural funding by the St. Anna Children's Cancer Research Institute including donations. For open access purposes, the author has applied a CC BY public copyright license to any author accepted manuscript version arising from this submission.

AUTHOR CONTRIBUTIONS

Conceptualization, B.H., M.M.-G., M.N.D., G.S.-F., and K.B.; methodology, B.H., P.Z., R.J.-H., A.F., A.S.-R., F.K., S.G., C.R., C.C., and P.R.; software, B.H., P.Z., C.C., and P.R.; formal analysis, B.H., M.M.-G., P.Z., and C.C.; investigation, B.H., M.M.-G., P.Z., R.J.-H., A.F., A.S.-R., F.K., S.G., C.R., and C.C.; resources, M.N.D., G.S.-F., and K.B.; data curation, B.H., M.M.-G., P.Z., and A.F.; writing – original draft, B.H. and K.B.; writing – review and editing, B.H., M.N.D., G.S.-F., and K.B.; visualization, B.H.; supervision, M.N.D., G.S.-F., and K.B.; funding acquisition, B.H., M.M.-G., M.N.D., G.S.-F., and K.B.

DECLARATION OF INTERESTS

G.S.-F. has a patent EP3704484A1 pending and patent EP3198276A1. Both are licensed to Exscientia GmbH.

STAR★METHODS

Detailed methods are provided in the online version of this paper and include the following:

- KEY RESOURCES TABLE
- EXPERIMENTAL MODEL AND STUDY PARTICIPANT DETAILS
- METHOD DETAILS
  - Drug treatment
  - Staining and image acquisition
  - Image analysis
  - Nucleus segmentation and segmentation model training
  - Cell type prediction
  - Viability prediction
  - Hyperparameter optimization for adversarial autoencoders
  - Benchmarking of image-based screening and CellTiterGlo data
  - Processing of dose response values and compound activity scoring
  - Drug-drug correlation analysis
  - Immunophenotyping for identification of markers for leukemic blasts
  - FACS staining for cell sorting
  - Cell number and cell viability assessment via flow cytometry
  - Transcriptome, exome and accessible chromatin sequencing
  - mRNA sequencing data analysis
  - ATAC-seq analysis
  - WES analysis
  - Predictions of healthy cell types and clinical variables
- QUANTIFICATION AND STATISTICAL ANALYSIS
- ADDITIONAL RESOURCES

SUPPLEMENTAL INFORMATION

Supplemental information can be found online at https://doi.org/10.1016/j.xcrm.2025.102304.

Received: November 28, 2024
Revised: May 18, 2025
Accepted: July 24, 2025
Published: August 20, 2025

REFERENCES

1. Bolouri, H., Farrar, J.E., Triche, T., Jr., Ries, R.E., Lim, E.L., Alonzo, T.A., Ma, Y., Moore, R., Mungall, A.J., Marra, M.A., et al. (2018). The molecular landscape of pediatric acute myeloid leukemia reveals recurrent structural alterations and age-specific mutational interactions. Nat. Med. *24*, 103–112.

2. Bonaventure, A., Harewood, R., Stiller, C.A., Gatta, G., Clavel, J., Stefan, D.C., Carreira, H., Spika, D., Marcos-Gragera, R., Peris-Bonet, R., et al. (2017). Worldwide comparison of survival from childhood leukaemia for 1995–2009, by subtype, age, and sex (CONCORD-2): a population-based study of individual data for 89 828 children from 198 registries in 53 countries. Lancet. Haematol. *4*, e202–e217.

3. Rasche, M., Zimmermann, M., Borschel, L., Bourquin, J.-P., Dworzak, M., Klingebiel, T., Lehrnbecher, T., Creutzig, U., Klusmann, J.-H., and Reinhardt, D. (2018). Successes and challenges in the treatment of pediatric acute myeloid leukemia: a retrospective analysis of the AML-BFM trials from 1987 to 2012. Leukemia *32*, 2167–2177. https://doi.org/10.1038/s41375-018-0071-7.

4. Tierens, A., Arad-Cohen, N., Cheuk, D., De Moerloose, B., Fernandez Navarro, J.M., Hasle, H., Jahnukainen, K., Juul-Dam, K.L., Kaspers, G., and Kovalova, Z. (2024). Mitoxantrone Versus Liposomal Daunorubicin in Induction of Pediatric AML With Risk Stratification Based on Flow Cytometry Measurement of Residual Disease. J Clin Oncol *23*, 01841.

5. Zwaan, C.M., Kolb, E.A., Reinhardt, D., Abrahamsson, J., Adachi, S., Aplenc, R., De Bont, E.S.J.M., De Moerloose, B., Dworzak, M., Gibson, B.E.S., et al. (2015). Collaborative efforts driving progress in pediatric acute myeloid leukemia. J. Clin. Oncol. *33*, 2949–2962. https://doi.org/10.1200/JCO.2015.62.8289.

6. Aplenc, R., Meshinchi, S., Sung, L., Alonzo, T., Choi, J., Fisher, B., Gerbing, R., Hirsch, B., Horton, T., Kahwash, S., et al. (2020). Bortezomib with standard chemotherapy for children with acute myeloid leukemia does not improve treatment outcomes: a report from the Children's Oncology Group. Haematologica *105*, 1879–1886.

7. McNeer, N.A., Philip, J., Geiger, H., Ries, R.E., Lavallée, V.-P., Walsh, M., Shah, M., Arora, K., Emde, A.-K., Robine, N., et al. (2019). Genetic mechanisms of primary chemotherapy resistance in pediatric acute myeloid leukemia. Leukemia *33*, 1934–1943. https://doi.org/10.1038/s41375-019-0402-3.

8. Umeda, M., Ma, J., Westover, T., Ni, Y., Song, G., Maciaszek, J.L., Rusch, M., Rahbarinia, D., Foy, S., Huang, B.J., et al. (2024). A new genomic framework to categorize pediatric acute myeloid leukemia. Nat. Genet. *56*, 281–293.

9. Lambo, S., Trinh, D.L., Ries, R.E., Jin, D., Setiadi, A., Ng, M., Leblanc, V.G., Loken, M.R., Brodersen, L.E., Dai, F., et al. (2023). A longitudinal single-cell atlas of treatment response in pediatric AML. Cancer Cell *41*, 2117–2135.e12.

10. Heikamp, E.B., Henrich, J.A., Perner, F., Wong, E.M., Hatton, C., Wen, Y., Barwe, S.P., Gopalakrishnapillai, A., Xu, H., Uckelmann, H.J., et al. (2022). The menin-MLL1 interaction is a molecular dependency in NUP98-rearranged AML. Blood *139*, 894–906.

11. Aubrey, B.J., Cutler, J.A., Bourgeois, W., Donovan, K.A., Gu, S., Hatton, C., Perlee, S., Perner, F., Rahnamoun, H., Theall, A.C.P., et al. (2022). IKAROS and MENIN coordinate therapeutically actionable leukemogenic gene expression in MLL-r acute myeloid leukemia. Nat. Cancer *3*, 595–613.

12. Farrar, J.E., Schuback, H.L., Ries, R.E., Wai, D., Hampton, O.A., Trevino, L.R., Alonzo, T.A., Guidry Auvil, J.M., Davidsen, T.M., Gesuwan, P., et al. (2016). Genomic profiling of pediatric acute myeloid leukemia reveals a changing mutational landscape from disease diagnosis to relapse. Cancer Res. *76*, 2197–2205.

13. Masetti, R., Castelli, I., Astolfi, A., Bertuccio, S.N., Indio, V., Togni, M., Belotti, T., Serravalle, S., Tarantino, G., Zecca, M., et al. (2016). Genomic complexity and dynamics of clonal evolution in childhood acute myeloid leukemia studied with whole-exome sequencing. Oncotarget *7*, 56746–56757.

14. Rocha, J.C.C., Cheng, C., Liu, W., Kishi, S., Das, S., Cook, E.H., Sandlund, J.T., Rubnitz, J., Ribeiro, R., Campana, D., et al. (2005). Pharmacogenetics of outcome in children with acute lymphoblastic leukemia. Blood *105*, 4752–4758.

15. Relling, M.V., and Evans, W.E. (2015). Pharmacogenomics in the clinic. Nature *526*, 343–350.

16. Elsayed, A.H., Cao, X., Mitra, A.K., Wu, H., Raimondi, S., Cogle, C., Al-Mansour, Z., Ribeiro, R.C., Gamis, A., Kolb, E.A., et al. (2022). Polygenic Ara-C response score identifies pediatric patients with acute myeloid leukemia in need of chemotherapy augmentation. J. Clin. Oncol. *40*, 772–783.

17. Amaki, J., Onizuka, M., Ohmachi, K., Aoyama, Y., Hara, R., Ichiki, A., Kawai, H., Sato, A., Miyamoto, M., Toyosaki, M., et al. (2015). Single nucleotide polymorphisms of cytarabine metabolic genes influence clinical outcome in acute myeloid leukemia patients receiving high-dose cytarabine therapy. Int. J. Hematol. *101*, 543–553.

18. Wachter, F., and Pikman, Y. (2024). Pathophysiology of acute myeloid leukemia. Acta Haematol. *147*, 229–246.

19. Sahoo, S.S., Kozyra, E.J., and Wlodarski, M.W. (2020). Germline predisposition in myeloid neoplasms: Unique genetic and clinical features of GATA2 deficiency and SAMD9/SAMD9L syndromes. Best Pract. Res. Clin. Haematol. *33*, 101197.

20. Hirabayashi, S., Wlodarski, M.W., Kozyra, E., and Niemeyer, C.M. (2017). Heterogeneity of GATA2-related myeloid neoplasms. Int. J. Hematol. *106*, 175–182.

21. Rasche, M., Zimmermann, M., Steidel, E., Alonzo, T., Aplenc, R., Bourquin, J.-P., Boztug, H., Cooper, T., Gamis, A.S., Gerbing, R.B., et al. (2021). Survival following relapse in children with acute myeloid leukemia: a report from AML-BFM and COG. Cancers (Basel) *13*, 2336.

22. Tierens, A., Bjørklund, E., Siitonen, S., Marquart, H.V., Wulff-Juergensen, G., Pelliniemi, T.T., Forestier, E., Hasle, H., Jahnukainen, K., Lausen, B., et al. (2016). Residual disease detected by flow cytometry is an independent predictor of survival in childhood acute myeloid leukaemia; results of the NOPHO-AML 2004 study. Br. J. Haematol. *174*, 600–609.

23. Brodersen, L.E., Gerbing, R.B., Pardo, M.L., Alonzo, T.A., Paine, D., Fritschle, W., Hsu, F.-C., Pollard, J.A., Aplenc, R., Kahwash, S.B., et al. (2020). Morphologic remission status is limited compared to ΔN flow cytometry: a Children's Oncology Group AAML0531 report. Blood Adv. *4*, 5050–5061.

24. Buldini, B., Rizzati, F., Masetti, R., Fagioli, F., Menna, G., Micalizzi, C., Putti, M.C., Rizzari, C., Santoro, N., Zecca, M., et al. (2017). Prognostic significance of flow-cytometry evaluation of minimal residual disease in children with acute myeloid leukaemia treated according to the AIEOP-AML 2002/01 study protocol. Br. J. Haematol. *177*, 116–126.

25. Letai, A. (2017). Functional precision cancer medicine-moving beyond pure genomics. Nat. Med. *23*, 1028–1035. https://doi.org/10.1038/nm.4389.

26. Kornauth, C., Pemovska, T., Vladimer, G.I., Bayer, G., Bergmann, M., Eder, S., Eichner, R., Erl, M., Esterbauer, H., Exner, R., et al. (2022). Functional precision medicine provides clinical benefit in advanced aggressive hematologic cancers and identifies exceptional responders. Cancer Discov. *12*, 372–387. https://doi.org/10.1158/2159-8290.CD-21-0538.

27. Snijder, B., Vladimer, G.I., Krall, N., Miura, K., Schmolke, A.-S., Kornauth, C., Lopez de la Fuente, O., Choi, H.-S., van der Kouwe, E., Gültekin, S., et al. (2017). Image-based ex-vivo drug screening for patients with aggressive haematological malignancies: interim results from a single-arm, open-label, pilot study. Lancet. Haematol. *4*, e595–e606. https://doi.org/10.1016/S2352-3026(17)30208-9.

28. Frismantas, V., Dobay, M.P., Rinaldi, A., Tchinda, J., Dunn, S.H., Kunz, J., Richter-Pechanska, P., Marovca, B., Pail, O., Jenni, S., et al. (2017). Ex vivo drug response profiling detects recurrent sensitivity patterns in drug-resistant acute lymphoblastic leukemia. Blood *129*, e26–e37. https://doi.org/10.1182/blood-2016-09-738070.

29. Peterziel, H., Jamaladdin, N., ElHarouni, D., Gerloff, X.F., Herter, S., Fiesel, P., Berker, Y., Blattner-Johnson, M., Schramm, K., Jones, B.C., et al. (2022). Drug sensitivity profiling of 3D tumor tissue cultures in the pediatric precision oncology program INFORM. NPJ Precis. Oncol. *6*, 94. https://doi.org/10.1038/s41698-022-00335-y.

30. Acanda De La Rocha, A.M., Berlow, N.E., Fader, M., Coats, E.R., Saghira, C., Espinal, P.S., Galano, J., Khatib, Z., Abdella, H., Maher, O.M., et al. (2024). Feasibility of functional precision medicine for guiding treatment of relapsed or refractory pediatric cancers. Nat. Med. *30*, 990–1000.

31. Wang, H., Chan, K.Y.Y., Cheng, C.K., Ng, M.H.L., Lee, P.Y., Cheng, F.W. T., Lam, G.K.S., Chow, T.W., Ha, S.Y., Chiang, A.K.S., et al. (2022). Pharmacogenomic profiling of pediatric acute myeloid leukemia to identify therapeutic vulnerabilities and inform functional precision medicine. Blood Cancer Discov. *3*, 516–535. https://doi.org/10.1158/2643-3230.BCD-22-0011.

32. Strachan, D.C., Gu, C.J., Kita, R., Anderson, E.K., Richardson, M.A., Yam, G., Pimm, G., Roselli, J., Schweickert, A., Terrell, M., et al. (2022). Ex Vivo Drug Sensitivity Correlates with Clinical Response and Supports Personalized Therapy in Pediatric AML. Cancers (Basel) *14*, 6240. https://doi.org/10.3390/cancers14246240.

33. Cucchi, D.G.J., Groen, R.W.J., Janssen, J.J.W.M., and Cloos, J. (2020). Ex vivo cultures and drug testing of primary acute myeloid leukemia samples: Current techniques and implications for experimental design and outcome. Drug Resist. Updat. *53*, 100730.

34. Maurer-Granofszky, M., Köhrer, S., Fischer, S., Schumich, A., Nebral, K., Larghero, P., Meyer, C., Mecklenbräuker, A., Mühlegger, N., Marschalek, R., et al. (2024). Genomic breakpoint-specific monitoring of measurable residual disease in pediatric non-standard-risk acute myeloid leukemia. Haematologica *109*, 740–750.

35. He, K., Gkioxari, G., Dollár, P., and Girshick, R. (2017). Mask R-CNN. Preprint at arXiv. https://doi.org/10.48550/arxiv.1703.06870.

36. Makhzani, A., Shlens, J., Jaitly, N., Goodfellow, I., and Frey, B. (2015). Adversarial Autoencoders. Preprint at arXiv. https://doi.org/10.48550/arxiv.1511.05644.

37. Kropivsek, K., Kachel, P., Goetze, S., Wegmann, R., Festl, Y., Severin, Y., Hale, B.D., Mena, J., van Drogen, A., Dietliker, N., et al. (2023). Ex vivo drug response heterogeneity reveals personalized therapeutic strategies for patients with multiple myeloma. Nat. Cancer *4*, 734–753.

38. Dietrich, S., Oleś, M., Lu, J., Sellner, L., Anders, S., Velten, B., Wu, B., Hüllein, J., da Silva Liberio, M., Walther, T., et al. (2018). Drug-perturbation-based stratification of blood cancer. J. Clin. Investig. *128*, 427–445. https://doi.org/10.1172/JCI93801.

39. Stieglitz, E., Gu, C.J., Richardson, M., Kita, R., Santaguida, M.T., Ali, K.A., Strachan, D.C., Dhar, A., Yam, G., Anderson, W., et al. (2023). Tretinoin Enhances the Effects of Chemotherapy in Juvenile Myelomonocytic Leukemia Using an Ex Vivo Drug Sensitivity Assay. JCO Precis. Oncol. *7*, e2300302.

40. Kuusanmäki, H., Leppä, A.-M., Pölönen, P., Kontro, M., Dufva, O., Deb, D., Yadav, B., Brück, O., Kumar, A., and Everaus, H. (2019). Phenotype-based drug screening reveals association between venetoclax response and differentiation stage in acute myeloid leukemia. Haematologica *105*, 708.

41. Spinner, M.A., Aleshin, A., Santaguida, M.T., Schaffert, S.A., Zehnder, J. L., Patterson, A.S., Gekas, C., Heiser, D., and Greenberg, P.L. (2020). Ex vivo drug screening defines novel drug sensitivity patterns for informing personalized therapy in myeloid neoplasms. Blood Adv. *4*, 2768–2778.

42. Vo, T.-T., Ryan, J., Carrasco, R., Neuberg, D., Rossi, D.J., Stone, R.M., DeAngelo, D.J., Frattini, M.G., and Letai, A. (2012). Relative mitochondrial priming of myeloblasts and normal HSCs determines chemotherapeutic success in AML. Cell *151*, 344–355.

43. Konopleva, M., Pollyea, D.A., Potluri, J., Chyla, B., Hogdal, L., Busman, T., McKeegan, E., Salem, A.H., Zhu, M., Ricker, J.L., et al. (2016). Efficacy and biological correlates of response in a phase II study of venetoclax monotherapy in patients with acute myelogenous leukemia. Cancer Discov. *6*, 1106–1117.

44. Corces, M.R., Buenrostro, J.D., Wu, B., Greenside, P.G., Chan, S.M., Koenig, J.L., Snyder, M.P., Pritchard, J.K., Kundaje, A., Greenleaf, W.J., et al. (2016). Lineage-specific and single-cell chromatin accessibility charts human hematopoiesis and leukemia evolution. Nat. Genet. *48*, 1193–1203.

45. Schep, A.N., Wu, B., Buenrostro, J.D., and Greenleaf, W.J. (2017). chromVAR: inferring transcription-factor-associated accessibility from single-cell epigenomic data. Nat. Methods *14*, 975–978.

46. Zaiss, D.M.W., and Coffer, P.J. (2018). Forkhead box transcription factors as context-dependent regulators of lymphocyte homeostasis. Nat. Rev. Immunol. *18*, 703–715.

47. Sango, J., Carcamo, S., Sirenko, M., Maiti, A., Mansour, H., Ulukaya, G., Tomalin, L.E., Cruz-Rodriguez, N., Wang, T., Olszewska, M., et al. (2024). RAS-mutant leukaemia stem cells drive clinical resistance to venetoclax. Nature *636*, 241–250.

48. Pei, S., Pollyea, D.A., Gustafson, A., Stevens, B.M., Minhajuddin, M., Fu, R., Riemondy, K.A., Gillen, A.E., Sheridan, R.M., Kim, J., et al. (2020). Monocytic subclones confer resistance to venetoclax-based therapy in patients with acute myeloid leukemia. Cancer Discov. *10*, 536–551.

49. Placke, T., Faber, K., Nonami, A., Putwain, S.L., Salih, H.R., Heidel, F.H., Krämer, A., Root, D.E., Barbie, D.A., Krivtsov, A.V., et al. (2014). Requirement for CDK6 in MLL-rearranged acute myeloid leukemia. Blood *124*, 13–23.

50. Schmoellerl, J., Barbosa, I.A.M., Eder, T., Brandstoetter, T., Schmidt, L., Maurer, B., Troester, S., Pham, H.T.T., Sagarajit, M., Ebner, J., et al. (2020). CDK6 is an essential direct target of NUP98 fusion proteins in acute myeloid leukemia. Blood *136*, 387–400.

51. Ni Chonghaile, T., Sarosiek, K.A., Vo, T.-T., Ryan, J.A., Tammareddi, A., Moore, V.D.G., Deng, J., Anderson, K.C., Richardson, P., Tai, Y.-T., et al. (2011). Pretreatment mitochondrial priming correlates with clinical response to cytotoxic chemotherapy. Science *334*, 1129–1133.

52. Torkamani, A., Andersen, K.G., Steinhubl, S.R., and Topol, E.J. (2017). High-Definition Medicine. Cell *170*, 828–843. https://doi.org/10.1016/j.cell.2017.08.007.

53. Letai, A. (2022). Precision medicine in AML: Function plus-omics is better than either alone. Cancer Cell *40*, 804–806.

54. Malani, D., Kumar, A., Brück, O., Kontro, M., Yadav, B., Hellesøy, M., Kuusanmäki, H., Dufva, O., Kankainen, M., Eldfors, S., et al. (2022). Implementing a functional precision medicine tumor board for acute myeloid leukemia. Cancer Discov. *12*, 388–401.

55. Schumich, A., Prchal-Murphy, M., Maurer-Granofszky, M., Hoelbl-Kovacic, A., Mühlegger, N., Pötschger, U., Fajmann, S., Haas, O.A., Nebral, K., von Neuhoff, N., et al. (2020). Phospho-Profiling Linking Biology and Clinics in Pediatric Acute Myeloid Leukemia. Hemasphere *4*, e312. https://doi.org/10.1097/HS9.0000000000000312.

56. Arber, D.A., Orazi, A., Hasserjian, R.P., Borowitz, M.J., Calvo, K.R., Kvasnicka, H.-M., Wang, S.A., Bagg, A., Barbui, T., Branford, S., et al. (2022). International Consensus Classification of Myeloid Neoplasms and Acute Leukemias: integrating morphologic, clinical, and genomic data. Blood *140*, 1200–1228.

57. Tyner, J.W., Tognon, C.E., Bottomly, D., Wilmot, B., Kurtz, S.E., Savage, S. L., Long, N., Schultz, A.R., Traer, E., Abel, M., et al. (2018). Functional genomic landscape of acute myeloid leukaemia. Nature *562*, 526–531. https://doi.org/10.1038/s41586-018-0623-z.

58. Bottomly, D., Long, N., Schultz, A.R., Kurtz, S.E., Tognon, C.E., Johnson, K., Abel, M., Agarwal, A., Avaylon, S., Benton, E., et al. (2022). Integrative analysis of drug response and clinical outcome in acute myeloid leukemia. Cancer Cell *40*, 850–864.e9.

59. Lee, S.H.R., Yang, W., Gocho, Y., John, A., Rowland, L., Smart, B., Williams, H., Maxwell, D., Hunt, J., Yang, W., et al. (2023). Pharmacotypes across the genomic landscape of pediatric acute lymphoblastic leukemia and impact on treatment response. Nat. Med. *29*, 170–179. https://doi.org/10.1038/s41591-022-02112-7.

60. Huang, B.J., Smith, J.L., Farrar, J.E., Wang, Y.-C., Umeda, M., Ries, R.E., Leonti, A.R., Crowgey, E., Furlan, S.N., Tarlock, K., et al. (2022). Integrated stem cell signature and cytomolecular risk determination in pediatric acute myeloid leukemia. Nat. Commun. *13*, 5487.

61. Gentles, A.J., Plevritis, S.K., Majeti, R., and Alizadeh, A.A. (2010). Association of a leukemic stem cell gene expression signature with clinical outcomes in acute myeloid leukemia. JAMA *304*, 2706–2715.

62. Duployez, N., Marceau-Renaut, A., Villenet, C., Petit, A., Rousseau, A., Ng, S.W.K., Paquet, A., Gonzales, F., Barthélémy, A., Leprêtre, F., et al. (2019). The stem cell-associated gene expression signature allows risk stratification in pediatric acute myeloid leukemia. Leukemia *33*, 348–357.

63. Pollyea, D.A., and Jordan, C.T. (2017). Therapeutic targeting of acute myeloid leukemia stem cells. Blood *129*, 1627–1635.

64. Van Tilburg, C.M., Milde, T., Witt, R., Ecker, J., Hielscher, T., Seitz, A., Schenk, J.-P., Buhl, J.L., Riehl, D., Frühwald, M.C., et al. (2019). Phase I/II intra-patient dose escalation study of vorinostat in children with relapsed solid tumor, lymphoma, or leukemia. Clin. Epigenetics *11*, 188.

65. Goldberg, J., Sulis, M.L., Bender, J., Jeha, S., Gardner, R., Pollard, J., Aquino, V., Laetsch, T., Winick, N., Fu, C., et al. (2020). A phase I study of panobinostat in children with relapsed and refractory hematologic malignancies. Pediatr. Hematol. Oncol. *37*, 465–474.

66. Letai, A., Bhola, P., and Welm, A.L. (2022). Functional precision oncology: testing tumors with drugs to identify vulnerabilities and novel combinations. Cancer Cell *40*, 26–35.

67. Irmisch, A., Bonilla, X., Chevrier, S., Lehmann, K.-V., Singer, F., Toussaint, N.C., Esposito, C., Mena, J., Milani, E.S., Casanova, R., et al. (2021). The Tumor Profiler Study: integrated, multi-omic, functional tumor profiling for clinical decision support. Cancer Cell *39*, 288–293.

68. Casteels, T., Bajew, S., Reiniš, J., Enders, L., Schuster, M., Fontaine, F., Müller, A.C., Wagner, B.K., Bock, C., and Kubicek, S. (2022). SMNDC1 links chromatin remodeling and splicing to regulate pancreatic hormone expression. Cell Rep. *40*.

69. Abdulla, W. Mask R-CNN for object detection and instance segmentation on Keras and TensorFlow. https://github.com/matterport/Mask_RCNN.

70. Kromp, F., Bozsaky, E., Rifatbegovic, F., Fischer, L., Ambros, M., Berneder, M., Weiss, T., Lazic, D., Dörr, W., Hanbury, A., and Beiske, K. (2020). An annotated fluorescence image dataset for training nuclear segmentation methods. Sci. Data *7*, 262.

71. Gunesli, G.N., Sokmensuer, C., and Gunduz-Demir, C. (2020). AttentionBoost: Learning what to attend for gland segmentation in histopathological images by boosting fully convolutional networks. IEEE Trans. Med. Imaging *39*, 4262–4273.

72. Korfhage, N., Muehling, M., Ringshandl, S., Becker, A., Schmeck, B., and Freisleben, B. (2020). Detection and segmentation of morphologically complex eukaryotic cells in fluorescence microscopy images via feature pyramid fusion. PLoS Comput. Biol. *16*, e1008179.

73. Caicedo, J.C., Goodman, A., Karhohs, K.W., Cimini, B.A., Ackerman, J., Haghighi, M., Heng, C., Becker, T., Doan, M., McQuin, C., and Rohban, M. (2019). Nucleus segmentation across imaging experiments: the 2018 Data Science Bowl. Nat. Methods *16*, 1247–1253.

74. Reichl, S., Ergüner, B., Barreca, D., Folkman, L., Dobnikar, L., and Bock, C. (2024). Ultimate ATAC-Seq Data Processing & Quantification Pipeline (v1.1.0) (Zenodo). https://doi.org/10.5281/zenodo.11087225.

75. Auwera, G.A., and O'Connor, B.D. (2020). Genomics in the cloud: using Docker, GATK, and (WDL in Terra).

76. Kim, S., Scheffler, K., Halpern, A.L., Bekritsky, M.A., Noh, E., Källberg, M., Chen, X., Kim, Y., Beyter, D., Krusche, P., and Saunders, C.T. (2018). Strelka2: fast and accurate calling of germline and somatic variants. Nat. Methods *15*, 591–594.

77. Chen, X., Schulz-Trieglaff, O., Shaw, R., Barnes, B., Schlesinger, F., Källberg, M., Cox, A.J., Kruglyak, S., and Saunders, C.T. (2016). Manta: rapid detection of structural variants and indels for germline and cancer sequencing applications. Bioinformatics *32*, 1220–1222.

STAR★METHODS

KEY RESOURCES TABLE

REAGENT or RESOURCE	SOURCE	IDENTIFIER
Antibodies		
CD117-PE	BD Biosciences	Cat# 555714; RRID:AB_396058
CD34-APC	BD Biosciences	Cat# 555714; RRID:AB_396058
CD3-FITC	BD Biosciences	Cat# 345763; RRID:AB_2811220
CD3-APC	BD Biosciences	Cat# 345767; RRID:AB_2833003
CD11a-APC	BD Biosciences	Cat# 550852; RRID:AB_398466
CD33-PE	BioLegend	Cat# 366608; RRID:AB_2566107
HLA-DR-FITC	BioLegend	Cat# 307604; RRID:AB_314682
CD13-PE	EXBIO Praha	Cat# 1P-396-T100; RRID:AB_10736238
Critical commercial assays		
CellTiter-Glo	Promega	Cat# G7573
Qiagen AllPrep DNA/RNA mini kit	Qiagen	Cat# 80204
Twist Library Preparation EF Kit	Twist Biosciences	Cat# 104207
Tagment DNA TDE1 Enzyme and Buffer Kits	Illumina	Cat# 20034198
Deposited data		
Healthy reference ATAC-seq data from Corces et al.	Gene Expression Omnibus	GEO: GSE74912
ATAC-seq data from this study	Gene Expression Omnibus	GEO: GSE282258
RNA-seq data from this study	Gene Expression Omnibus	GEO: GSE282423
Exome sequencing data from this study	European Genome Phenome Archive	EGA: EGAD50000001572
Software and algorithms		
Code repository for main analyses in this study	GitHub	DOI: https://doi.org/10.5281/zenodo.16368448
Code repository for image-based screening pipeline	GitHub	DOI: https://doi.org/10.5281/zenodo.16330001
Code repository for models to predict cell types and cell viabilities	GitHub	DOI: https://doi.org/10.5281/zenodo.16330095
Other		
SpectraMax i3 plate reader	Molecular Devices	N/A
Opera Phenix High Content Screening System	Perkin Elmer	RRID:SCR_021100

EXPERIMENTAL MODEL AND STUDY PARTICIPANT DETAILS

Fresh-frozen samples of primary bone-marrow mononuclear cells (MNCs) were acquired from the biobank of the St. Anna Children's Hospital Vienna, Austria covering a total of 45 patients. All patients or their respective legal guardians gave written informed consent prior to the study. Samples are standardized to contain 10 million MNCs per vial. Fresh samples for testing and optimization of 5 additional patients aged 0–18 years were taken from leftover material that was obtained during routine diagnostic procedures. Of these, one sample with the ID fAML01 was used for assessing correlations between Image-based viability assessment and CellTiterGlo, two samples with the IDs fAML02 and fAML03 were additionally used for viability model training and two samples with the IDs pAML01 and pAML02 were additionally used to train the cell type model. These samples were only used for the technical benchmarking in this study and thus not subjected to further characterization. All samples were acquired under the appropriate ethics approval by the independent ethics committee of the Medical University of Vienna (institutional review board vote No.1500/2014). Patients were aged 0–18 years at the time of sample acquisition, selected in a gender- and sex-balanced fashion (n = 20 female, n = 25 male) and are of European descent. More information on individual samples can be found in Table S2.

METHOD DETAILS

Drug treatment

Screening plates were acquired from the Molecular Discovery Platform of the Center for Molecular Medicine of the Austrian Academy of Sciences (CeMM) and stored at −80°C until screening.

Every sample was screened on two screening plates spotted with a total of 115 compounds in 3 concentrations and duplicates. All compounds were dissolved in DMSO. Additionally, each plate contains 32 control wells spotted with DMSO. Compounds were spotted onto plates in a randomized layout. Plates were thawed in the dark for 3 h before screening.

Bone-marrow mononuclear cells (MNCs) were either thawed from frozen vials or isolated from fresh bone marrow via Ficoll gradient separation. Subsequently, cells were resuspended in IMDM with 10% FCS and 1% Penicillin and Streptamycin and seeded onto plates at a density of 9.000 cells per well and incubated at 37°C with 5% CO2 for 24 h.

Additional drug treatment for benchmarking of Pharmacoscopy and CellTiterGlo (Figure 1G) was performed using the compounds described in the respective figure in quadruplicates at concentrations of 10 nM, 100 nM, 1 μM and 10 μM.

Staining and image acquisition

Treatment incubation was stopped by adding 15 μL of fixation-permeabilization buffer (PBS with 0.5% Formaldehyde, 0.1% X-114), incubating for 15 min and subsequently washing three times with PBS. Then, 15 μL of PBS were added to all wells. Subsequently, cells were stained with sample specific staining solution containing 10 mM solutions of DAPI and Hoechst in a concentration of 1:1500 each, leading to a total concentration of 1:750, and the appropriate antibody cocktail to among the following antibodies: CD117-PE (BD Biosciences Cat# 555714, RRID:AB_396058), CD34-APC (BD Biosciences Cat# 345804, RRID:AB_2686894), CD3-FITC (BD Biosciences Cat# 345763, RRID:AB_2811220), CD3-APC (BD Biosciences Cat# 345767, RRID:AB_2833003) CD11a-APC (BD Biosciences Cat# 550852, RRID:AB_398466), CD33-PE (BioLegend Cat# 366608, RRID:AB_2566107), HLA-DR-FITC (BioLegend Cat# 307604, RRID:AB_314682), CD13-PE (EXBIO Praha Cat# 1P-396-T100, RRID:AB_10736238). The antibodies for CD3 and CD13 were added in a concentration of 1:150. CD11a was added in a concentration of 1:200. All other antibodies were added in a concentration of 1:250. 15 μL of staining solution were added to each well. The first plate was incubated for 6 h at room temperature and the second one overnight at 4°C until imaging with 4 h at RT before imaging for the second plate. Images were acquired with a PerkinElmer Opera Phenix microscope at 20× resolution, leading to 25 images at a resolution of 1080x1080 pixels, covering the full well for each well and each channel.

Viability staining was performed with Live-or-Dye fixable viability staining kit (Biotium, San Francisco, CA, USA) according to the manufacturer's recommendations. In brief, cells were washed 2× with PBS. Subsequently, 15 μL of viability dye solution at a concentration of 1:300 was added to each well. After 30 min of incubation away from light, cells were prepared for imaging as described in before in this paragraph.

Image analysis

After image acquisition and export, images were analyzed with CellProfiler (v4.2.1, RRID:SCR_007358).[68] We added custom modules for nuclear segmentation - described further below - and gamma correction with corresponding function from the scikit-image library (v0.18.1). CellProfiler outputs were written to sqlite databases for further analyses. Subsequently, we performed quality control by first excluding images that were likely to be improperly stained by excluding wells with average intensities at least 4 standard deviations away from the average well intensity for each imaged channel. Furthermore, we excluded artifacts and improperly segmented cells by thresholding on morphological descriptors for intensity, texture and cell-shape. After this QC step, we applied our AAE models for cell type and viability prediction as described in the following section. Our pipeline then enumerated all viable cells for each marker and wrote them into a single table for further analysis. More details can be found in the code repository referenced in the data and code availability section.

Nucleus segmentation and segmentation model training

We adapted a Mask-R-CNN implementation[35,69] with pre-trained ImageNet weights for nucleus segmentation and compiled a custom training dataset from 4 different sources[70–73], as well as a small selection of images that were annotated in-house. The training dataset comprises a total of 1523 images and 43519 nuclei. We randomly split the dataset into 80% training images and 20% testing images, subsequently trained the model for 100 epochs and picked the weights from the epoch with the lowest combined loss value on the testing dataset as our final model for further segmentation tasks.

Cell type prediction

To train a predictive model to classify cells by their marker expression profile, we generated an in-house dataset comprising of 16553 cells imaged over 550 fields of view from 11 samples. Samples were selected to cover all cell surface markers that were stained across the full cohort. We then randomly sampled 50 image patches with sizes of 500x500 pixels per plate and manually annotated cells as positive or negative for the given markers using the VGG image annotator. Subsequently, we trained an adversarial autoencoder (AAE) with hyperparameter optimization to derive a model that predicts marker positivity from image data. Our model receives two-channel patches with a size of 32x32 pixels as input that contain the DAPI channel and the current channel of interest. Models were trained with tensorflow (v2.11.0) and keras (v2.11.0). Model hyperparameter optimization was performed with keras-tuner (v1.1.0).

Viability prediction

We performed automated labeling of nuclei for semi-supervised viability learning based on morphology and viability dye intensity jointly. Assuming that distinct cell-subpopulations have different protein contents, and will therefore have different viability dye

intensities, we first performed UMAP dimensionality reduction of nuclei morphology features, followed by unsupervised clustering of nuclei in the reduced feature space using HDBSCAN (v0.8.38.post2). Subsequently, we fit Gaussian mixture models comprised of two independent Gaussian distributions on the viability dye intensities for each cluster. To finally derive high-confidence labels of cell viability, we performed two additional filtering steps. First, we only assigned labels to cells that were at least 3 standard deviations away from the respective positive or negative distribution respectively. Then, we fit a K nearest neighbors (KNN) model on the data using the distribution fits as labels, and only kept labels were distribution fits and KNN clusters agreed with each other. The model was then trained on 1000 randomly sampled fields of view from 5 samples, yielding roughly 1.8 million cells with a labeling rate of 29.5%.

Hyperparameter optimization for adversarial autoencoders

We performed hyperparameter optimization using keras-tuner (v1.1.0) with Bayesian optimization on patches of a size 32x32 pixels for an adversarial autoencoder with a set of convolutional layers, followed by dense layers with 500 neurons and used the following hyperparameter ranges: Number of dense layers: 2,3; z-dimensionality:25,100,500; Number of filters per convolutional layer: [16, 128, 16, 128, 16], [128, 16, 128, 16, 128], [128, 128, 64, 64, 16], [128, 128, 64, 64, 16, 16], [128, 64, 128, 16, 64, 16], [16, 16, 64, 64, 128], [16, 16, 64, 64, 128, 128], [16, 16, 128, 128, 128]; class learning rate: 0.0001, 0.00001; Optimizer: Adam, Nadam; Augmentation: Flip only, flip and random brightness changes. We additionally set a dropout rate of 0.2. We subsequently selected the architecture with the lowest classification loss. The final architecture for cell type prediction had a z-dimensionality of 100, a learning rate of 10^{-4} a filter size of 3, 3 dense layers, used the Nadam optimizer, had [16,128,16,128,16] filters and was trained with flipping only. The final architecture for viability prediction had a z dimensionality of 25, a learning rate of 10^{-4} a filter size of 5, 2 dense layers, used the Adam optimizer, had [16, 16, 128, 128, 128] filters and was trained with flipping only.

Benchmarking of image-based screening and CellTiterGlo data

Cells were incubated in compound concentrations ranging from 10 nM to 10 μM in quadruplicates for 24 h in different densities ranging from 9000 cells per well to 2250 cells per well. Treatment was performed in two 384 well plates per experiment. After the incubation period, CellTiterGlo (Promega) was performed according to the manufacturer's protocol. Fluorescent viability readouts were measured on a plate-reader (SpectraMax i3, Molecular Probes). Image-based screening was performed in parallel as described above.

Processing of dose response values and compound activity scoring

Dose response values were first filtered for outliers by filtering wells with total cell numbers that were more than 4 standard deviations higher than the average number in the DMSO control. Subsequently, we filtered out outlier replicate values, by filtering out values that were 50% above the average value for the previous concentration point. We additionally filtered out concentrations with more than a 50% difference between replicates.

We scored compound activity using an approximate area under the curve (AUC) metric. First, we used linear interpolation to map all dose response values to the same concentration range. Then, we calculated relative blast fractions (RBFs) for each treated condition as described previously,[26,27] by dividing the viable blast fraction in treated wells by the mean viable blast fraction in negative control wells. Subsequently we calculated the approximate AUC by integrating over the average RBFs per concentration point and calculated the final RBF-AUC inhibition score as 1 - AUC, such that higher values indicate stronger inhibitions. We fit dose-response curves as shown in Figure 3 using a 3-parameter dose response model and normalized absolute blast fractions to the average of the negative DMSO control. RBF-AUC values were scaled by subtracting the mean and dividing by the standard deviation for cohort-level depictions such as Figures 4, 5, and 6. Absolute response values - denoted as Blast AUC as shown in Figure S4B - were calculated analogously, but instead of dividing the viable fraction of blasts in treated wells by the mean viable blast fraction in negative control wells, we divided the absolute number of viable blasts in treated wells, by the mean absolute number of viable blasts in negative control wells, thus ignoring non-malignant populations.

Drug-drug correlation analysis

To derive the most robust drug-drug pairs for subsequent correlation analysis, we first filtered for drugs with at least approximately 50% inhibition in at least 3 samples, by only keeping drugs with a Blast AUC value of 0.25 or higher in at least 3 samples. This analysis led to 58 of 115 drugs or combinations being kept. We quantified strength of correlation with Spearman correlation coefficients for each drug pair. For the analysis of correlation by drug-class, we manually annotated drugs to their respective classes (Table S2) and subsequently kept all pairs of classes where all drug-pairs between the two classes have either positive or negative Spearman Correlation coefficients.

Immunophenotyping for identification of markers for leukemic blasts

Immunophenotyping has been performed at diagnosis on erythrocyte-lysed whole bone marrow (BM) samples using multiparameter flow cytometry (MFC). As in our previous work, we used a 10-color format cocktail across two tubes[34]: Tube 1 included HLA-DR, CD45, CD15, CD34, CD117, CD33, CD14, and CD11b, while Tube 2 contained HLA-DR, CD45, CD38, CD371, CD34, CD117,

CD33, CD99, CD123, and CD45RA. Patient-specific LAIP antigens, such as lymphoid markers (e.g., CD2, CD4, CD7, CD19, CD56) or other aberrant markers (e.g., CD11a, CD13, CD71, CD99, NG2), were included as "drop-ins" only in tube 1.

FACS staining for cell sorting

Cells were washed using PBS with 2% FBS. After spinning down at 400g for 8 min at either room temperature or 4°C, the supernatant was removed, and the cells were resuspended in 300 μL of PBS/2% FBS. Of this suspension, 250 μL was transferred to a filter-cap FACS tube (Ref. nr. 352235). Patient-specific markers for leukemic blasts together with CD3, CD19 and CD45 were used to sort normal CD3 T cells or CD19 B cells and leukemic blasts. Following a brief vortex and 15-min incubation at room temperature in the dark, the cells were washed once, and the supernatant was removed. The cells were resuspended in PBS/2% FBS/25 mM HEPES and sorted using a FACS Aria (BD Biosciences) cell sorter.

Cell number and cell viability assessment via flow cytometry

For determining absolute cell numbers and viability we used Trucount tubes (BD Biosciences) in conjunction with 7-AAD and CD45 staining as well as with antibodies for identification of leukemic blasts (as identified with immunophenotyping, i.e., CD117, CD34 or CD33) as well as normal T cells (CD3). Measurements were conducted on a FACS Symphony (BD Biosciences) analyzer.

Transcriptome, exome and accessible chromatin sequencing

RNA and DNA from FACS isolated blasts and DNA healthy lymphoid cells were isolated using the Qiagen AllPrep DNA/RNA mini kit (Cat. No 80204) according to the manufacturer's protocol. Amplification of cDNA was performed using the SmartSeq2 protocol. Library construction was performed using the Twist Library Preparation EF Kit (Twist Biosciences). DNA for ATAC-seq was isolated from 50.000 sorted blasts using the Tagment DNA TDE1 Enzyme and Buffer Kits (Illumina, CA, USA) RNA from isolated blasts.

mRNA sequencing data analysis

We merged the unaligned.bam files using Picard v3.0.0 (RRID:SCR_006525) and extracted UMI sequences for each read from "BC" tags (which contained sample index reads and UMIs) into "RX" tags using a custom Python script. We converted the unaligned.bam files to.fastq format using Picard v3.0.0, filtered according to quality and length with fastp v0.23.3, and aligned to the GRCh38 genome reference (Ensembl v102) with STAR v2.7.10. We then sorted the aligned reads using Picard v3.0.0, and transferred the RX tags containing the UMI sequences from the unaligned to the aligned.bam files using GATK v4.4.0. We added read mate information, grouped reads by UMIs, and called molecular consensus sequences using fgbio v2.1.0, then converted the resulting.bam files to.fastq format using SAMtools v1.17 (RRID:SCR_002105) and subsequently quantified read counts using Salmon v1.9.0 in mapping based mode against a salmon index built from the GRCh38 transcriptome.

ATAC-seq analysis

ATAC-seq data was analyzed as described in Casteels et al.[68,74] In brief, sequence adapter trimming and initial filtering were performed with fastp v0.20.1 and alignments to the GRCh38 human reference genome were performed using bowtie2 v2.4.4 with the –very-sensitive parameter. PCR duplicates were marked using Samblaster v0.1.24. Sorting, filtering of ENCODE blacklisted regions and subsequent indexing was performed using samtools v1.12 and peaks were called using MACS2 v2.2.7.1. Only samples with at least 40% of mapped reads, a duplication rate below 50% as detected via Samblaster and fractions of reads in peaks as detected by MACS2 of more than 10% were considered for futher downstream analysis. Identified peak summits were then merged to generate a consensus region set. This analysis was performed for the dataset from this study and the healthy reference dataset from Corces et al.[44] independently, and reads from this study were subsequently quantified for the consensus regions derived from the healthy reference. Subsequent unsupervised analyses were performed on CQN normalized read counts using the CQN package and trimmed mean of M normalized library sizes as provided by the edgeR package. Batch correction was performed using reCombat.

WES analysis

Raw sequencing reads were processed using the nf-core sarek WES pipeline version 2.7.2. Variant calling was conducted in a tumor-normal matched mode, utilizing CD3 or CD19 FACS-sorted cells as the matched normal samples. Three variant callers - Mutect2[75], Strelka[76], and Manta[77] - were employed for comprehensive variant identification. The resulting VCF files from Mutect2 and Strelka (following the best practices workflow) were normalized using bcftools norm (bcftools v1.9) and subsequently annotated with the Ensembl Variant Effect Predictor (VEP) (VEP v99.2). A coordinate-based filtering was conducted using the start and end coordinates of genes listed in an in-house AML gene panel.

Predictions of healthy cell types and clinical variables

Predictive models for mapping to healthy references and for clinical variables were implemented using the scikit-learn (v1.3.2). Random Forest models for clinical variable prediction were initialized with balanced class weights, without bootstrapping and with 101 estimators. Support vector machines for healthy cell type prediction were initialized with radial-basis function kernels and balanced class weights on the 1000 most variable consensus regions.

QUANTIFICATION AND STATISTICAL ANALYSIS

All statistical tests of were performed using scipy (v1.13.0) in Python (v3.11.6). Significance of differences between quantitative measurements was assessed using the Mann-Whitney-U test unless otherwise indicated. Multiple testing correction was performed using Bonferroni correction unless otherwise indicated.

ADDITIONAL RESOURCES

Risk stratification for samples in this study was performed according to the criteria established by the AIEOP-BFM-AML consortium: EUCT: 2022-500783-35-00.

Cell Reports Medicine, Volume *6*

Supplemental information

Image-based drug screening combined with molecular profiling identifies signatures and drivers of therapy resistance in pediatric AML

Ben Haladik, Margarita Maurer-Granofszky, Peter Zoescher, Raul Jimenez-Heredia, Alexandra Frohne, Anna Segarra-Roca, Chloe Casey, Felix Kartnig, Sarah Giuliani, Christina Rashkova, Peter Repiscak, Michael N. Dworzak, Giulio Superti-Furga, and Kaan Boztug

Supplementary Figures

Figure S1

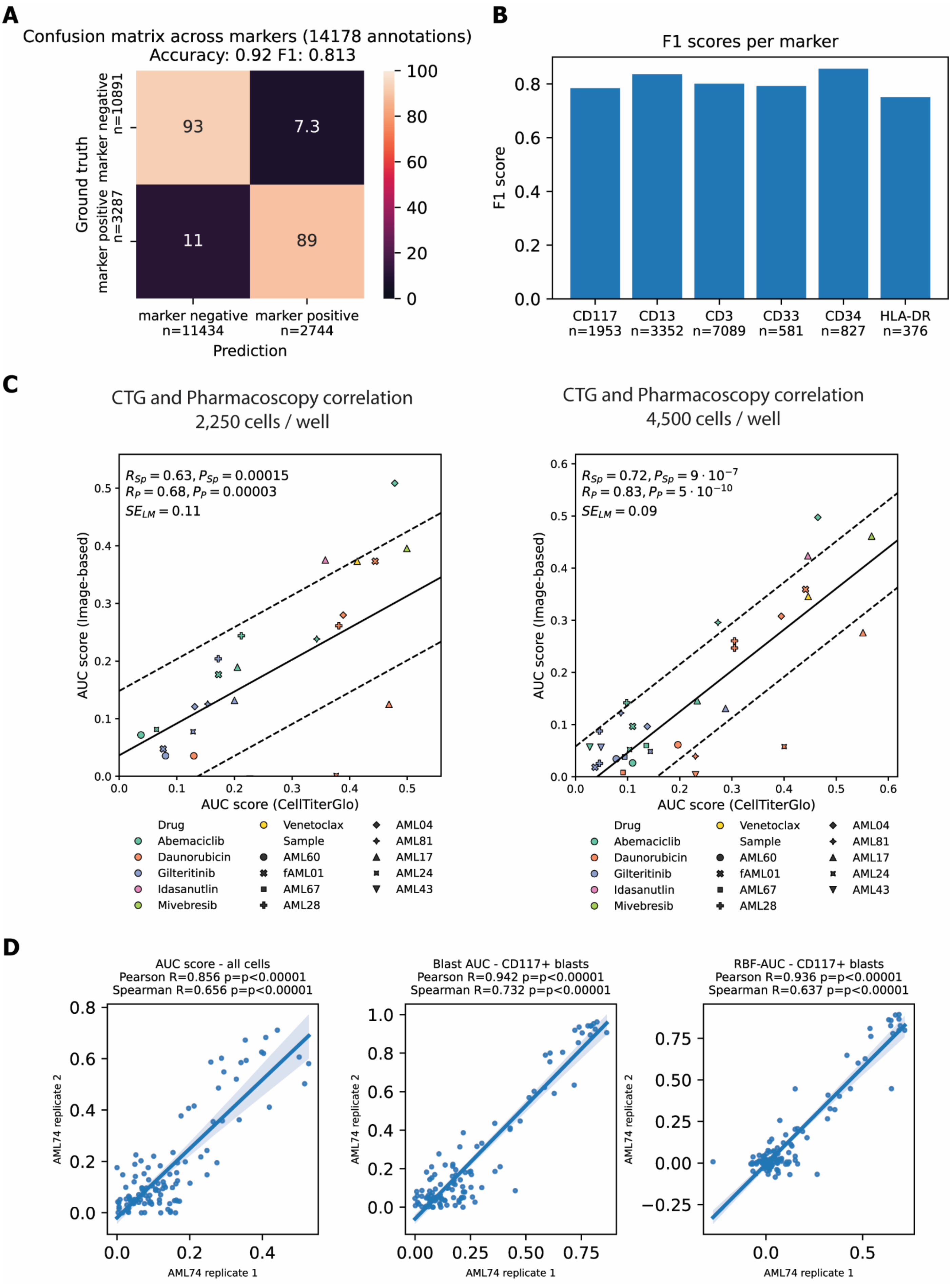

Figure S1: Pharmacoscopy technical benchmarkings. Related to Figure 1

A Confusion matrix for marker-positivity across all cells in the testing dataset for the celltype model. **B** Bargraph of F1 scores per surface antigen in the testing dataset. The bottom number indicates the number of cells **C** Left: Correlation between CTG inhibition scores and Pharmacoscopy inhibition scores for 6 drugs and 9 samples for a cell-density of 2.250 cells per well. Right: same as left, but with 4.500 cells per well. **D** Correlation between replicates across readouts for the AUC score on all cells (left), target cells only (middle) and the RBF-AUC score (right).

Figure S2

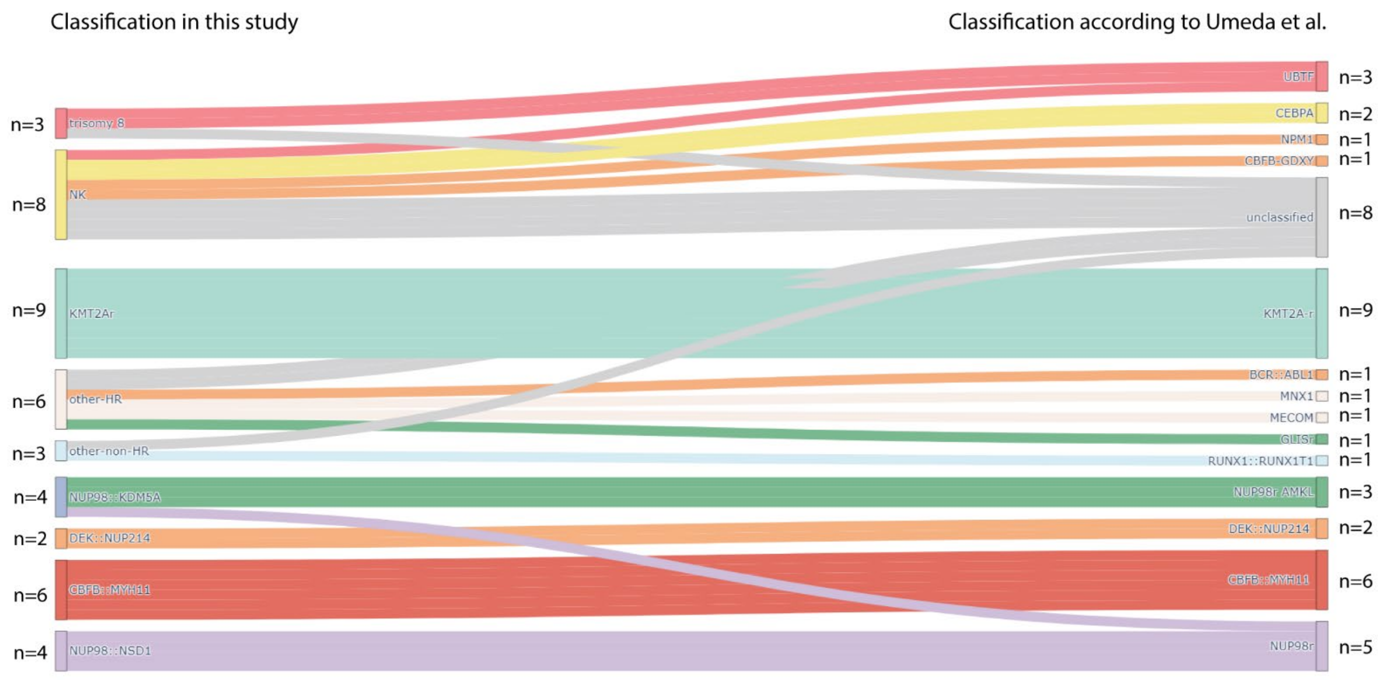

Figure S2: Molecular re-classification of our cohort. Related to Figure 2

Sankey chart indicating matchings between the classification in this study for the n=45 samples in our cohort and the classification in Umeda et al.

Figure S3

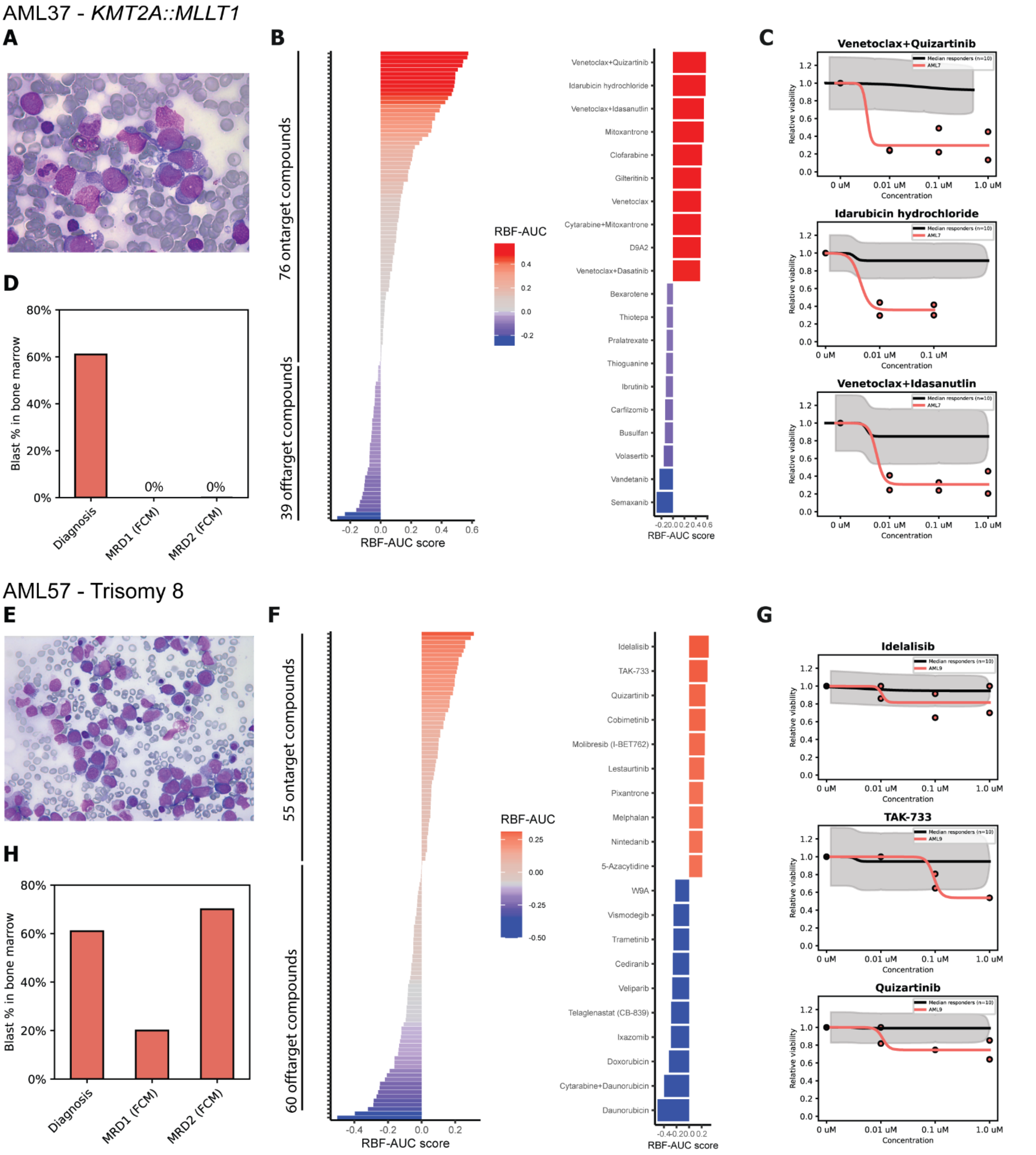

Figure S3: Case vignettes illustrating our drug sensitivity profiling reporting format. Related to Figure 3

A Bone marrow smear of sample AML37 (good responder). The patient had a monoblastic M5a phenotype and a *KMT2A::MLLT1* fusion. **B** Left: Bar chart of RBF-AUC scores for all tested compounds. Right: Bar chart of RBF-AUC scores for the 10 compounds with the highest and lowest RBF-AUC scores respectively and the induction regimen drugs Idarubicin and Mitoxantrone among the top 10 compounds. **C** Dose response curves for the patient (red) and 10 patients with RBF-AUC scores closest to the median score for the respective drug. Lines indicate the fit of the dose response curve. Dots indicate blast viability relative to the average of the DMSO control for each replicate after QC. The shaded grey area indicates the standard deviation around the curve fit for the 10 median responders. **D** Barplots of blast percentages at diagnosis and at MRD time points after induction 1 and 2 as determined by flow-cytometry (FCM) indicating complete response after the first induction cycle. **E** Bone marrow smear of sample AML57 (poor responder). The patient had an M2 phenotype, trisomy and mutations in *NRAS*, *WT1*, and *FLT3* **F** Barplots of RBF-AUC scores analogous with induction compounds Daunorubicin, Doxorubicin and the combination of

Cytarabine and Daunorubicin among the lowest scoring drugs analogous to **B**. **G** Dose response curves for top 3 drugs analogous to **C.** **H** Barplots of blast percentages at diagnosis and at MRD time points analogous to **D** indicating highly resistant disease.

Figure S4: Clustering analysis of chemosensitivities

A

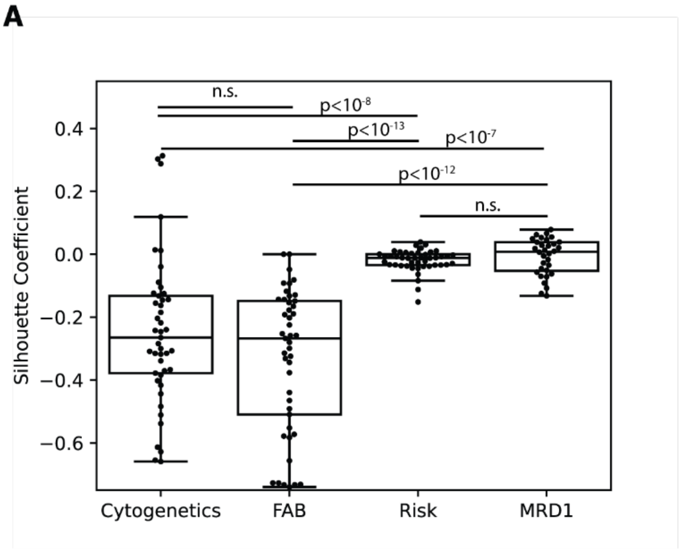

B

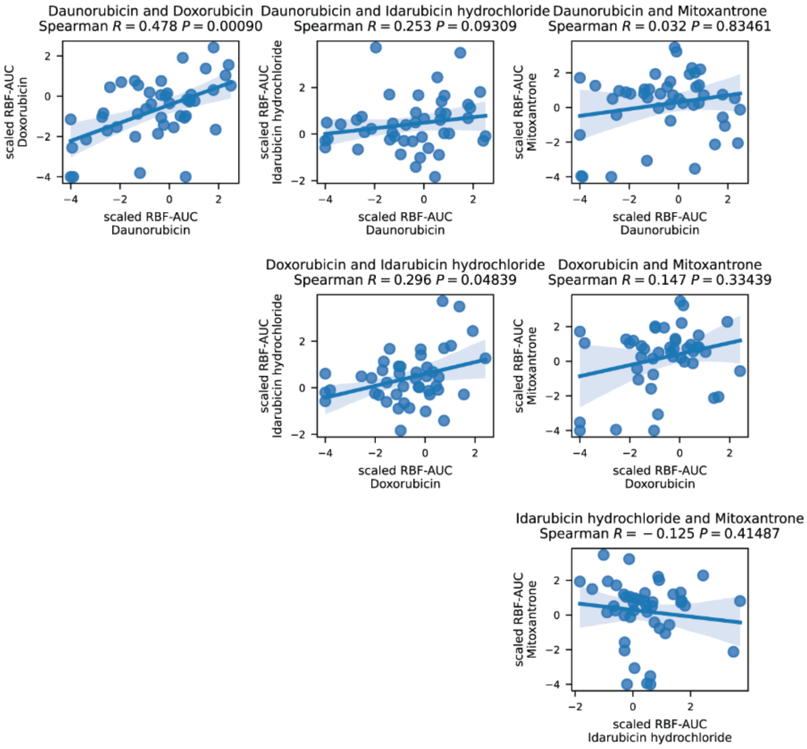

C

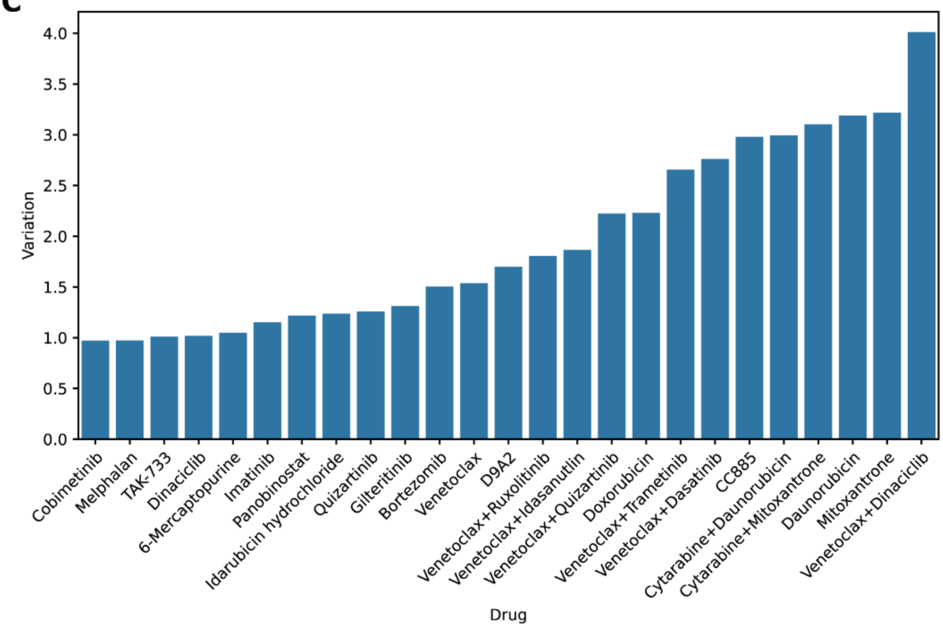

Figure S4: Clustering analysis of chemosensitivities. Related to Figure 3

A Silhouette coefficients of drug sensitivities per sample based on sample classification by cytogenetic group (n=45), FAB classification (n=45), risk group (n=45), and MRD status (n=32) respectively. P-values calculated with Mann-Whitney-U test. Dots indicate individual samples. Boxes represent quartiles. Whiskers extend to points that are within 1.5 interquartile ranges. **B** Correlations of RBF-AUC scores for selected chemotherapeutics for the n=45 samples profiled in this study. Individual dots indicate the scaled RBF-AUC score for individual samples and the respective compounds. Lines indicate a linear model fit. Shaded areas around lines indicate the 95% confidence interval. **C** Variation per compound for the 25 most variable compounds.

Figure S5

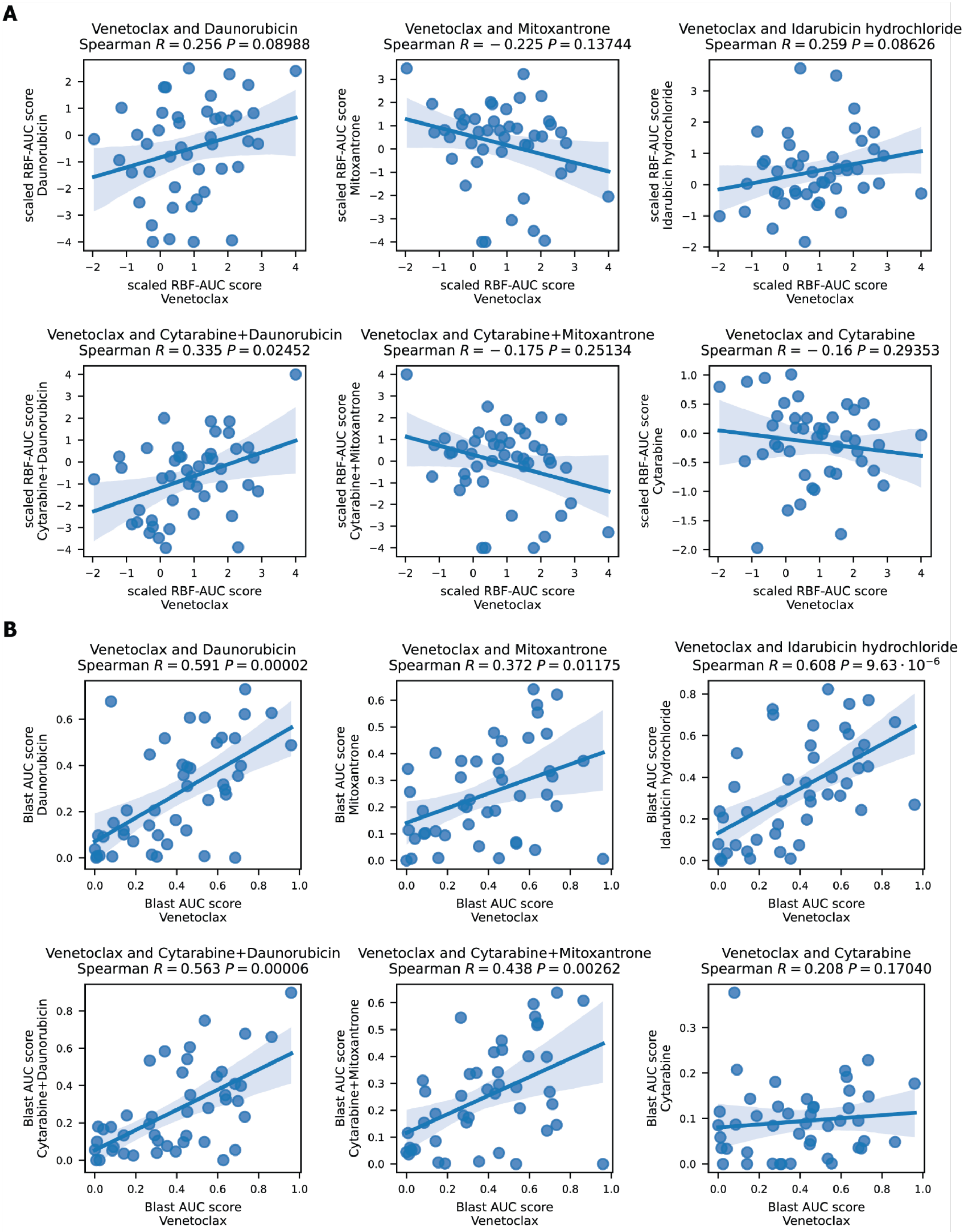

Figure S5: Correlation of Venetoclax and selected chemotherapeutic agents. Related to Figure 3

A Scatterplots and Spearman correlations between Venetoclax on the horizontal axes and selected chemotherapy compounds on the vertical axes for n=45 samples in the cohort. Individual dots indicate the scaled RBF-AUC score for individual samples and

the respective compounds. Lines indicate a linear model fit. Shaded areas around lines indicate the 95% confidence interval. **B** Same as A, but dots indicate the absolute AUC score that only reflects responses of the blast population (Methods).

Figure S6

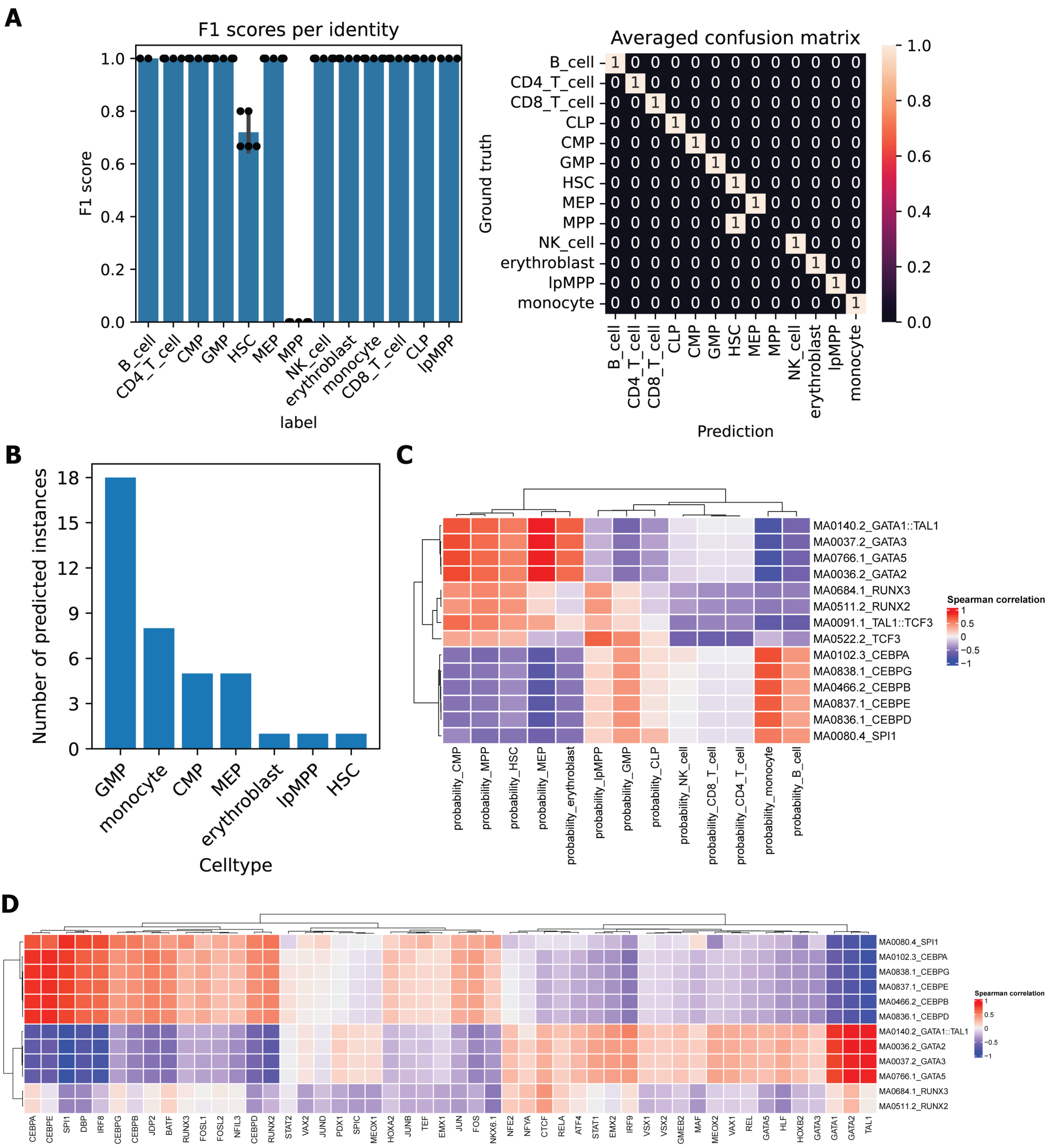

Figure S6: Mapping pedAML samples to their most similar healthy cell-types. Related to Figure 5

A Performance of the support vector classifier. Left: F1 scores per run and celltype in 5-fold cross-validation. Dots indicate F1 scores per run. Bar heights indicate mean F1 scores over all folds. Error bars indicate 95% confidence intervals. Right: Averaged confusion matrix over all runs. **B** Numbers of predicted healthy cell-types for samples in the ExTrAct-cohort. **C** Clustered heatmap of Spearman correlations for predicted probabilities of healthy cell-types and chromVAR deviation scores of key hematopoietic transcription factors **D** Spearman rank correlation for chromVAR scores for TF motifs from Supplementary Figure S4B and normalized expression levels of key hematopoietic transcription factors

Figure S7

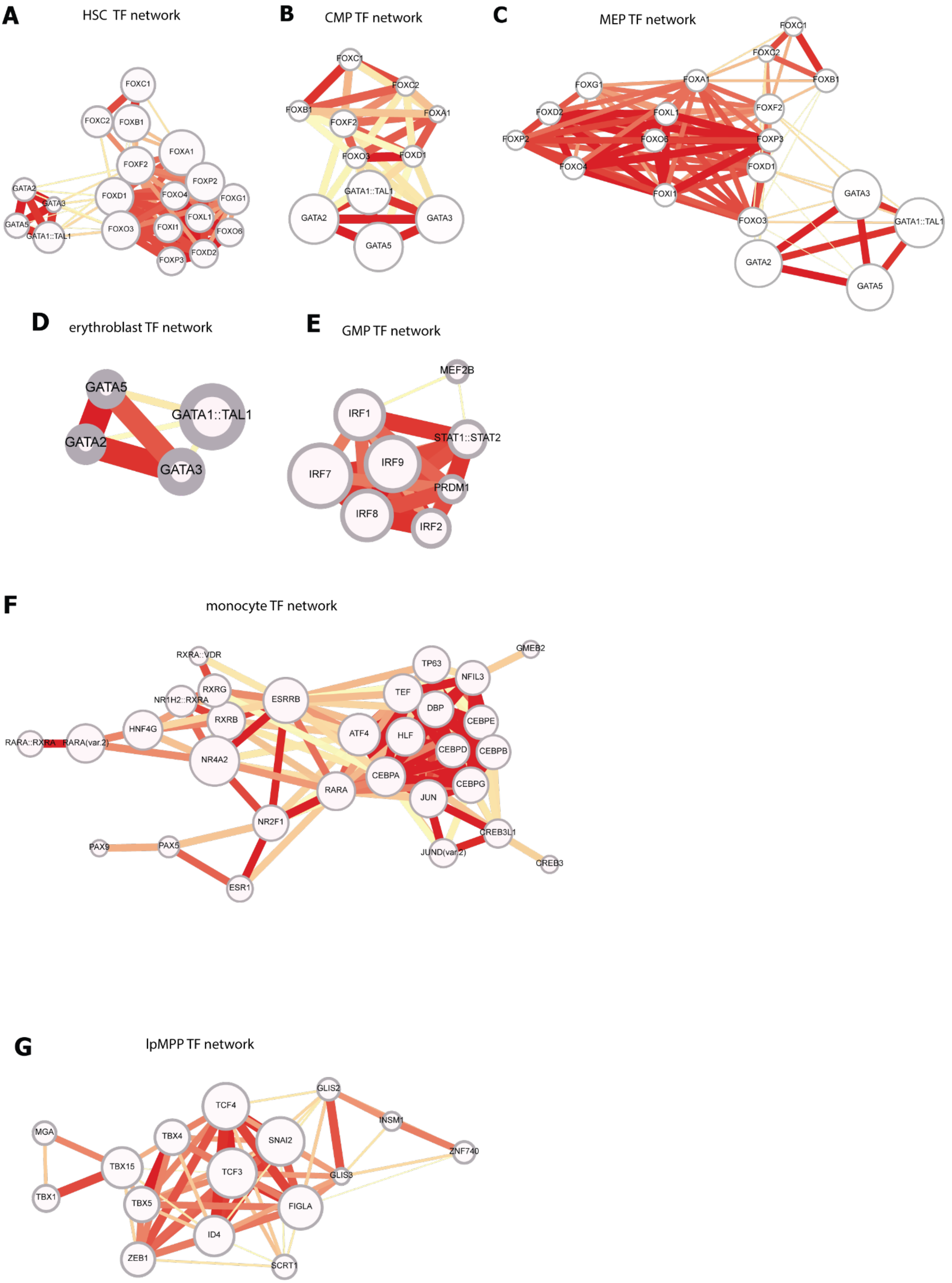

Figure S7: Transcription factor activity networks for predicted cell states. Related to Figure 5

Networks represent the largest connected component correlating TF-activities that are significantly associated with the respective cell states for the n=38 samples with ATAC-seq data after QC. Node sizes are proportional to correlation between TF activity and

cell state probability. Edges represent Spearman correlations of at least 0.8 between individual TF activities (STAR methods). Edge widths and colors are proportional to correlation value. **A** HSC TF network. **B** CMP TF network. **C** MEP TF network. **D** erythroblast TF network. **E** GMP TF network. **F** monocyte TF network. **G** lpMPP TF network.

Figure S8

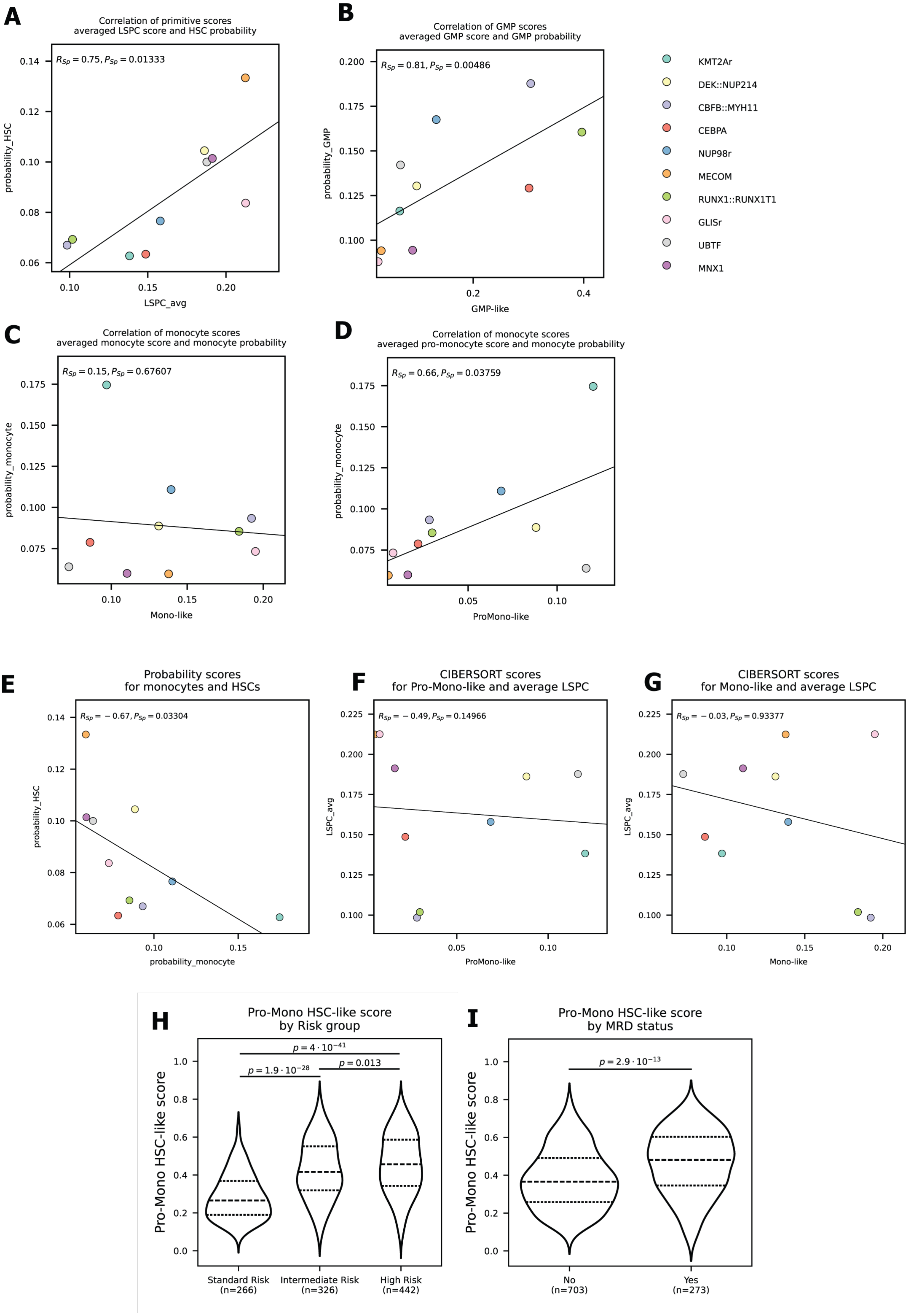

A
Correlation of primitive scores
averaged LSPC score and HSC probability
$R_{Sp} = 0.75, P_{Sp} = 0.01333$
probability_HSC
LSPC_avg
B
Correlation of GMP scores
averaged GMP score and GMP probability
$R_{Sp} = 0.81, P_{Sp} = 0.00486$
probability_GMP
GMP-like
KMT2Ar
DEK::NUP214
CBFB::MYH11
CEBPA
NUP98r
MECOM
RUNX1::RUNX1T1
GLISr
UBTF
MNX1
C
Correlation of monocyte scores
averaged monocyte score and monocyte probability
$R_{Sp} = 0.15, P_{Sp} = 0.67607$
probability_monocyte
Mono-like
D
Correlation of monocyte scores
averaged pro-monocyte score and monocyte probability
$R_{Sp} = 0.66, P_{Sp} = 0.03759$
probability_monocyte
ProMono-like
E
Probability scores
for monocytes and HSCs
$R_{Sp} = -0.67, P_{Sp} = 0.03304$
probability_HSC
probability_monocyte
F
CIBERSORT scores
for Pro-Mono-like and average LSPC
$R_{Sp} = -0.49, P_{Sp} = 0.14966$
LSPC_avg
ProMono-like
G
CIBERSORT scores
for Mono-like and average LSPC
$R_{Sp} = -0.03, P_{Sp} = 0.93377$
LSPC_avg
Mono-like
H
Pro-Mono HSC-like score
by Risk group
$p = 4 \cdot 10^{-41}$
$p = 1.9 \cdot 10^{-28}$
$p = 0.013$
Pro-Mono HSC-like score
Standard Risk (n=266)
Intermediate Risk (n=326)
High Risk (n=442)
I
Pro-Mono HSC-like score
by MRD status
$p = 2.9 \cdot 10^{-13}$
Pro-Mono HSC-like score
No (n=703)
Yes (n=273)

Figure S8: Validation analysis of cell type differentiation states. Related to Figure 5

A-D CIBERSORT scores from Umeda et al (x-axis) plotted against probabilities for cell differentiation states from this study (y-axis). Dots indicate median values per genetic subtype for *KMT2A*r (n=6 this study; n=236 Umeda et al.), *DEK::NUP214* (n=2 this study, n=17 Umeda et al.), *CBFB::MYH11* (n=6 this study, n=102 Umeda et al.), *CEBPA* (n=2 this study, n=63 Umeda et al.), *NUP98*r (n=5 this study, n=77 Umeda et al.), *MECOM* (n=1 this study, n=11 Umeda et al.), *RUNX1::RUNX1T1* (n=1 this study, n=141 Umeda et al.), *GLIS*r (n=1 this study, n=20 Umeda et al.), *UBTF* (n=2 this study, n=45 Umeda et al.), *MNX1* (n=1 this study, n=4 Umeda et al.) Black lines indicate linear model fit regression line. **A** Averaged LSPC CIBERSORT score and HSC probability **B** GMP-like CIBERSORT score and GMP probability **C** Mono-like CIBERSORT score and monocyte probability **D** Pro-Mono like CIBERSORT score and monocyte probability. **E-G** Dots and lines as in A-D. **E** HSC probability plotted against monocyte probability **F** averaged LSPC CIBERSORT score and Mono-like CIBERSORT score **G** averaged LSPC CIBERSORT score and Pro-Mono-like CIBERSORT score **H** Violin plot of Pro-Mono HSC score by AIEOP-BFM risk group **I** Violin plot of Pro-Mono HSC score by MRD status after induction 1. Widths are proportional to the number of samples within the corresponding value interval. Dashed lines in H and I separate the quartiles of the data.

Figure S9

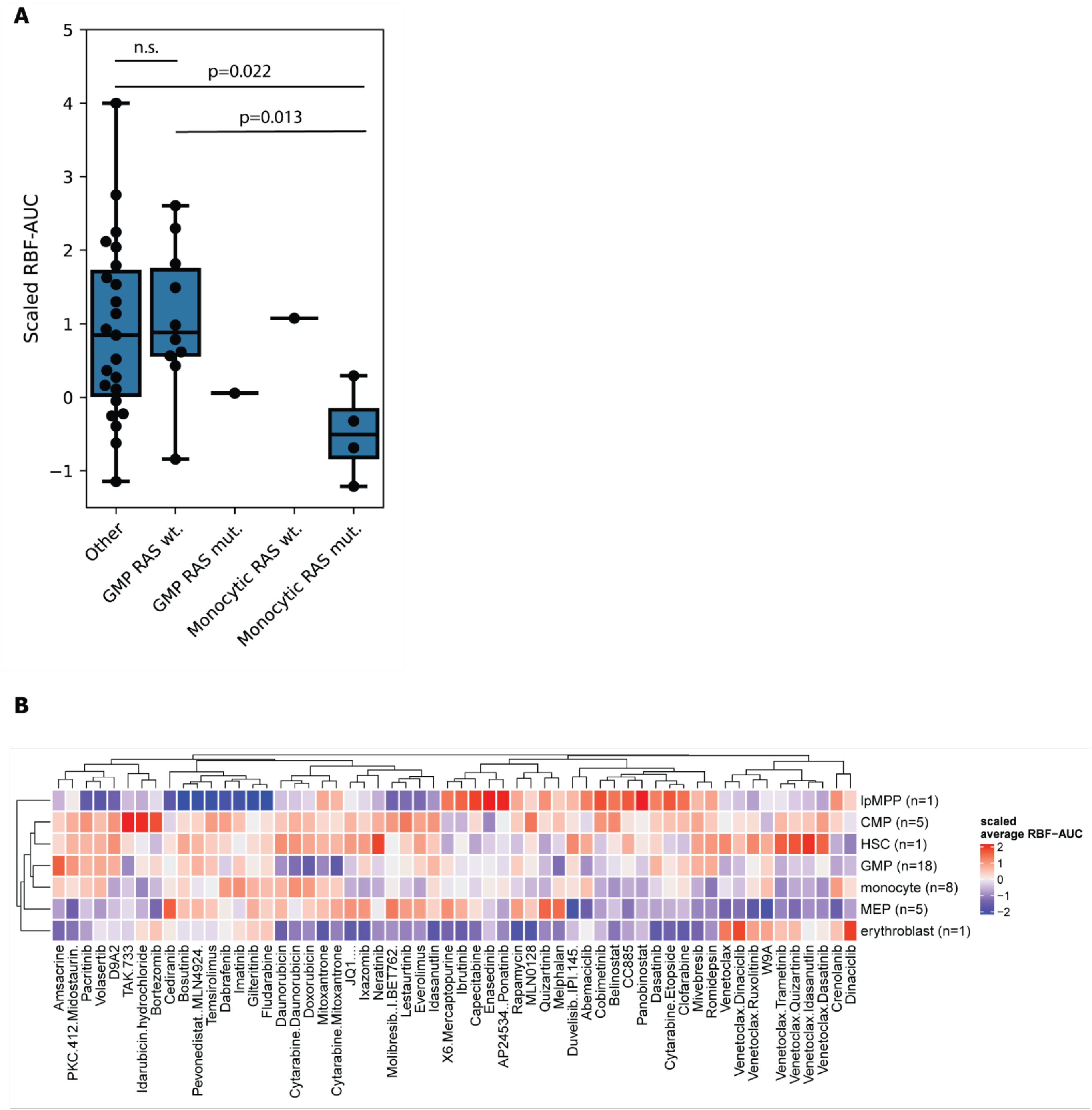

Figure S9: Associations of drug response and cellular differentiation states. Related to Figure 5

A Comparative analysis of Venetoclax response by RAS-mutation status and differentiation status for monocytic samples with mutated RAS (n=4), one monocytic sample with wild type RAS (n=1), one GMP-like sample with mutated RAS (n=1), GMP-like samples with wild type RAS (n=8) and samples that are neither GMP-like nor monocytic and have been termed Other (n=23). P-values were calculated using the Mann-Whitney-U Test. Dots indicate RBF-AUC values for individual samples. Boxes represent quartiles. Whiskers extend to points that are within 1.5 interquartile ranges. **B** Heatmap of scaled averaged RBF-AUC values for predicted differentiation states.

Supplementary Tables

Supplementary Table 1: Compound library overview. Related to Figure 1.

Compound	Compound MoA	Compound class	ConcPoint1 (micromolar)	ConcPoint2 (micromolar)	ConcPoint3 (micromolar)
Pentostatin	Adenosine deaminase inhibitor	Chemotherapeutic	1	0.1	0.01
Thiotepa	Alkylating agent	Chemotherapeutic	1	0.1	0.01
Palifosfamide	Alkylating agent	Chemotherapeutic	1	0.1	0.01
Busulfan	Alkylating agent	Chemotherapeutic	1	0.1	0.01
Melphalan	Alkylating agent	Chemotherapeutic	1	0.1	0.01
Aminopterin	Antimetabolite, Antifolate	Chemotherapeutic	1	0.1	0.01
Pralatrexate	Antimetabolite, Antifolate	Chemotherapeutic	0.7	0.07	0.007
Methotrexate	Antimetabolite, Antifolate	Chemotherapeutic	1	0.1	0.01
Doxorubicin	Antimetabolite, DNA intercalater	Chemotherapeutic	1	0.1	0.01
Idarubicin hydrochloride	Antimetabolite, DNA intercalater	Chemotherapeutic	1	0.1	0.01
Daunorubicin	Antimetabolite, DNA intercalater	Chemotherapeutic	1	0.1	0.01
Mitoxantrone	Antimetabolite, DNA intercalater, Topoisomerase 2 inhibitor	Chemotherapeutic	1	0.1	0.01
Pixantrone	Antimetabolite, DNA intercalater, Topoisomerase 2 inhibitor	Chemotherapeutic	1	0.1	0.01
Etoposide	Antimetabolite, DNA synthesis inhibitor	Chemotherapeutic	2	0.2	0.02
Hydroxyurea	Antimetabolite, DNA sythesis inhibitor, other effects	Chemotherapeutic	1	0.1	0.01
Amsacrine	Antimetabolite, DNA-intercalater	Chemotherapeutic	1	0.1	0.01
Thioguanine	Antimetabolite, Purines	Chemotherapeutic	1	0.1	0.01
6-Mercaptopurine	Antimetabolite, Purines	Chemotherapeutic	0.1	0.01	0.001
Clofarabine	Antimetabolite, Purines	Chemotherapeutic	1	0.1	0.01
Fludarabine	Antimetabolite, Purines	Chemotherapeutic	1	0.1	0.01
5-Fluorouracil	Antimetabolite, Pyrimidines	Chemotherapeutic	1	0.1	0.01
Cytarabine	Antimetabolite, Pyrimidines	Chemotherapeutic	1	0.1	0.01
Elacytarabine	Antimetabolite, Pyrimidines	Chemotherapeutic	1	0.1	0.01
Capecitabine	Antimetabolite, Pyrimidines	Chemotherapeutic	1	0.1	0.01
5-Azacytidine	Antimetabolite, Pyrimidines, Hypomethylating agent	Chemotherapeutic	3	0.3	0.03
Irinotecan	Antimetabolite, Topoisomerase 1 inhibitor	Chemotherapeutic	1	0.1	0.01
Venetoclax	BCL2-inhibitor	Cell death signaling (+/- Kinase inhibitor)	1	0.1	0.01
Molibresib (I-BET762)	BET inhibitor	Epigenetic	1	0.1	0.01
JQ1-(+)	BET inhibitor	Epigenetic	0.96	0.096	0.0096
Mivebresib	BET inhibitor	Epigenetic	1	0.1	0.01
Venetoclax+Dasatinib	Combination	Cell death signaling (+/- Kinase inhibitor)	0.5+0.5	0.05+0.05	0.005+0.005
Venetoclax+Ruxolitinib	Combination	Cell death signaling (+/- Kinase inhibitor)	0.5+0.5	0.05+0.05	0.005+0.005
Venetoclax+Idasanutlin	Combination	Cell death signaling (+/- Kinase inhibitor)	0.5+0.5	0.05+0.05	0.005+0.005
Venetoclax+Dinaciclib	Combination	Cell death signaling (+/- Kinase inhibitor)	0.5+0.5	0.05+0.05	0.005+0.005
Venetoclax+Trametinib	Combination	Cell death signaling (+/- Kinase inhibitor)	0.5+0.5	0.05+0.05	0.005+0.005

Venetoclax+Quizartinib	Combination	Cell death signaling (+/- Kinase inhibitor)	0.5+0.5	0.05+0.05	0.005+0.005
Cytarabine+Daunorubicin	Combination, Antimetabolite	Chemotherapeutic	0.5+0.5	0.05+0.05	0.005+0.005
Cytarabine+Etopside	Combination, Antimetabolite	Chemotherapeutic	0.5+0.5	0.05+0.05	0.005+0.005
Cytarabine+Mitoxantrone	Combination, Antimetabolite	Chemotherapeutic	0.5+0.5	0.05+0.05	0.005+0.005
Pinemetostat (EPZ-5676)	Epigenetic drug	Epigenetic	1	0.1	0.01
Romidepsin	Epigenetic drug, HDAC inhibitor	Epigenetic	2	0.2	0.02
Belinostat	Epigenetic drug, HDAC inhibitor	Epigenetic	1	0.1	0.01
Panobinostat	Epigenetic drug, HDAC inhibitor	Epigenetic	5	0.5	0.05
ORY1001	Epigenetic drug, Histone demethylase inhibitor	Epigenetic	1	0.1	0.01
Tazemetostat (EPZ-6438)	Epigenetic drug, Histone methyltransferase inhibitor	Epigenetic	1	0.1	0.01
Guadecitabine	Epigenetic drug, hypomethylating agent	Epigenetic	1	0.1	0.01
Decitabine	Epigenetic drug, hypomethylating agent	Epigenetic	1	0.1	0.01
GSK126	Epigenetic drug, Methyltransferase inhibitor	Epigenetic	1	0.1	0.01
Prednisolone	glucocorticoid	Other	1	0.1	0.01
Vismodegib	Hedgehog pathway inhibitor	Kinase inhibitor	2	0.2	0.02
Glasdegib	Hedgehog pathway inhibitor	Kinase inhibitor	1	0.1	0.01
Ivosidenib	IDH inhibitor	Metabolic drug	1	0.1	0.01
Enasedinib	IDH inhibitor	Metabolic drug	1	0.1	0.01
Ceritinib	Kinase inhibitor, ALK	Kinase inhibitor	1	0.1	0.01
Crizotinib	Kinase inhibitor, ALK	Kinase inhibitor	2	0.2	0.02
Alisertib	Kinase inhibitor, Aurora	Kinase inhibitor	1	0.1	0.01
Bafetinib	Kinase inhibitor, BCR-ABL	Kinase inhibitor	1	0.1	0.01
Imatinib	Kinase inhibitor, BCR-ABL	Kinase inhibitor	1	0.1	0.01
AP24534 Ponatinib	Kinase inhibitor, BCR-ABL	Kinase inhibitor	1	0.1	0.01
Dasatinib	Kinase inhibitor, BCR-ABL	Kinase inhibitor	1	0.1	0.01
Nilotinib	Kinase inhibitor, BCR-ABL	Kinase inhibitor	2	0.2	0.02
Bosutinib	Kinase inhibitor, BCR-ABL	Kinase inhibitor	1	0.1	0.01
Vemurafenib	Kinase inhibitor, BRAF	Kinase inhibitor	2	0.2	0.02
Dabrafenib	Kinase inhibitor, BRAF	Kinase inhibitor	1	0.1	0.01
Ibrutinib	Kinase inhibitor, BTK	Kinase inhibitor	1	0.1	0.01
Dinaciclib	Kinase inhibitor, CDK	Kinase inhibitor	1	0.1	0.01
Palbociclib	Kinase inhibitor, CDK	Kinase inhibitor	1	0.1	0.01
Abemaciclib	Kinase inhibitor, CDK	Kinase inhibitor	1	0.1	0.01
Neratinib	Kinase inhibitor, EGFR	RTK inhibitor	1	0.1	0.01
Erlotinib	Kinase inhibitor, EGFR	RTK inhibitor	2	0.2	0.02
Gefitinib	Kinase inhibitor, EGFR	RTK inhibitor	1	0.1	0.01
Gilteritinib	Kinase inhibitor, FLT3	RTK inhibitor	0.1	0.01	0.001
Quizartinib	Kinase inhibitor, FLT3	RTK inhibitor	1	0.1	0.01
Crenolanib	Kinase inhibitor, FLT3	RTK inhibitor	1	0.1	0.01
Lestaurtinib	Kinase inhibitor, FLT3	RTK inhibitor	1	0.1	0.01
Ruxolitinib	Kinase inhibitor, JAK	Kinase inhibitor	1	0.1	0.01
Tofacitinib	Kinase inhibitor, JAK	Kinase inhibitor	1	0.1	0.01
Pacritinib	Kinase inhibitor, JAK	Kinase inhibitor	1	0.1	0.01
Trametinib	Kinase inhibitor, MEK	Kinase inhibitor	1	0.1	0.01
Cobimetinib	Kinase inhibitor, MEK	Kinase inhibitor	1	0.1	0.01

TAK-733	Kinase inhibitor, MEK	Kinase inhibitor	1	0.1	0.01
Idelalisib	Kinase inhibitor, PI3K	Kinase inhibitor	1	0.1	0.01
Duvelisib (IPI-145)	Kinase inhibitor, PI3K	Kinase inhibitor	1	0.1	0.01
Volasertib	Kinase inhibitor, Plk	Kinase inhibitor	1	0.1	0.01
Sunitinib	Kinase inhibitor, RTK	RTK inhibitor	1	0.1	0.01
Nintedanib	Kinase inhibitor, RTK	RTK inhibitor	1	0.1	0.01
Cabozantinib	Kinase inhibitor, unspecific	RTK inhibitor	2	0.2	0.02
Regorafenib	Kinase inhibitor, unspecific	Kinase inhibitor	2	0.2	0.02
PKC-412(Midostaurin)	Kinase inhibitor, unspecific	RTK inhibitor	1	0.1	0.01
Sorafenib	Kinase inhibitor, unspecific	Kinase inhibitor	1	0.1	0.01
Vandetanib	Kinase inhibitor, VEGFR	RTK inhibitor	2	0.2	0.02
Semaxanib	Kinase inhibitor, VEGFR	RTK inhibitor	1	0.1	0.01
Pazopanib	Kinase inhibitor, VEGFR	RTK inhibitor	0.443	0.0443	0.00443
Cediranib	Kinase inhibitor, VEGFR	RTK inhibitor	1	0.1	0.01
Idasanutlin	MDM2 inhibitor	Cell death signaling (+/- Kinase inhibitor)	1	0.1	0.01
Temsirolimus	mTOR inhibitor	Metabolic drug	1	0.1	0.01
Everolimus	mTOR inhibitor	Metabolic drug	1	0.1	0.01
Rapamycin	mTOR inhibitor	Metabolic drug	1	0.1	0.01
MLN0128	mTOR inhibitor	Metabolic drug	1	0.1	0.01
MLN2480	mTOR inhibitor	Metabolic drug	1	0.1	0.01
Pevonedistat (MLN4924)	NEDD inhibitor	Metabolic drug	1	0.1	0.01
Veliparib	PARP inhibitor	Epigenetic	1	0.1	0.01
Olaparib	PARP inhibitor	Epigenetic	1	0.1	0.01
Carfilzomib	Proteasome inihibitor	Metabolic drug	0.000117	0.0000117	0.00000117
Ixazomib	Proteasome inihibitor	Metabolic drug	1	0.1	0.01
Bortezomib	Proteasome inihibitor	Metabolic drug	1	0.1	0.01
Bexarotene	Retinoid	Other	2	0.2	0.02
Alitretinoin	Retinoid receptor activator	Other	2	0.2	0.02
CC885	SLC9A1 degradation toxicitiy control	Other	1	0.1	0.01
D9A2	SLC9A1 degrader	Metabolic drug	1	0.1	0.01
W9A	SLC9A1 inhibitor	Metabolic drug	1	0.1	0.01
Rosuvastatin	Statin	Metabolic drug	1	0.1	0.01
Lovastatin	Statin	Metabolic drug	1	0.1	0.01
Telaglenastat (CB-839)	TCA cycle inhibitor, targets Glutaminase	Metabolic drug	1	0.1	0.01
Devimistat	TCA cycle inhibitor, targets Pyruvate Dehydrogenase	Metabolic drug	1	0.1	0.01

Supplementary Table 2: Extended patient information. Related to Figure 2.

Extended information on patient samples in the cohort. (WES) next do detected mutations in the Additional alterations column indicates that the alterations have been identified via whole-exome sequencing. Abbreviations are the following: NK: Normal Karyotype; WES: Whole exome sequencing; ND: not detected/not assessed; Dx: Diagnosis; SCT: stem cell transplantation; FUP: follow up; MRD: Measurable residual disease; SR: Standard risk; IR: Intermediate risk; HR: High risk; pos: positive; neg: negative

Sample ID	Sex	Age at Dx (years)	FAB	Cytogenetics	Additional alterations	FCM-MRD (d21 or d28)	PCR MRD d28	MRD vote Ind1	FCM-MRD d56	PCR MRD d56	MRD vote Ind2	Risk BFM 2019	RISK BFM 2019 post Ind1	Time to death (days)	Time to relapse (days)	Time to SCT (days)	Time to FUP (days)	Treatment _protocol	Umeda 2024 category
AML74	male	17,8	M4	NK	FLT3-ITD	7.50 %	ND	pos	ambiguous	nd	neg	IR	HR	745	303	no SCT	745	AML-BFM 04 mod	UBTF
AML20	female	3,8	M1	NK	FLT3-ITD NPM1	0.53 %	ND	pos	ambiguous	nd	neg	IR	HR	alive	no relapse	no SCT	603	AML-BFM 2012	NPM1
AML82	female	16,5	M1	NK		neg	ND	neg	neg	neg	neg	IR	IR	alive	798	no SCT	1358	AML-BFM 2013	CBFB-GDXY
AML64	male	5,7	M1	NK		neg	ND	neg	neg	nd	neg	IR	IR	alive	no relapse	no SCT	1868	AML-BFM 2004	unclassified
AML31	male	11,8	M7	BCR::ABL1		ND	ND	ND	nd	nd	ND	HR	HR	alive	no relapse	139	2230	AML04 Interim	BCR-ABL1
AML93	female	7,4	M0	ETV6::MNX1		ND	ND	ND	nd	nd	ND	HR	HR	668	480	100	668	AML04 Interim	MNX1
AML37	male	1,4	M5a	KMT2A::MLLT1		neg	<0,01 %	neg	neg	neg	neg	IR	IR	alive	no relapse	no SCT	1091	AML-BFM 2012	KMT2Ar
AML06	male	1,4	M7	NUP98::KDM5A		10.60%	2%	pos	0.104%	0.09%	pos	HR	HR	254	154	no SCT	254	AML-BFM 2012	NUP98r AMKL
AML57	male	18,3	M2/MDS!	trisomy 8	NRAS, WT1	20%	ND	pos	70%	nd	pos	IR	HR	alive	no relapse	67	246	AML-BFM 2004	UBTF
AML84	female	1,4	M7	complex		neg	ND	neg	neg	nd	neg	HR	HR	alive	no relapse	no SCT	607	AML-BFM 2012	unclassified
AML13	male	17,7	M2	KMT2A::MLLT1	FLT3-ITD, DNMT3A, RAD21	0.09 %	0.70%	pos	neg	<0,01 %	neg	IR	HR	alive	326	625	938	AML-BFM 2012	KMT2Ar
AML39	female	0,1	M4	DEK::NUP214	FLT3-ITD	3.10 %	ND	pos	2.20%	nd	pos	HR	HR	alive	no relapse	134	322	AML-BFM 2012	DEK-NUP214
AML44	female	12,8	M7	NUP98::KDM5A	KIT	0.38 %	ND	pos	neg	nd	neg	HR	HR	alive	no relapse	no SCT	911	AML-BFM 2012	NUP98r AMKL

AML66	female	8,9	M4Eo	CBFB::MYH11		neg	ND	neg	neg	nd	neg	SR	SR	alive	no relapse	no SCT	1207	AML-BFM 2012	CBFB-MYH11
AML47	male	17,4	M4Eo	CBFB::MYH11	NRAS, KIT	ambiguous	ND	neg	ambiguous	nd	neg	SR	SR	alive	no relapse	no SCT	237	AML-BFM 2012	CBFB-MYH11
AML58	male	4,4	M2	NK	CEBPAdm	neg	nd	neg	neg	nd	neg	SR	SR	alive	no relapse	no SCT	555	AML-BFM 2012	CEBPA
AML63	male	4,6	M5a	NUP98::NSD1	FLT3-ITD, GATA2	ambiguous	ND	neg	ambiguous	nd	neg	HR	HR	alive	973	973	1635	AML-BFM 04	NUP98r
AML62	female	14,2	M4	DEK::NUP214	FLT3-ITD	nd	ND	ND	neg	nd	neg	HR	HR	alive	no relapse	134	1362	AML-BFM 2004	DEK-NUP214
AML59	male	7,6	M4Eo	CBFB::MYH11		0.10 %	ND	pos	neg	nd	neg	SR	IR	alive	no relapse	no SCT	2313	AML04 Interim	CBFB-MYH11
AML77	male	8,5	M4	trisomy 8	GATA2	1.50 %	ND	pos	0.36%	nd	pos	IR	HR	66	no relapse	63	66	AML-BFM 04	unclassified
AML26	female	6,1	M4Eo	CBFB::MYH11		ND	ND	ND	nd	nd	ND	SR	SR	alive	no relapse	no SCT	2457	AML04 Interim	CBFB-MYH11
AML88	male	5,1	M5	monosomy 7	NRAS	neg	ND	neg	neg	nd	neg	HR	HR	alive	no relapse	32	2190	AML-BFM 2004	unclassified
AML95	male	15,4	M4	inv(3)(q21q26) RPN1/MECOM		ND	ND	ND	38%	nd	pos	HR	HR	319	190	93	319	AML-BFM 04	MECOM
AML54	male	0,1	M5	KMT2A::MLLT3		ambigous 0,17 %	<0,01 %	neg	0.01%	0.03%	neg	IR	IR	342	237	175	342	AML-BFM 2012	KMT2Ar
AML07	female	15,3	M5b	KMT2A::MLLT3		neg	0.20%	pos	neg	<0,01 %	neg	IR	HR	alive	no relapse	no SCT	979	AML-BFM 2012	KMT2Ar
AML60	male	9,8	M4Eo	CBFB::MYH11		neg	nd	neg	neg	nd	neg	SR	SR	alive	1069	1175	1667	AML04 Interim	CBFB-MYH11
AML98	female	1,4	M4Eo	CBFB::MYH11		ND	ND	ND	neg	nd	neg	SR	SR	alive	no relapse	no SCT	2343	AML04 Interim	CBFB-MYH11
AML89	female	4,5	M2	RUNX1::RUNX1T1		ND	ND	ND	nd	nd	ND	SR	SR	alive	no relapse	no SCT	3499	AML04 Interim	RUNX1-RUNX1T1
AML76	male	16,8	M5	KMT2A::MLLT3		neg	neg	neg	neg	neg	neg	IR	IR	alive	no relapse	no SCT	1583	AML-BFM 2004	KMT2Ar
AML87	male	8,9	M7	NK	ETV6, JAK3, WT1	0.54 %	0.30%	pos	neg	neg	neg	IR	HR	949	413	no SCT	949	AML-BFM 2012	unclassified
AML50	male	3,3	M7	CBFA2T3::GLIS2	GATA2	3.07 %	4%	pos	neg	neg	neg	HR	HR	alive	no relapse	no SCT	1368	AML-BFM 2012	GLISr
AML52	female	11	M5	KMT2A::MLLT10		neg	<0,01 %	neg	neg	neg	neg	HR	HR	alive	no relapse	153	956	AML-BFM 2012	KMT2Ar

AML65	female	11,3	M5	KMT2A::MLLT3	NRAS	neg	neg	neg	neg	neg	neg	IR	IR	alive	no relapse	no SCT	410	AML-BFM 2012	KMT2Ar
AML23	female	7,2	M5a	KMT2A::MLLT1	GATA2, KRAS, NRAS	0.36 %	0.20%	pos	neg	neg	neg	IR	HR	alive	293	no SCT	1441	AML-BFM 2012	KMT2Ar
AML67	female	1,1	M5a	KMT2A::MLLT3		neg	<10-4	neg	neg	neg	neg	IR	IR	alive	no relapse	no SCT	1730	AML-BFM 2004	KMT2Ar
AML28	female	16,4	M1	NK	FLT3, NRAS, WT1 (WES)	ND	ND	ND	ND	ND	ND	IR	IR	472	314	394	472	AML-BFM 2004	unclassified
AML24	male	10,3	M4	trisomy 8	FLT3-ITD, UBTF, (WES)	ND	ND	ND	ND	ND	ND	IR	IR	alive	421	519	2191	(AML-BFM 04) different therapy	UBTF
AML43	male	17,8	M2	NK	CEBPAdm (WES)	ND	ND	ND	ND	ND	ND	SR	SR	alive	no relapse	no SCT	1735	AML-BFM 2004	CEBPA
AML04	male	6,7	M1	NK	FLT3-ITD	ND	ND	ND	ND	ND	ND	IR	IR	534	277	357	534	AML-BFM 2004	unclassified
AML81	female	12,6	M4	other	FLT3 (WES)	ND	ND	ND	ND	ND	ND	IR	IR	alive	no relapse	no SCT	2961	AML-BFM 2004	unclassified
AML17	female	16,9	M4	NUP98::NSD1	FLT3	ambigous	1%	pos	ambiguous	0.10%	pos	HR	HR	alive	no relapse	137	137	AML-BFM 2012	NUP98r
AML12	male	10,8	M2	NUP98::NSD1	NRAS	32.71%	50%	pos	neg	0.04%	neg	HR	HR	alive	463	159	463	AML-BFM 2012	NUP98r
AML29	female	3,9	M2	NUP98::NSD1		ND	ND	ND	ND	ND	ND	HR	HR	alive	397	146	4190	(AML-BFM 04) different therapy	NUP98r
AML55	male	7,6	M5	NUP98::KDM5A		neg	<0,01 %	neg	neg	neg	neg	HR	HR	alive	no relapse	181	543	AML-BFM recommendations 2019	NUP98r
AML34	male	1,7	M6/M7	NUP98::KDM5A		0.21 %	0.40%	pos	0.34%	0.30%	pos	HR	HR	alive	no relapse	no SCT	258	AML-BFM recommendations 2019	NUP98r AMKL
